# Supplementary material for: NR2F1 regulates regional progenitor dynamics in the mouse neocortex and cortical gyrification in BBSOAS patients
Source: EMBO J. 2020 Jun 2;39(13):e104163. doi: 10.15252/embj.2019104163 (PMC7327499; doi:10.15252/embj.2019104163)
Supplement: Supplementary file 1 — Appendix [file EMBJ-39-e104163-s001.pdf]

Altered regional progenitor dynamics in the neocortex of a BBSOA disease mouse model

Bertacchi *et al.*

APPENDIX FIGURES/TABLES and LEGENDS
SUPPLEMENTARY METHODS

Table of contents:

- **Appendix Figure S1.**
  Nr2f1 regulates cell proliferation in vitro.
- **Appendix Figure S2.**
  Flow cytometry analysis of Nr2f1-mediated control of NP proliferation in the posterior mouse cortex.
- **Appendix Figure S3.**
  Neurogenic gradient, cell cycle dynamics and Nr2f1 levels along the latero-medial axis of mouse neocortex.
- **Appendix Figure S4.**
  Nr2f1-mediated control of Pax6 level in neural progenitors.
- **Appendix Figure S5.**
  CRISPR/Cas9-mediated downregulation of NR2F1 expression in mouse cortical cells.
- **Appendix Figure S6.**
  Hierarchical gene clustering analysis of E15.5 Nr2f1 WT and KO neocortices.
- **Supplementary Methods.**
  Detailed experimental materials and methods, with supplementary references.
- **Appendix Table S1.**
  Complete list of differentially expressed genes (RNA-Seq).
- **Appendix Table S2.**
  Gene Ontology (GO) analysis by DAVID software.
- **Appendix Table S3.**
  GO analysis by IPA (Ingenuity Pathway Analysis).
- **Appendix Table S4.**
  GO analysis by PANTHER Overrepresentation Test.
- **Appendix Table S5.**
  Complete list of P-values.
- **Appendix Table S6.**
  List of real time RT-PCR primers.

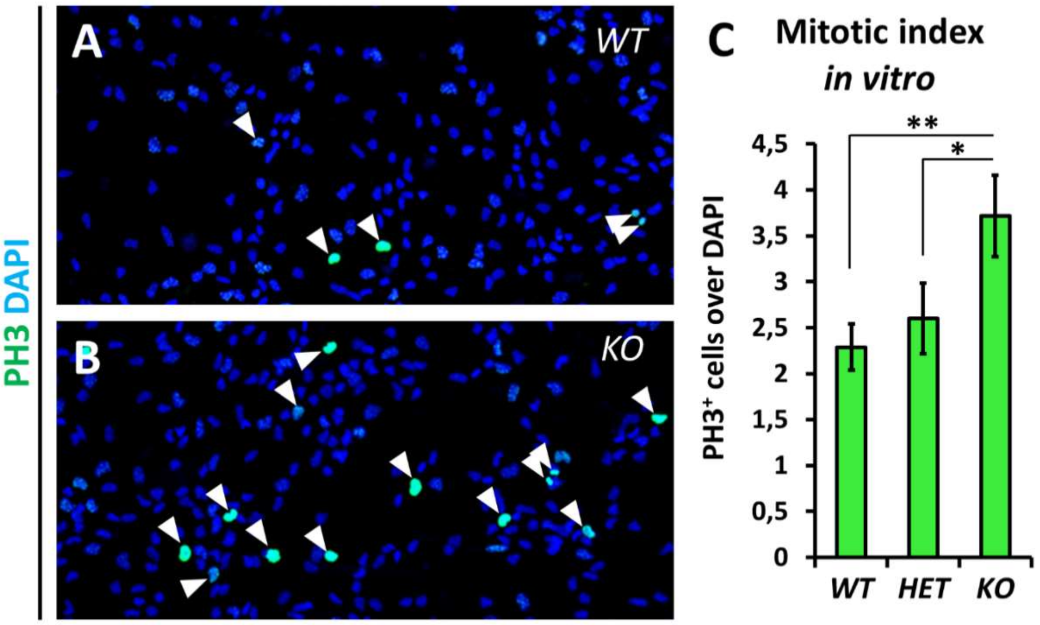

Appendix Figure S1. Nr2f1 regulates cell proliferation in vitro. (**A,B**) PH3 (green; mitotic figures) immunofluorescence (IF) of *WT* (A) and *Nr2f1 KO* (B) NP cells, cultured *in vitro* as a monolayer in the presence of FGF2 and EGF. PH3+ dividing cells are highlighted by white arrowheads. (**C**) PH3+ mitotic figure percentages in *WT*, *HET* and *KO* cells. Nuclei (blue) were stained with DAPI. Data are represented as means ± SEM. 2-way ANOVA (C; *P<0.05).

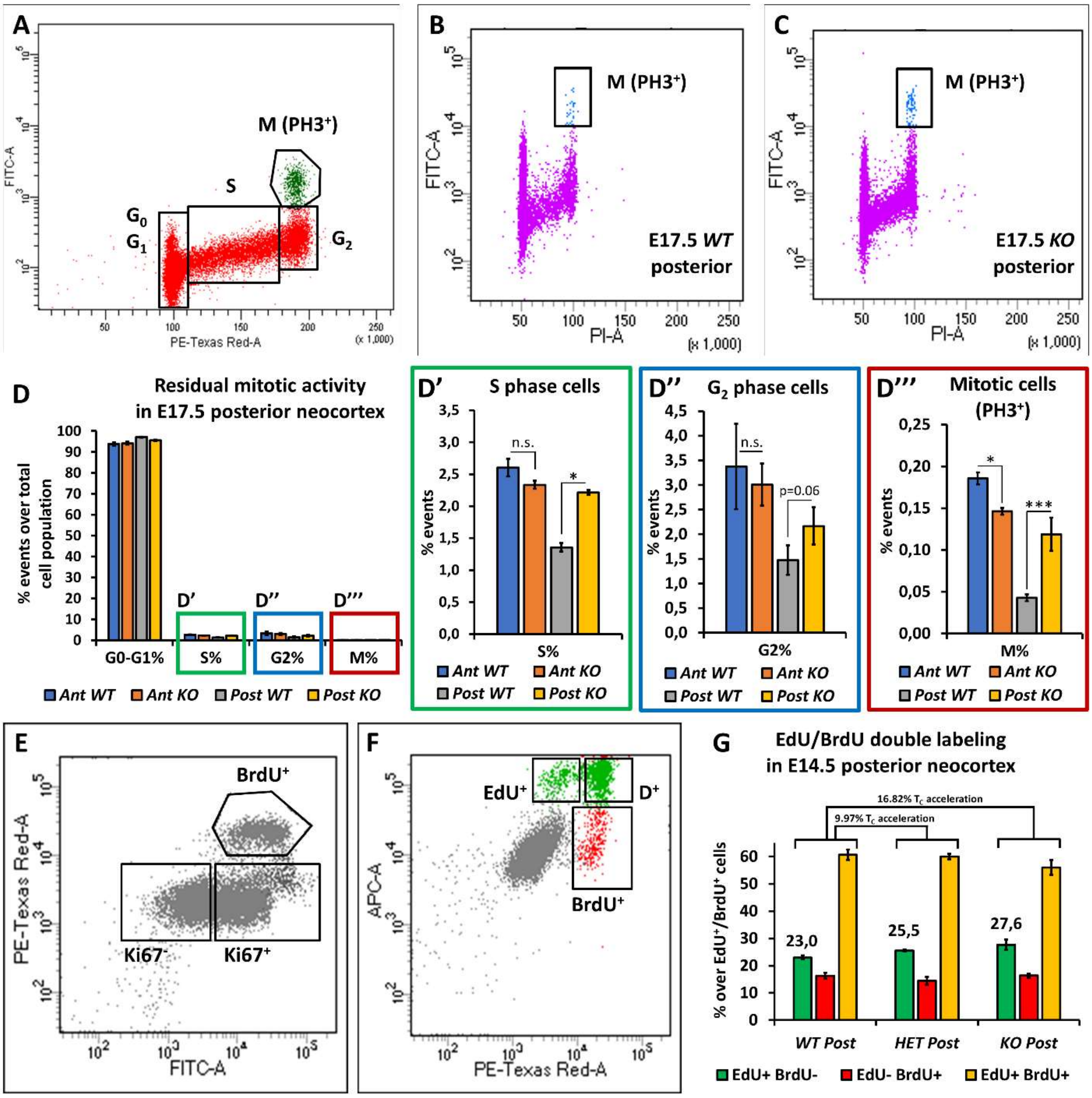

Appendix Figure S2. Flow cytometry analysis of Nr2f1-mediated control of NP proliferation in the posterior mouse cortex. (**A**) Cell cycle phase analysis of cycling NPs after staining with Propidium Iodide (Texas red channel, x-axis) to measure DNA content (G_0/G_1, S and G_2 phases) and with anti-PH3 antibody (FITC channel; y-axis) to label M-phase cells (green dots). (**B,C**) Cell cycle phase analysis of *WT* (B) and *Nr2f1 KO* (C) E17.5 cortical NPs. Single cells were obtained from dissected posterior cortices. Note the residual mitotic activity in mutant cortices (blue dots). (**D-D'''**) Graphs showing the percentage of NPs in different cell cycle phases (G_0-G_1; S; G_2 and M) in *WT versus KO* and in different areas (anterior -Ant- or posterior -Post- cortices), as indicated. Graphs (D'), (D'') and (D''') help to appreciate percentages of cells in S, G_2 and M phases, respectively. *KO* cortices have a higher number of S, G_2- and M-phase NPs, compared to *WT* in posterior brains. (**E-G**) Cell proliferation analysis after double BrdU and EdU (red) injection at E14.5, as schematized in Figure EV5D. Ki67 IF (FITC channel; x-axis in E) allows to only evaluate proliferating cells. BrdU (Texas red channel; y-axis in E and x-axis in F) and EdU (APC channel; y-axis in F) detection allows to quantify the leaving fraction (EdU^+BrdU^-) and the total number of EdU^+BrdU^+ or $BrdU^+$ labelled cells (S-phase cells). Percentages are shown in (G). The leaving fraction increases from 23% (*WT*) to 25.5% and 27.6% (*HET* and *KO*, respectively), indicating a 9.97% increase of cell cycle speed in *HET* and a 16.82% increase in *KO* NPs. Data are represented as means ± SEM. 2-way ANOVA (Graphpad; D-D'''; *P<0.05, ***P<0.001).

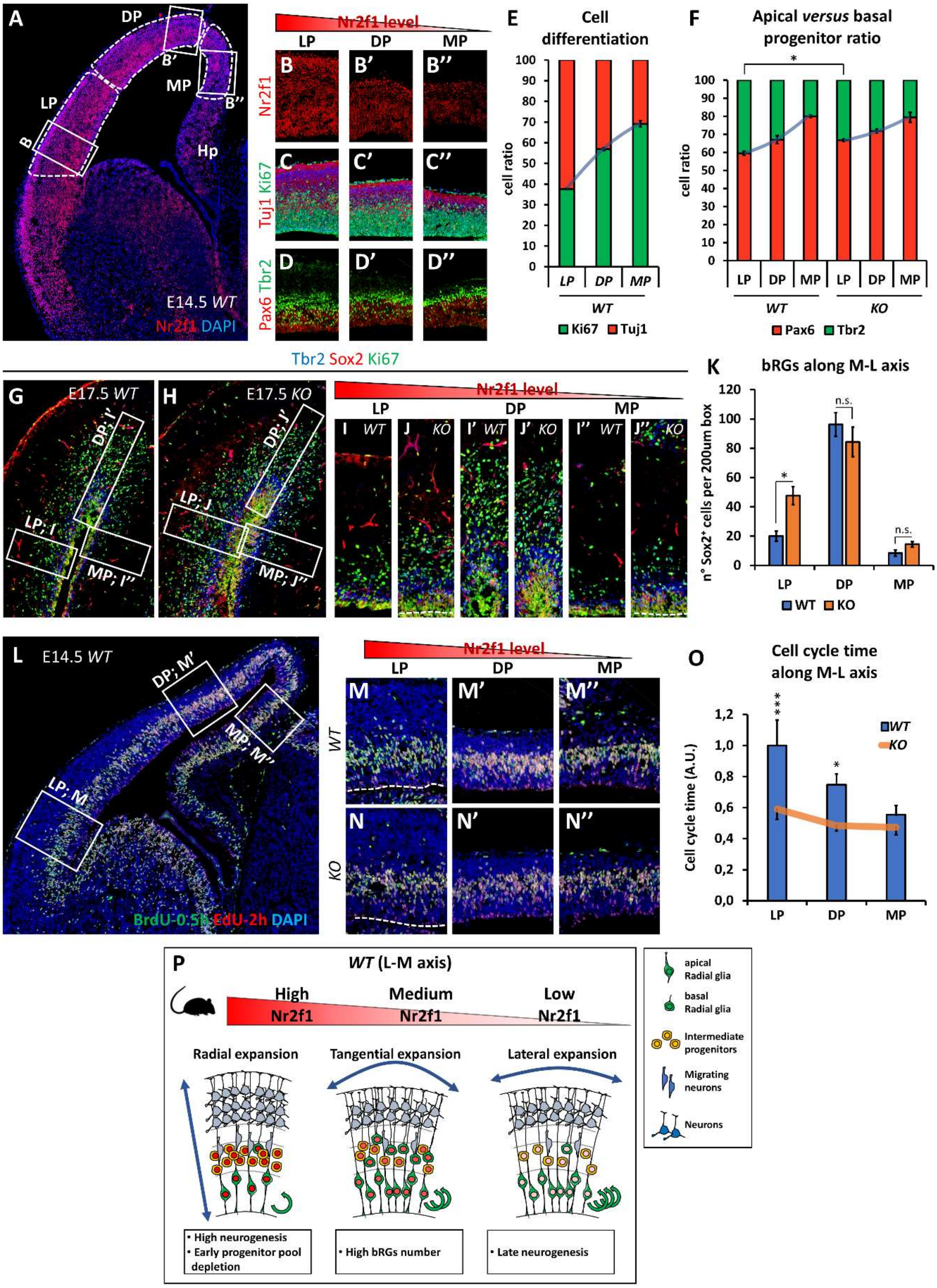

Appendix Figure S3. Neurogenic gradient, cell cycle dynamics and Nr2f1 levels along the latero-medial axis of mouse neocortex. (**A-B''**) Nr2f1 (red) IF of E14.5 *WT* brain, showing high (LP in B) to low levels (MP in B'') of expression. DP (B') has intermediate levels. (**C-C''**) Tuj1 (red) and Ki67 (green) IF in lateral (C), dorsal (C') and medial (C'') pallia of *WT* animals. Neurogenesis starts earlier in the LP than in the MP, as seen by accumulating Tuj1^{+} neurons in the CP. (**D-D''**) Pax6 (red) and Tbr2 (green) IF in LP (D), DP (D') and MP (D'') of *WT* animals. Appearance of Tbr2^{+} IPs correlates with higher neurogenesis in LP regions compared to dorso-medial ones. (**E**) Graph indicating the percentage of Ki67^{+}

NPs (green) and Tuj1+ neurons (red) in the WT E14.5 brain, in different regions along the L-M axis as indicated. The neurogenic gradient (blue line) is reflected in high Ki67+ NPs in MP versus high Tuj1+ neurons in LP. (**F**) Graph showing the percentage of Pax6 RGs (red) and Tbr2+ IPs (green) at E14.5, in different regions along the L-M axis and in different genotypes as indicated. The number of Tbr2+ IPs is significantly decreased in E14.5 *KO* LPs. (**G-K**) Tbr2 (blue), Sox2 (red) and Ki67 (green) triple IF of *WT* (G,I-I'') and *KO* (H,J-J'') neocortices at E17.5, in different L-M regions as indicated, showing abundant Ki67+Sox2+Tbr2- bRGs in mutant brains. Numbers of bRGs are quantified in (K). (**L-N''**) BrdU (green) and EdU (red) IF of *WT* (L-M'') and *KO* (N-N'') E14.5 LP (M,N), DP (M',N') and MP (M'',N''). (**O**) Cell cycle duration along L-M axis of E14.5 *WT* (blue columns) and *KO* (orange line) LP, DP and MP. Cell cycle in mutant NPs is accelerated only in LP and DP, consistent with Nr2f1 gradient expression. (**P**) Schematic representation of cellular and morphological consequences of Nr2f1-controlled neurogenesis. Distinct levels of Nr2f1 (red color code) can be appreciated along the latero-medial (L-M) axis of the mouse developing neocortex. High Nr2f1 (left) in lateral regions triggers early Tbr2 expression and NP cell cycle exit, causing early NP pool depletion. Low Nr2f1 levels in medial neocortex allow for a longer NP pool expansion and are associated with long-lasting neurogenic activity late in development, as shown for the hippocampal region (Flore et al., 2016; Parisot et al., 2017). Medium Nr2f1 levels are associated to high bRGs number and to the development of a gyrus-like morphology expanding tangentially. Globally, Nr2f1 modulation controls the physiology of NP population in a time- and region-specific manner, resulting in local morphological changes of the cortical surface. Nuclei (blue) were stained with DAPI. In (E,F,K,O) the number of positive cells was quantified in 100µm- or 200µm-width boxes, randomly placed across the LP. In graphs, data are represented as means ± SEM. 2-way ANOVA (*P<0.05; ***P<0.001). Data are represented as means ± SEM. Scale bars: 50µm.

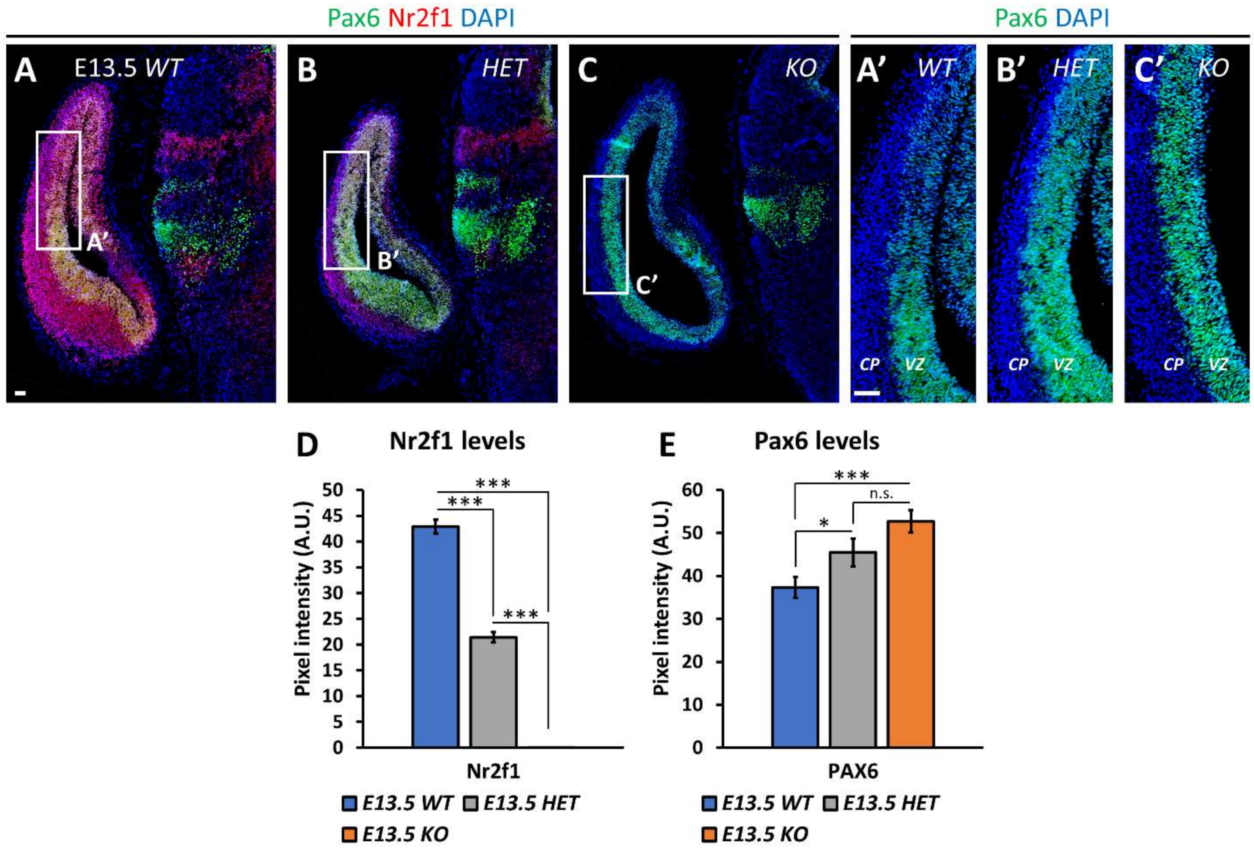

Appendix Figure S4. Nr2f1-mediated control of Pax6 level in neural progenitors. (**A-C'**) Pax6 (green in A-C') and Nr2f1 (red in A,B) IF of E13.5 *WT* (A,A'), *HET* (B,B') and *KO* (C,C') lateral pallia. (**D,E**) Nr2f1 (D) and Pax6 (E) pixel intensity quantification at E13.5 demonstrating increased Pax6 levels upon Nr2f1 removal, already in *HET* mutants. Nuclei (blue) were stained with DAPI. In (C,L) the pixel intensity was quantified in 100µm-width boxes, randomly placed across the lateral pallium, and data are represented as means ± SEM. 2-way ANOVA (Graphpad) (D,E; *P<0.05, ***P<0.001). Scale bars: 50µm. CP: cortical plate; VZ: ventricular zone.

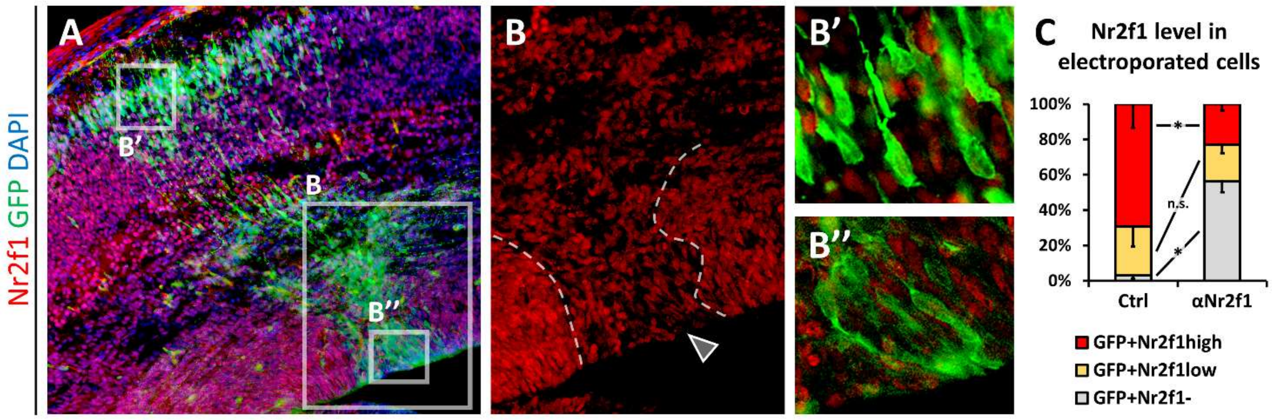

Appendix Figure S5. CRISPR/Cas9-mediated downregulation of NR2F1 expression in mouse cortical cells. (**A-C**) Nr2f1 (red) and GFP (green) immunostaining of a E14.5 mouse neocortex, 48-hours after electroporation of a CRISPR/Cas9 plasmid containing *gRNA* against *Nr2f1* start codon sequence (*PX458-αNr2f1).* Decreased Nr2f1 expression (arrowhead in B) is illustrated in neurons (B') and progenitors (B''). Quantification of high and low Nr2f1 expressing cells is shown in (C). Ctrl: control condition, i.e. electroporation of empty *PX458* vector. Data are represented as means ± SEM. 2-way ANOVA (C; *P<0.05).

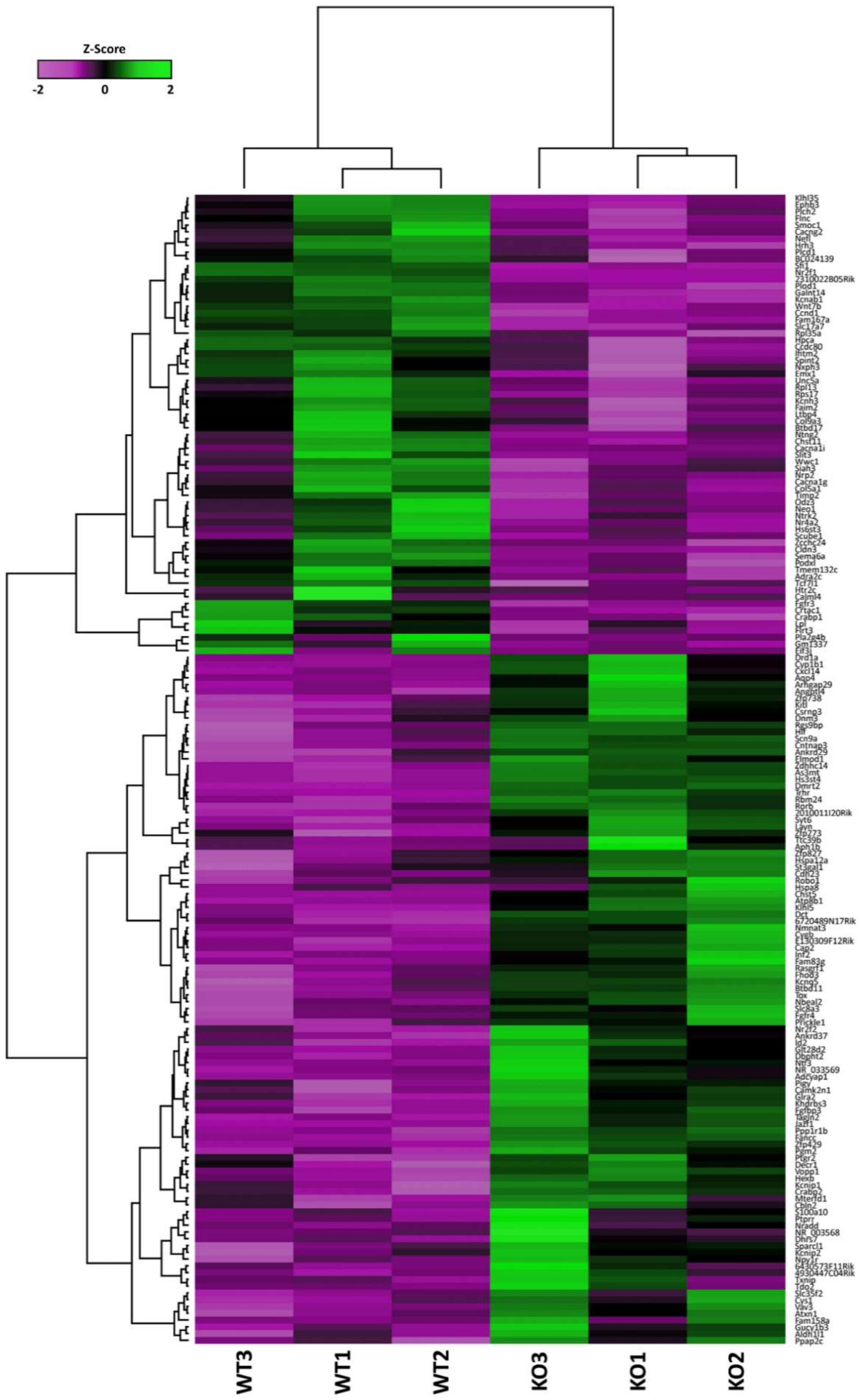

Appendix Figure S6. Hierarchical gene clustering analysis of E15.5 Nr2f1 WT and KO neocortices. Differentially expressed genes (DEGs) between WT and KO samples with a P-value <0.01 were considered for hierarchical gene clustering analysis (see Methods; RNA-Seq). Heat map color scale indicates normalized gene expression from high (green) to low (magenta) level. DEG genes are listed in Appendix Table S1.

SUPPLEMENTARY MATERIALS AND METHODS

Subjects, clinical phenotyping and DNA sequence analysis. The individuals and families with BBSOAS were recruited from multiple French clinical genetics centres. Informed consent was obtained from the legal guardians of the subjects described in this study according to the Declaration of Helsinki. Within the participating institutions, the phenotype was evaluated by clinical exam by the authors of this study. All patients underwent brain magnetic resonance imaging (MRI). MRI was re-evaluated by the neuroradiologist L.D'I. Protocol included 3D T1-w.i, axial and coronal FLAIR and T2-w.i. On volumetric T1-w.i. the following features were evaluated: morphology of the cerebral convolutions, cortical thickness, interface grey-white matter. FLAIR and T2-w.i. were evaluated for signal intensity of the cortex and of the white matter. Corpus callosum, optic nerves and chiasm were evaluated both on T1- and T2-w.i.

Exome sequencing (ES), bioinformatics analyses, interpretation, validation and subsequent analyses were carried out according to standard procedures. Clinical Research Exome kits (Agilent technologies, Santa Clara, CA) were used according to suppliers' protocols on a HiSeq 4000 (Illumina, San Diego, CA) platform. Variants were filtered and interpreted according to their potential pathogenicity, using various software programs and web resources as previously described (Orphanomix Physicians' Group et al., 2018). For patient 3, exome sequencing on Nextseq500 (Illumina, San Diego, CA) was performed following a previously published pipeline (Thevenon et al., 2016). Mutation and segregation studies were confirmed by Sanger sequencing, concordance of the trio by microsatellite analysis.

Animal procedures. All mouse experiments were conducted in accordance with relevant national and international guidelines and regulations (European Union rules; 2010/63/UE), and have been approved by the local ethical committee in France (CIEPAL NCE/2019-548). *Nr2f1* heterozygous (*HET*) and homozygous (*KO*) mice were generated and genotyped as previously described (Armentano et al., 2006). Littermates of *HET* and *KO* mice with normal *Nr2f1* alleles were used as control mice (herein called *WT*). Midday of the day of the vaginal plug was considered as embryonic day 0.5 (E0.5). Control and mutant mice were bred in a 129S2/SvPas background. Both male and female embryos and pups were used in this study; age is specified for each embryo/pup used in specific experiments. Standard housing conditions were approved by local ethical committee; briefly, adult mice were kept on a 12 hours light-dark cycle and housed three per cage with the recommended environmental enrichment (wooden cubes, cotton pad, igloo) with food and water *ad libidum*.

Cell culture (mouse neurospheres). For neurosphere assay, we followed previous protocols (Brewer and Torricelli, 2007; Reynolds et al., n.d.). Embryonic neocortical tissue was isolated at E12.5 or E15.5 and dissociated by mechanical trituration coupled to trypsin digestion. After centrifugation, cell pellet was resuspended in culture medium, which consisted of DMEM/F12 (Invitrogen) supplemented with Glutamine (2mM), Na-Piruvate (1mM), Penycillin-Streptomycin (100 U/ml), b-Mercaptoethanol (0.05mM), NEAA (1 mM), Heparin (2mg/ml) and N2/B27 (no vitamin A; Invitrogen). Cells were seeded at a density of 50.000 cells / cm^2 and incubated at 37°C, 5% CO2, 95% air. Single cells proliferated to form spherical clusters (primary neurospheres), floating in suspension. To promote NP proliferation and self-renewal, EGF (10ng/ml) and FGF2 (10ng/ml) were added to the culture medium. Each "passage" (every 3 days) consisted in harvesting the neurospheres, dissociating them into single cells and re-plating in fresh medium. As an alternative to neurosphere culture, NPs were dissociated by trypsin digestion and seeded as a monolayer on a Matrigel-coated plastic surface, in the same medium as described above (Conti et al., 2005).

Cell culture (human brain organoids). Human induced pluripotent (iPS) cells and brain organoids were obtained and cultured as previously described (Klaus et al., 2019; Lancaster et al., 2013). Briefly, iPS cells were seeded in low-bind plates to obtain embryoid bodies, which were then cultured in neural induction media, at 37 °C, 5% CO and ambient oxygen level with medium changes every 3 or 4 days. On day11 of the protocol, embryoid bodies were transferred in Matrigel droplets and subsequently grown in differentiation media on an orbital shaker, until the desired differentiation day (Day30, Day40 and Day70), to obtain $PAX6^{+}SOX2^{+}$ neural progenitors (NPs) and $DCX^{+}TUJ1^{+}$ neurons (Lancaster et al., 2013; Lancaster and Knoblich, 2014).

Immunofluorescence. Mouse embryonic brains/whole heads were dissected and fixed in 4% paraformaldehyde (PFA) at 4°C for 3 h in agitation, then washed in PBS 1X and dehydrated in 25% sucrose overnight at 4°C. P8 brains were fixed by intra-cardiac perfusion of 4% PFA, then processed as previously described (Armentano et al., 2007, 2006; Terrigno et al., 2018). Primary antibodies used: NR2F1 (Abcam ab181137, 1:1000, rabbit; R&D H8132, 1:1000, mouse), Sox2 (R&D AB2018, 1:500, mouse), PH3 (Phospho-Histone-3; Millipore 06-570, 1:2000, rabbit); Caspase-3 (Cell signaling #9661, 1:2000, rabbit), Pax6 (Millipore AB2237, 1:500, rabbit), Ki67 (ThermoFisher PA5-16446, 1:1000), GFAP (Dako, 1:200, rabbit), BLBP (Abcam ab32423, 1:2000, rabbit), Map2 (Sigma M4403, 1:2000, mouse); RFP (Abcam ab124754, 1:2000, rabbit); BrdU (Sigma B8434, 1:1000, mouse); P21 (Abcam ab188224, 1:200, rabbit); P-Vim (Phospho-Vimentin; MBL D076-3S, 1:2000, mouse); γ-Tub (gamma-Tubulin; ThermoFisher PA5-34815, 1:1000); Hopx (Sigma HPA030180, 1:1000, rabbit); Tbr2 (Millipore AB15894, 1:500, chicken); Satb2 (Abcam ab51502, 1:1000, mouse); Tbr1 (Abcam ab31940, 1:1000,

rabbit); Ctip2 (Abcam ab18465, 1:1000, rat); GFP (Invitrogen A11122, 1:1000, rabbit; or Abcam ab13970, 1:1000, chicken) and Tuj1 (β-III TUBULIN, Covance MRB-435P, 1:1000, rabbit; or Sigma T8660, 1:1000, mouse). All antibodies except GFP and RFP ones required antigen retrieval (10 minutes at 95°C in pH=6 Citric acid solution); the protocol varied for BrdU detection, were antigen retrieval was obtained by treating with 2N HCl with 0.5% Triton X-100 for 30 minutes at 37°C. Alexa Fluor 488, 555, 594 and 647 anti-mouse, anti-rabbit, anti-chicken or anti-rat IgG conjugates (Thermo Fisher scientific, all 1:500) were used as secondary antibodies. Images were acquired at an Apotome Zeiss, using the AxioVision software.

EdU/BrdU injection and cell cycle time calculation. The nucleoside analogs EdU (5-ethynyl-2'-deoxyuridine) and BrdU (5-bromo-2'-deoxyuridine) (both 10mg/kg) were injected intraperitoneally in pregnant females carrying embryos at the desired age. For the calculation of cell cycle time, we followed a previously described protocol (Martynoga et al., 2005). Briefly, EdU was injected, followed by BrdU 1.5 hours later (T_i = 1.5 hrs), then the embryos were collected at 2 hours from the first injection. After tissue fixation, cryostat cutting and immunostaining, the number of cycling progenitors (P_{cells}; Ki67$^+$), progenitors in S-phase (S_{cells} fraction; EdU$^+$BrdU$^+$Ki67$^+$) and progenitors that left the S-phase (L_{cells}, Leaving fraction; Ki67$^+$EdU$^+$ but BrdU$^-$) were quantified in a box of 100μm, randomly placed in the LP (unless otherwise specified). As the fraction of cells in a given phase of the cell cycle is directly proportional to the length of that phase, it is possible to calculate the total cell cycle time (T_C) as well as the length of S-phase (Ts) of the proliferating pool with the following formulas: $T_S = T_i / (L_{cells} / S_{cells})$; $Tc = Ts / (S_{cells} / P_{cells})$. For cumulative EdU cell cycle quantification (Contestabile et al., 2009; Nowakowski et al., 1989; Takahashi et al., 1993), E13.5 embryos were labelled with multiple EdU injections (at 0, 2, 4 and 6 hours). As EdU stays in the bloodstream and continues to label S-phase cells for 2 hours (Martynoga et al., 2005), animals were sacrificed at specific time points positioned 2 hours after the last injection (EdU2h, EdU4h, EdU6h and EdU8h; see *Figure 4H*). Ki67$^+$ staining was used to include only actively cycling cells in the analysis. The percentage of EdU-labeled progenitors over the total number of Ki67$^+$ cycling cells was quantified and data points for each group were best fitted with a least-square line, using linear regression analysis (Microsoft Excel). The y-intercept and the x-values for y = 1 extrapolated from the equation $y = a * x + b$ were used to calculate the total cell cycle time (T_C) and the length of S-phase (T_S): $T_C = 1/a$; $T_S = b/a$. Additionally, by quantifying the proportion of cells in G2-phase by means of PH3 immunostaining (Contestabile et al., 2009), the length of G2-phase was calculated. Finally, G1 was calculated as $T_C - (T_S + T_{G2} + T_M)$. For cell differentiation assays, pregnant females were injected with EdU at the desired stage, then embryos/pups were collected 24-h later or at P0/P8. Ki67 immunostaining allowed to distinguish between cycling cells (Ki67$^+$EdU$^+$) and differentiated cells (Ki67$^-$EdU$^+$). In all protocols listed, EdU was detected using EdU click-it technology (Invitrogen, C10340), while

BrdU required acid antigen retrieval (30 minutes at 37°C in 2N HCl with 0.5% Triton X-100) followed by immunostaining (Sigma B8434, 1:1000, mouse).

***In utero* electroporation.** *In utero* electroporation was performed on E12.5 mouse brains, by trying to target the latero-dorsal regions of the telencephalic dorsal pallium. The electroporations were performed using a Tweezertrode electrode (diameter 7 mm; BTX) connected to a NEPA21 Type-II electroporator (NEPA GENE), as previously described (Alfano et al., 2014, 2011; Parisot et al., 2017; Terrigno et al., 2018). The following parameters were used: four 35 V pulses, P(on) 50 ms, P(off) 1 s and 5% decay rate. The Sox2p-GFP and Tis21p-RFP plasmids were a kind gift of Elisa Marti and Gwenvael Le Dréau (Saade et al., 2013). For Nr2f1 knock-out/knock-down, we used a Crispr/Cas9 vector (Ran et al., 2013) in normal *wild-type* background mice. The anti-Nr2f1 *sgRNA* probe for Crispr/Cas9 construct was selected and checked for specificity *in silico* with Crispor (*http://crispor.tefor.net/)* then cloned in the PX458 empty vector (Addgene; pSpCas9(BB)-2A-GFP), with the assistance of iBV molecular biology facility (Virginie Virolle). In all cases, the 1X DNA solution for IUE was injected in one of the two brain telencephalic vesicles prior to the application of electric current, and consisted of endo-free TE buffer, 1X Fast Green and the desired plasmid at a final concentration of 1mg/ml. As a technical control, empty *pCIG2-IRES-GFP* or PX458 plasmids were electroporated at the same concentration. After electroporation, brains were dissected, fixed, cut and stained as previously described.

Electroporation of organoids. For electroporation, human brain organoids were kept in neural differentiation medium without antibiotics for 2 hours before the experiment. The organoids were placed in an electroporation chamber (Harvard Apparatus, Holliston, MA, USA), and the plasmid DNA was injected at a concentration of 1 µg/µl at several positions (Klaus et al., 2019). The organoids were then subjected to five pulses at 80 V with a 50-ms duration in an interval of 500ms using an ECM830 electroporation device (Harvard Apparatus) (Cárdenas et al., 2018; Klaus et al., 2019).

Cell cytometry. Neocortices were dissected on ice and digested using Papain kit, following manufacturer’s instructions. Low-bind Eppendorf tubes were used to limit material loss. Dissociated single cells were washed twice with ice-cold PBS 1X, then fixed by slowly adding Ethanol while mixing on a vortex, and stored at -20°C. For cytometric analysis, cells were immunostained following the same immunofluorescence protocol used for cryostat sections, with the only difference that a brief centrifugation (5 minutes at 1000 rpm) was performed between each solution change to recover the cells at the bottom of Eppendorf tubes and discard the supernatant. Cells were analyzed with BD LSR

Fortessa and FACS DIVA Software (Becton Dickinson) on the basis of 10.000 total events (debris excluded).

Real time RT-PCR. Neocortices were dissected in ice-cold PBS1X and stored at -80°C. Total RNA was extracted with NucleoSpin RNA II columns (Macherey-Nagel). RNA quantity and RNA quality were assessed with Nanodrop and gel electrophoresis. For each sample, 500ng of total RNA were reverse-transcribed using random examers (SuperScript III Reverse Transcriptase, Invitrogen); RT-PCR was performed using GoTaq SYBR Green qPCR Mix (Promega) on LightCycler (Roche). cDNA was diluted so that each reaction contained 2ng. Amplification take-off values were evaluated using the built-in LightCycler relative quantification analysis function, and relative expression was calculated with the $2^{-\Delta\Delta Ct}$ method as described in (Bertacchi et al., 2015), normalizing to the housekeeping gene *GAPDH*. Standard errors were obtained from the error propagation formula. For each genotype/time point, at least 2 embryos/cell pellets were analyzed (biological replicates), while at least 3 reactions were assembled per sample/gene analyzed (technical replicates). Primer sequences are listed in *Appendix Table S6*.

RNA-Seq. Total RNA was extracted using TRIZOL reagent (Invitrogen) and its integrity was analysed by using the DNF-471 Standard Sensitivity RNA Analysis Kit on Fragment Analyzer instrument (Advanced Analytical Technology, Ankeny, IA, USA). RNA-seq libraries were prepared from total RNA using TruSeq RNA Sample Preparation v2 (Illumina, San Diego, CA, USA) according to the manufacturer's protocol and were sequenced on Illumina NextSeq 500 platform (Illumina). Sequencing reads were trimmed out of the low-quality bases with Fastx Toolkit and were mapped on mm9 genome assembly by using TopHat v2.0.6 (Johns Hopkins University, Baltimore, MD, USA) and Differential Expressed genes were called by using DESEQ2 software (Love et al., 2014) (see *Appendix Table S1* for complete list of DEG genes). Genes with a P-value < 0.01 were considered for downstream Gene Ontology (GO) analysis (see *Appendix Tables S2-4* for GO analysis with DAVID web software, Ingenuity pathway analysis or Panther Overrepresentation test, respectively). GO analysis showed in *Figure 5O* was performed by using DAVID web software (Huang et al., 2009). The RNA-Seq datasets produced in this study are available in the following database: Gene Expression Omnibus GSE146595 (https://www.ncbi.nlm.nih.gov/geo/query/acc.cgi? acc=GSE146595).

Collection and processing of human foetuses. Cryostat section of human samples were kindly provided by Cécile Allet and Paolo Giacobini (Lille, France). All experiments involving the use of human samples conformed to the principles set out in the WMA Declaration of Helsinki and the Department of

Health and Human Services Belmont Report. Tissues were made available in accordance with French bylaws (Good practice concerning the conservation, transformation and transportation of human tissue to be used therapeutically, published on December 29, 1998). Furthermore, the studies on human foetal tissue were approved by the French agency for biomedical research (Agence de la Biomédecine, Saint-Denis la Plaine, France, protocol n°: PFS16–002). Non-pathological human foetuses (11 and 14 gestational weeks, n = 2) were obtained from voluntarily terminated pregnancies after obtaining written informed consent from the parents (Gynaecology Department, Jeanne de Flandre Hospital, Lille, France). Foetuses were fixed by immersion in 4% PFA at 4 °C for 7 days. The tissues were then cryoprotected in 30% sucrose/PBS for 3 days, embedded in Tissue-Tek OCT compound (Sakura Finetek, USA), frozen in dry ice and stored at -80°C until sectioning. Frozen samples were cut serially at 20 µm using a Leica CM 3050S cryostat (Leica Biosystems Nussloch GmbH, Germany).

Statistical Analysis. All data were statistically analyzed and graphically represented using Microsoft Office Excel software and GraphPad Prism (version 7.00). Quantitative data are shown as the mean ± standard error (SEM). For cell percentage/number quantification after immunofluorescence (IF), measurements were performed on at least 9 sections coming from 3 to 5 different animals, unless otherwise stated. To minimize subjective bias, sample identity (e.g. genotypes) was randomized by associating an identification number to each sample before processing. Fixed embryos with damaged tissues were excluded from any further analysis/processing. Microscope images were processed with Photoshop or ImageJ software, by randomly overlapping fixed-width (100µm) rectangular boxes on the area of interest (e.g. the lateral pallium of the neocortex), then quantifying positive cells inside the boxes. When calculating percentages over the total cell number, the latter was quantified by counting $DAPI^+$ nuclei, unless otherwise specified. Data were compared by two-tailed Student's *t*-test (when comparing two data group) or by 2-way ANOVA (analysis of variance; for comparison of three or more groups) and statistical significance was set as follows: *= $P \leq 0.05$; **= $P \leq 0.01$; ***= $P \leq 0.001$. Sample size, statistical test used and detailed list of statistical results for each experiment are listed in *Appendix Table S5*.

SUPPLEMENTARY REFERENCES

Alfano, C., Magrinelli, E., Harb, K., Hevner, R.F., Studer, M., 2014. Postmitotic control of sensory area specification during neocortical development. Nat. Commun. 5. https://doi.org/10.1038/ncomms6632

Alfano, C., Viola, L., Heng, J.I.-T., Pirozzi, M., Clarkson, M., Flore, G., De Maio, A., Schedl, A., Guillemot, F., Studer, M., 2011. COUP-TFI promotes radial migration and proper morphology of callosal projection neurons by repressing Rnd2 expression. Development 138, 4685–4697. https://doi.org/10.1242/dev.068031

Armentano, M., Chou, S.-J., Srubek Tomassy, G., Leingärtner, A., O'Leary, D.D.M., Studer, M., 2007. COUP-TFI regulates the balance of cortical patterning between frontal/motor and sensory areas. Nat. Neurosci. 10, 1277–1286. https://doi.org/10.1038/nn1958

Armentano, M., Filosa, A., Andolfi, G., Studer, M., 2006. COUP-TFI is required for the formation of commissural projections in the forebrain by regulating axonal growth. Development 133, 4151–4162. https://doi.org/10.1242/dev.02600

Bertacchi, M., Lupo, G., Pandolfini, L., Casarosa, S., D'Onofrio, M., Pedersen, R.A., Harris, W.A., Cremisi, F., 2015. Activin/Nodal Signaling Supports Retinal Progenitor Specification in a Narrow Time Window during Pluripotent Stem Cell Neuralization. Stem Cell Rep. 5, 532–545. https://doi.org/10.1016/j.stemcr.2015.08.011

Brewer, G.J., Torricelli, J.R., 2007. Isolation and culture of adult neurons and neurospheres. Nat. Protoc. 2, 1490–1498. https://doi.org/10.1038/nprot.2007.207

Cárdenas, A., Villalba, A., de Juan Romero, C., Picó, E., Kyrousi, C., Tzika, A.C., Tessier-Lavigne, M., Ma, L., Drukker, M., Cappello, S., Borrell, V., 2018. Evolution of Cortical Neurogenesis in Amniotes Controlled by Robo Signaling Levels. Cell 174, 590-606.e21. https://doi.org/10.1016/j.cell.2018.06.007

Contestabile, A., Fila, T., Bartesaghi, R., Ciani, E., 2009. Cell Cycle Elongation Impairs Proliferation of Cerebellar Granule Cell Precursors in the Ts65Dn Mouse, an Animal Model for Down Syndrome. Brain Pathol. 19, 224–237. https://doi.org/10.1111/j.1750-3639.2008.00168.x

Conti, L., Pollard, S.M., Gorba, T., Reitano, E., Toselli, M., Biella, G., Sun, Y., Sanzone, S., Ying, Q.-L., Cattaneo, E., Smith, A., 2005. Niche-Independent Symmetrical Self-Renewal of a Mammalian Tissue Stem Cell. PLoS Biol. 3, e283. https://doi.org/10.1371/journal.pbio.0030283

Flore, G., Di Ruberto, G., Parisot, J., Sannino, S., Russo, F., Illingworth, E.A., Studer, M., De Leonibus, E., 2016. Gradient COUP-TFI Expression Is Required for Functional Organization of the Hippocampal Septo-Temporal Longitudinal Axis. Cereb. Cortex bhv336. https://doi.org/10.1093/cercor/bhv336

Huang, D.W., Sherman, B.T., Lempicki, R.A., 2009. Systematic and integrative analysis of large gene lists using DAVID bioinformatics resources. Nat. Protoc. 4, 44–57. https://doi.org/10.1038/nprot.2008.211

Klaus, J., Kanton, S., Kyrousi, C., Ayo-Martin, A.C., Di Giaimo, R., Riesenberg, S., O'Neill, A.C., Camp, J.G., Tocco, C., Santel, M., Rusha, E., Drukker, M., Schroeder, M., Götz, M., Robertson, S.P., Treutlein, B., Cappello, S., 2019. Altered neuronal migratory trajectories in human cerebral organoids derived from individuals with neuronal heterotopia. Nat. Med. 25, 561–568. https://doi.org/10.1038/s41591-019-0371-0

Lancaster, M.A., Knoblich, J.A., 2014. Generation of cerebral organoids from human pluripotent stem cells. Nat. Protoc. 9, 2329–2340. https://doi.org/10.1038/nprot.2014.158

Lancaster, M.A., Renner, M., Martin, C.-A., Wenzel, D., Bicknell, L.S., Hurles, M.E., Homfray, T., Penninger, J.M., Jackson, A.P., Knoblich, J.A., 2013. Cerebral organoids model human brain development and microcephaly. Nature 501, 373–379. https://doi.org/10.1038/nature12517

Love, M.I., Huber, W., Anders, S., 2014. Moderated estimation of fold change and dispersion for RNA-seq data with DESeq2. Genome Biol. 15. https://doi.org/10.1186/s13059-014-0550-8

Martynoga, B., Morrison, H., Price, D.J., Mason, J.O., 2005. Foxg1 is required for specification of ventral telencephalon and region-specific regulation of dorsal telencephalic precursor proliferation and apoptosis. Dev. Biol. 283, 113–127. https://doi.org/10.1016/j.ydbio.2005.04.005

Nowakowski, R.S., Lewin, S.B., Miller, M.W., 1989. Bromodeoxyuridine immunohistochemical determination of the lengths of the cell cycle and the DNA-synthetic phase for an anatomically defined population. J. Neurocytol. 18, 311–318. https://doi.org/10.1007/BF01190834

Orphanomix Physicians' Group, Nambot, S., Thevenon, J., Kuentz, P., Duffourd, Y., Tisserant, E., Bruel, A.-L., Mosca-Boidron, A.-L., Masurel-Paulet, A., Lehalle, D., Jean-Marçais, N., Lefebvre, M., Vabres, P., El Chehadeh-Djebbar, S., Philippe, C., Tran Mau-Them, F., St-Onge, J., Jouan, T., Chevarin, M., Poé, C., Carmignac, V., Vitobello, A., Callier, P., Rivière, J.-B., Faivre, L., Thauvin-Robinet, C., 2018. Clinical whole-exome sequencing for the diagnosis of rare disorders with congenital anomalies and/or intellectual disability: substantial interest of prospective annual reanalysis. Genet. Med. 20, 645–654. https://doi.org/10.1038/gim.2017.162

Parisot, J., Flore, G., Bertacchi, M., Studer, M., 2017. COUP-TFI mitotically regulates production and migration of dentate granule cells and modulates hippocampal Cxcr4 expression. Dev. Camb. Engl. 144, 2045–2058. https://doi.org/10.1242/dev.139949

Ran, F.A., Hsu, P.D., Wright, J., Agarwala, V., Scott, D.A., Zhang, F., 2013. Genome engineering using the CRISPR-Cas9 system. Nat. Protoc. 8, 2281–2308. https://doi.org/10.1038/nprot.2013.143

Reynolds, B.A., Fraser, D., Weiss, S., n.d. bFCF Regulates the Proliferative Fate of Unipotent (Neuronal) and Bipotent (NeuronallAstroglial) EGF-Generated CNS Progenitor Cells 16.

Saade, M., Gutiérrez-Vallejo, I., Le Dréau, G., Rabadán, M.A., Miguez, D.G., Buceta, J., Martí, E., 2013. Sonic Hedgehog Signaling Switches the Mode of Division in the Developing Nervous System. Cell Rep. 4, 492–503. https://doi.org/10.1016/j.celrep.2013.06.038

Takahashi, T., Nowakowski, R., Caviness, V., 1993. Cell cycle parameters and patterns of nuclear movement in the neocortical proliferative zone of the fetal mouse. J. Neurosci. 13, 820–833. https://doi.org/10.1523/JNEUROSCI.13-02-00820.1993

Terrigno, M., Bertacchi, M., Pandolfini, L., Baumgart, M., Calvello, M., Cellerino, A., Studer, M., Cremisi, F., 2018. The microRNA miR-21 Is a Mediator of FGF8 Action on Cortical COUP-TFI Translation. Stem Cell Rep. 11, 756–769. https://doi.org/10.1016/j.stemcr.2018.08.002

Thevenon, J., Duffourd, Y., Masurel-Paulet, A., Lefebvre, M., Feillet, F., El Chehadeh-Djebbar, S., St-Onge, J., Steinmetz, A., Huet, F., Chouchane, M., Darmency-Stamboul, V., Callier, P., Thauvin-Robinet, C., Faivre, L., Rivière, J.B., 2016. Diagnostic odyssey in severe neurodevelopmental disorders: toward clinical whole-exome sequencing as a first-line diagnostic test: Diagnostic odyssey in severe neurodevelopmental disorders. Clin. Genet. 89, 700–707. https://doi.org/10.1111/cge.12732

Appendix Table S1

Complete list of differentially expressed genes (RNA-Seq)

gene	baseMean	log2FoldChan	lfcSE	stat	pvalue	padj
Nr2f1	1499,983514	-4,078788491	0,164914389	-24,73276296	4,75E-135	7,84E-131
Trhr	179,7005052	1,633669115	0,210479728	7,761645872	8,38E-15	6,92E-11
Dct	586,3945767	1,176621527	0,170217375	6,912464276	4,76E-12	2,61E-08
Zfp429	24,09631309	4,791991507	0,6973177	6,872034806	6,33E-12	2,61E-08
Cxcl14	46,12166689	2,522234068	0,423677341	5,953195572	2,63E-09	8,61E-06
Syt6	744,3612113	1,263155484	0,213205004	5,924605252	3,13E-09	8,61E-06
Wnt7b	2097,895066	-0,828312875	0,146011174	-5,67294169	1,40E-08	3,31E-05
Dbpht2	126,6066704	1,529391764	0,270688504	5,650006332	1,60E-08	3,31E-05
Fgfr3	1403,276634	-0,895381589	0,159207442	-5,623993329	1,87E-08	3,42E-05
Ppp1r1b	409,8023754	0,93136866	0,172803696	5,389749651	7,06E-08	0,000115842
As3mt	120,3746795	1,269242882	0,23620276	5,373531125	7,72E-08	0,000115842
Inf2	483,5106635	1,064881224	0,203553964	5,231444298	1,68E-07	0,000213613
Atp8b1	64,17380937	1,68168653	0,321462122	5,231367594	1,68E-07	0,000213613
Rorb	539,8500769	0,90924999	0,174797708	5,201727203	1,97E-07	0,000232759
Tox	675,0691193	0,833957934	0,170944612	4,878527162	1,07E-06	0,001175975
Scn9a	140,776018	1,271705745	0,266850224	4,765616174	1,88E-06	0,001884288
Aqp4	29,3040376	2,221139636	0,466676887	4,759480701	1,94E-06	0,001884288
Hs3st4	1030,724724	0,681343922	0,144914573	4,70169361	2,58E-06	0,002365688
Ccnd1	498,8898251	-0,776709506	0,167609347	-4,634046475	3,59E-06	0,003114795
Rasgrf1	289,7192791	1,028585442	0,227970401	4,511925402	6,42E-06	0,005301232
Klhl35	109,8521649	-1,280369292	0,289228742	-4,426839755	9,56E-06	0,007383985
6720489N17Rik	121,9958666	1,100104997	0,249196181	4,414614192	1,01E-05	0,007383985
Nbeal2	142,7986311	1,115625839	0,252966379	4,410174353	1,03E-05	0,007383985
Kcnab1	62,25526028	-1,432700224	0,325483658	-4,401757783	1,07E-05	0,007383985
Zfp738	327,1117201	0,861887928	0,19697606	4,375597361	1,21E-05	0,007994562
Cap2	402,4319889	0,763967365	0,175344982	4,356938846	1,32E-05	0,008372232
Nefl	169,1110701	-1,337840114	0,308626073	-4,334825309	1,46E-05	0,008916801
Vav3	91,78728598	1,216416164	0,282051618	4,312743084	1,61E-05	0,009504032
Cygb	298,9975268	0,802422401	0,186561315	4,301118926	1,70E-05	0,00965748
Galnt14	69,50088546	-1,334459352	0,310779016	-4,293917168	1,76E-05	0,00965748
Drd1a	113,7959218	1,097695673	0,256348935	4,282037185	1,85E-05	0,009717167
Rbm24	416,4683463	0,723234905	0,16905111	4,278202632	1,88E-05	0,009717167
Flnc	112,4195468	-1,114020664	0,266388839	-4,181934447	2,89E-05	0,014455469
Kcnq5	298,3900185	0,925430493	0,225018064	4,112694234	3,91E-05	0,01898291
Fhod3	1276,852778	0,651245405	0,158955714	4,097024178	4,18E-05	0,019733843
S100a10	185,7876741	1,302125292	0,319602266	4,074205442	4,62E-05	0,021167164
Cldn3	60,16298548	-1,383569373	0,340653023	-4,061520901	4,88E-05	0,021746943
Adra2c	64,41427326	-1,308460666	0,323881592	-4,039935272	5,35E-05	0,023221108
Zdhhc14	117,5571477	0,943204746	0,236027333	3,99616745	6,44E-05	0,02724269
Hpca	343,1114382	-0,892661283	0,22385882	-3,987608274	6,67E-05	0,027538057
Col5a1	183,246478	-1,015718464	0,256135102	-3,965557459	7,32E-05	0,029475584
Slit3	76,86476126	-1,377619431	0,350068998	-3,935279727	8,31E-05	0,032654284
Ntf3	45,54970543	1,393855104	0,361570132	3,855006211	0,000115727	0,044417519
Cntnap3	76,59012774	1,075082247	0,279687716	3,843866522	0,000121111	0,045427596
Unc5a	436,7637817	-0,783178002	0,205075615	-3,818971863	0,000134009	0,048387792
Pla2g4b	283,4869355	-1,192312152	0,312336362	-3,817397837	0,000134867	0,048387792
Eif3j	32,08015426	-2,616540846	0,690200277	-3,790987821	0,000150049	0,052483226
Cdh23	37,90183814	1,751507049	0,462537874	3,786732173	0,000152641	0,052483226
Angptl4	42,9205622	1,387065366	0,367634114	3,772950644	0,000161328	0,053436104
Sfi1	690,7767739	-0,572537132	0,151782624	-3,772086142	0,000161888	0,053436104
Rps17	135,2932998	-0,954526495	0,259176471	-3,682921098	0,000230577	0,073442783
Crtac1	155,6392763	-0,818676131	0,222344738	-3,682012628	0,0002314	0,073442783
Nrp2	1040,858948	-0,706183589	0,19383504	-3,643219461	0,000269249	0,083843148
Ntng2	776,5815766	-0,724464702	0,199483845	-3,631696109	0,000281565	0,084934381
Jazf1	383,3542984	0,619298047	0,170589453	3,630341947	0,000283046	0,084934381

2010011I20Rik	1490,523368	0,511461638	0,142158208	3,59783403	0,000320878	0,094567408
Ifitm2	122,7200569	-0,999537398	0,278926205	-3,583519155	0,000338996	0,096917063
Odz3	1459,84348	-0,747354035	0,20862467	-3,582289843	0,000340596	0,096917063
Prickle1	344,1637487	0,716313378	0,200453839	3,57345802	0,000352298	0,09854783
Fam83g	13,48821144	2,3213919	0,654162493	3,548647201	0,000387216	0,106510077
4930447C04Rik	57,52580093	1,17911017	0,338269655	3,485710739	0,000490831	0,132798067
Nmnat3	9,18953538	2,814989202	0,810437215	3,473420456	0,00051387	0,136788787
Fam167a	249,7057189	-0,657389063	0,190308103	-3,454340897	0,00055164	0,144512063
Plch2	440,6843005	-0,664479273	0,19436942	-3,418641023	0,000629347	0,161134439
Ltbp4	376,9706085	-0,707862778	0,207208637	-3,416183742	0,000635054	0,161134439
Gm1337	10,79326177	-3,061974408	0,897923369	-3,410062054	0,000649481	0,161134439
Htr2c	30,85014541	-2,21369402	0,649537116	-3,408110121	0,000654145	0,161134439
Nr4a2	277,3796679	-0,756250724	0,223568529	-3,382634966	0,00071794	0,174248238
Hexb	297,7858567	0,623575301	0,185731833	3,357395937	0,000786804	0,188194286
Dmrt2	45,63098622	1,126484958	0,336848803	3,344185719	0,000825245	0,194569128
Khdrbs3	2175,164378	0,477421872	0,143176029	3,334509815	0,000854499	0,198628859
Cacna1g	619,1003175	-0,641335428	0,192749536	-3,327299465	0,000876921	0,201009734
Nr2f2	760,1189878	0,600965333	0,180849846	3,323007164	0,000890526	0,201332132
Cbln2	138,8240755	0,923503578	0,279997707	3,298254071	0,000972881	0,21697867
Tagln2	315,7256035	0,587728618	0,17844009	3,293702769	0,00098877	0,217582125
Hspa12a	2860,910119	0,529842401	0,161138702	3,288113863	0,00100861	0,219027707
Fgfr4	60,53165435	1,076446433	0,328527581	3,276578579	0,001050731	0,225211162
Npy1r	64,59714484	1,109003759	0,339057078	3,270846797	0,00107226	0,22687918
Cacng2	94,5066025	-0,962378331	0,295696001	-3,254620722	0,001135439	0,237206125
Zcchc24	227,7772313	-0,705049398	0,218325926	-3,229343438	0,001240748	0,255966304
Hspa8	1727,193261	0,715257348	0,221851607	3,224035008	0,001263979	0,257038226
Hlf	108,139081	0,831368651	0,258102589	3,221078305	0,001277092	0,257038226
Kitl	1533,649902	0,509584558	0,158878493	3,207385401	0,001339474	0,266345561
Adcyap1	74,40148026	0,933729566	0,291660767	3,201423272	0,001367505	0,268220249
Slc17a7	562,5423926	-0,523063652	0,163641306	-3,196403549	0,001391523	0,268220249
Chst11	354,8665945	-0,642826863	0,201249134	-3,194184498	0,001402265	0,268220249
Ankrd37	55,84827307	1,067184391	0,334352273	3,191796427	0,001413909	0,268220249
Kcnip1	441,0589558	0,613845336	0,192575508	3,187556617	0,001434804	0,269090902
Rpl35a	102,4972298	-0,855821604	0,268836546	-3,18342732	0,001455426	0,269891668
Gucy1b3	548,2013343	0,602992485	0,189911087	3,1751305	0,001497691	0,2746433
Crabp1	42,27867916	-1,199472538	0,378519225	-3,168855	0,001530407	0,27748023
Id2	4903,657733	0,478675119	0,151203891	3,165759259	0,001546788	0,27748023
Atxn1	452,3001305	0,560775427	0,178045805	3,149613249	0,001634867	0,290127419
Col9a3	230,6009885	-0,823868453	0,262024579	-3,144241109	0,001665182	0,292363407
Kcnh3	192,1896389	-0,752540227	0,240447282	-3,129751441	0,001749543	0,30394161
Pgm2	231,1637968	0,612269835	0,198111461	3,09053213	0,001997982	0,343486382
Btbd11	297,1268949	0,580272197	0,188270802	3,082114652	0,002055357	0,349707264
Spint2	86,43464333	-0,94856643	0,31009891	-3,058915716	0,002221396	0,374101283
Ntrk2	779,4645281	-0,682859101	0,223606152	-3,053847565	0,002259268	0,376636028
Fgfbp3	406,5906017	0,532113365	0,174920471	3,042030257	0,002349883	0,387319733
Slc35f2	117,0694701	0,816332797	0,268581208	3,039426334	0,002370292	0,387319733
Plcd1	98,05403223	-0,893071559	0,294128049	-3,03633592	0,002394724	0,387475743
6430573F11Rik	83,62369079	0,946728576	0,312760719	3,027006014	0,00246989	0,39575798
Fancc	101,722201	0,735420643	0,243945309	3,014694756	0,00257238	0,408216917
Mterfd1	476,9600702	0,614650481	0,204961779	2,998854154	0,00270997	0,425955661
Rpl13	97,9589241	-0,869269311	0,291540337	-2,981643365	0,002867058	0,445651237
Camk2n1	792,6480995	0,507961131	0,170542146	2,978507911	0,002896556	0,445651237
2310022B05Rik	1360,101793	-0,417000414	0,140428808	-2,969479133	0,002983051	0,445651237
Calml4	12,12650388	-2,793689996	0,941448826	-2,967436912	0,003002939	0,445651237
Cyp1b1	23,57730356	1,370419631	0,461864057	2,96714934	0,003005749	0,445651237
Lpl	916,1992373	-0,61087227	0,205969555	-2,965837688	0,003018598	0,445651237
Timp2	981,2124407	-0,485520367	0,163736307	-2,965257837	0,003024293	0,445651237
Elmod1	1420,396493	0,4526745	0,154064123	2,938221375	0,003301012	0,48212306
Neo1	3675,760218	-0,503899212	0,17183615	-2,932440066	0,003363098	0,485747115
Decr1	170,79166	0,72765365	0,248307626	2,930452288	0,00338469	0,485747115
Ptprr	67,63708662	0,972778102	0,333283849	2,918767605	0,003514181	0,499983133

E130309F12Rik	568,0437805	0,466689595	0,160899278	2,900507706	0,003725587	0,525530633
Flrt3	962,5216644	-0,471740107	0,163854035	-2,879026488	0,003989048	0,557925873
Scube1	985,1025264	-0,642155419	0,223469316	-2,873573121	0,004058571	0,562879483
Cacna1i	106,7902168	-0,865932729	0,30213131	-2,866080739	0,004155882	0,571572357
St3gal1	1261,844424	0,60660966	0,212099376	2,860025677	0,004236067	0,577785546
NR_033540	4,87382392	-4,165556036	1,457838185	-2,857351438	0,004271926	0,57790049
BC024139	161,3695239	-0,753588673	0,265024803	-2,843464708	0,004462595	0,598785936
Zfp273	132,8227252	0,74370188	0,26200356	2,838518228	0,004532353	0,603241572
Plod1	133,6670587	-0,651018876	0,230084457	-2,829477859	0,004662403	0,61558634
Klhl5	1910,370845	0,397827	0,140878601	2,823899419	0,004744327	0,618308798
Csrnp3	2183,37584	0,465038982	0,164733348	2,822980224	0,004757951	0,618308798
Glt28d2	48,1865832	0,942620144	0,337027431	2,796864759	0,005160113	0,659808025
Crabp2	39,94064421	1,067048279	0,381986088	2,793421832	0,005215363	0,659808025
Txnip	801,5366753	0,646900796	0,231649929	2,792579293	0,005228965	0,659808025
Ccdc80	414,5798069	-0,499931183	0,179053972	-2,792069771	0,005237206	0,659808025
Dhrs7	29,79491785	1,282170677	0,460311373	2,785442097	0,005345479	0,668346811
Fam158a	35,15644668	1,214045828	0,436508061	2,78126783	0,005414705	0,671911905
Sema6a	454,3911781	-0,489609054	0,176204946	-2,778633999	0,005458799	0,672328536
Chst5	26,39193018	1,206241974	0,435271643	2,77123951	0,005584333	0,681635572
Aqp1	28,72532117	-1,928395214	0,696337025	-2,769341777	0,005616968	0,681635572
Faim2	107,9045838	-0,747493111	0,270531297	-2,763055954	0,005726295	0,689830446
Aph1b	609,9459184	0,60183518	0,218541003	2,753877631	0,005889378	0,704335512
Fbp1	2,830989701	-4,498188136	1,635449862	-2,750428637	0,005951735	0,706672235
Ptgr2	288,4392745	0,549300049	0,200182541	2,743995794	0,006069632	0,713866275
Hs6st3	16,54143774	-1,861835146	0,678902302	-2,742419844	0,006098833	0,713866275
Pigy	181,4436882	0,628370535	0,229485443	2,738171656	0,006178182	0,718061368
Aldh1l1	210,955037	0,605666941	0,222021767	2,727961993	0,006372695	0,733816384
Tdo2	12,00190042	1,878922454	0,689155226	2,726413997	0,006402664	0,733816384
NR_003568	57,15104657	1,05265758	0,387462289	2,716800088	0,006591641	0,748144842
Tcf7l1	97,87717668	-0,738368404	0,272029042	-2,714299911	0,006641601	0,748144842
Arhgap29	462,0359599	0,459422722	0,169334403	2,713109175	0,006665514	0,748144842
Nxph3	427,250366	-0,553686882	0,204240652	-2,710953367	0,006709006	0,748144842
Ankrd29	32,56797426	1,124867873	0,416696683	2,699488428	0,006944617	0,769221212
Kcnip2	70,5174612	0,928121478	0,345664808	2,685033175	0,007252261	0,792688667
Nradd	176,5731782	0,715074211	0,266319871	2,685020113	0,007252544	0,792688667
Cys1	27,99241469	1,193192504	0,445479936	2,678442747	0,007396538	0,803108266
Wwc1	1186,39327	-0,462440382	0,173405731	-2,666811407	0,007657463	0,822833554
Ephb3	173,0076312	-0,579581762	0,217480504	-2,664982609	0,00769923	0,822833554
Btbd17	192,6575697	-0,603352029	0,226631902	-2,662255507	0,007761893	0,822833554
Hrh3	28,91272085	-1,219514892	0,458193174	-2,661573677	0,007777632	0,822833554
Slc8a3	329,4987759	0,513429126	0,193122593	2,658565826	0,007847402	0,824926887
Ppap2c	79,25035329	0,815904327	0,307435743	2,653901977	0,007956694	0,83112198
Emx1	709,2804204	-0,446008533	0,168471767	-2,647378497	0,00811185	0,836774866
Glra2	914,5027834	0,439015559	0,165831245	2,647363346	0,008112214	0,836774866
Layn	12,77279548	1,640680458	0,621286489	2,640779232	0,00827156	0,847911957
Nr0b1	8,131415337	-2,553758403	0,968698164	-2,636278768	0,008382084	0,852081392
Sparcl1	1251,608623	0,426735483	0,161953321	2,634928887	0,008415491	0,852081392
Siah3	82,24389578	-0,917695097	0,348675679	-2,631944676	0,008489769	0,854360619
Smoc1	286,943695	-0,528177534	0,201987043	-2,614907995	0,008925149	0,88369246
Dnm3	813,0660853	0,445613503	0,170442124	2,614456409	0,008936956	0,88369246
Zfp827	1021,953507	0,431616465	0,165152423	2,613443126	0,0089635	0,88369246
Rgs9bp	44,72729286	0,921770516	0,352867513	2,612228335	0,008995415	0,88369246
Ttc39b	842,2714772	0,56012472	0,214845867	2,60710028	0,009131261	0,891729808
Tmem132c	157,2196476	-0,611666101	0,235246013	-2,600112512	0,00931932	0,904741514
Robo1	2533,232096	0,458596145	0,176920399	2,592104394	0,009539082	0,92066088
Ttc21a	5,326130458	-3,657478773	1,412165641	-2,589978589	0,00959819	0,920979828
Plekhf2	528,6892823	0,540038071	0,208872329	2,585493605	0,009723968	0,927655273

Appendix Table S2

Gene Ontology (GO) analysis by DAVID software

Category	Term	Count	%	PValue	Genes	List Total	Pop Hits	Pop Total	Fold Enrichment	Bonferroni	Benjamini	FDR
GOTERM_BP_FAT	GO:0030182~neuron differentiation	16	9,58083832	1,65E-06	FGFR3, NTF3, EMX1, PTPRR, NR4A2, NTNG2, RORB, EPHB3, SLIT3, SEMA6A, RASGRF1, ROBO1, NTRK2, DRD1A, NEFL, CDH23	118	399	13588	4,617645809	0,00170093	0,00170093	0,00261231
GOTERM_BP_FAT	GO:0048666~neuron development	13	7,78443114	7,94E-06	SEMA6A, NTF3, ROBO1, RASGRF1, NTRK2, NR4A2, NTNG2, RORB, EPHB3, DRD1A, NEFL, SLIT3, CDH23	118	292	13588	5,126654284	0,00817978	0,00409829	0,01260294
KEGG_PATHWAY	mmu04010:MAPK signaling pathway	12	7,18562874	6,42E-05	FGFR4, FGFR3, NTF3, RASGRF1, NTRK2, CACNA1I, PTPRR, CACNA1G, CACNG2, FLNC, PLA2G4B, HSPA8	60	265	5738	4,330566038	0,00588673	0,00588673	0,07008578
GOTERM_BP_FAT	GO:0006928~cell motion	13	7,78443114	7,54E-05	NRP2, VAV3, NTF3, PODXL, NR4A2, EPHB3, KITL, SLIT3, SEMA6A, ROBO1, NR2F2, DRD1A, NR2F1	118	367	13588	4,078972891	0,07499322	0,02565004	0,11955151
GOTERM_BP_FAT	GO:0048667~cell morphogenesis involved in neuron differentiation	9	5,38922156	1,75E-04	SEMA6A, NTF3, ROBO1, NR4A2, NTNG2, EPHB3, NEFL, SLIT3, CDH23	118	182	13588	5,694356491	0,16538471	0,04418997	0,27703453
GOTERM_MF_FAT	GO:0022832~voltage-gated channel activity	9	5,38922156	1,92E-04	KCNQ5, KCNAB1, CACNA1I, CACNA1G, SCN9A, CACNG2, KCNIP2, KCNH3, KCNIP1	119	179	13288	5,614384301	0,05480877	0,05480877	0,25582734
GOTERM_MF_FAT	GO:0005244~voltage-gated ion channel activity	9	5,38922156	1,92E-04	KCNQ5, KCNAB1, CACNA1I, CACNA1G, SCN9A, CACNG2, KCNIP2, KCNH3, KCNIP1	119	179	13288	5,614384301	0,05480877	0,05480877	0,25582734
GOTERM_MF_FAT	GO:0005509~calcium ion binding	19	11,3772455	4,24E-04	SPARCL1, CRTAC1, LTBP4, SCUBE1, S100A10, SYT6, CACNG2, KCNIP2, KCNIP1, SLIT3, CALML4, SMOC1, PLCH2, HPCA, CACNA1G, PLCD1, PLA2G4B, CDH23, GALNT14	119	840	13288	2,525730292	0,11679004	0,06020749	0,56278198
GOTERM_BP_FAT	GO:0030030~cell projection organization	11	6,58682635	4,28E-04	SEMA6A, VAV3, NTF3, ROBO1, RASGRF1, NR4A2, NTNG2, EPHB3, NEFL, SLIT3, CDH23	118	319	13588	3,970777323	0,35776092	0,08475083	0,67717818
GOTERM_MF_FAT	GO:0019838~growth factor binding	6	3,59281437	4,42E-04	FGFBP3, FGFR3, NTF3, LTBP4, NTRK2, COL5A1	119	72	13288	9,305322129	0,1213897	0,04222074	0,58637386
GOTERM_BP_FAT	GO:0000904~cell morphogenesis involved in differentiation	9	5,38922156	4,90E-04	SEMA6A, NTF3, ROBO1, NR4A2, NTNG2, EPHB3, NEFL, SLIT3, CDH23	118	212	13588	4,888551327	0,39727848	0,08092113	0,77392197
GOTERM_BP_FAT	GO:0007409~axonogenesis	8	4,79041916	5,16E-04	SEMA6A, NTF3, ROBO1, NR4A2, NTNG2, EPHB3, NEFL, SLIT3	118	163	13588	5,651658521	0,41374119	0,07344776	0,81608127
GOTERM_BP_FAT	GO:0042391~regulation of membrane potential	7	4,19161677	5,64E-04	ATXN1, NTF3, HEXB, CACNA1I, CACNA1G, CACNG2, DRD1A	118	119	13588	6,773678963	0,44192748	0,07031392	0,89104586
GOTERM_BP_FAT	GO:0031175~neuron projection development	9	5,38922156	5,89E-04	SEMA6A, NTF3, ROBO1, RASGRF1, NR4A2, NTNG2, EPHB3, NEFL, SLIT3	118	218	13588	4,754004043	0,45601647	0,06541106	0,92992647
GOTERM_BP_FAT	GO:0007610~behavior	12	7,18562874	7,36E-04	NRP2, ATXN1, PPP1R1B, ROBO1, NTRK2, HEXB, NR4A2, SCN9A, NPY1R, HTR2C, DRD1A, CDH23	118	405	13588	3,411927181	0,5328578	0,07328774	1,16116927
GOTERM_BP_FAT	GO:0048812~neuron projection morphogenesis	8	4,79041916	8,14E-04	SEMA6A, NTF3, ROBO1, NR4A2, NTNG2, EPHB3, NEFL, SLIT3	118	176	13588	5,234206471	0,5692034	0,07369923	1,283943
GOTERM_MF_FAT	GO:0022836~gated channel activity	10	5,98802395	8,91E-04	KCNQ5, KCNAB1, GLRA2, CACNA1I, CACNA1G, SCN9A, CACNG2, KCNIP2, KCNH3, KCNIP1	119	281	13288	3,973803044	0,22976381	0,06318038	1,17932617
GOTERM_MF_FAT	GO:0022843~voltage-gated cation channel activity	7	4,19161677	9,71E-04	KCNQ5, KCNAB1, CACNA1I, CACNA1G, SCN9A, CACNG2, KCNH3	119	128	13288	6,106617647	0,24767032	0,0553267	1,28490354
GOTERM_CC_FAT	GO:0005578~proteinaceous extracellular matrix	10	5,98802395	0,001215	WNT7B, CRTAC1, SPARCL1, LTBP4, SMOC1, CCDC80, NTNG2, TIMP2, COL5A1, ANGPTL4	111	297	12504	3,792883793	0,1887142	0,1887142	1,47531596
GOTERM_CC_FAT	GO:0005886~plasma membrane	41	24,5508982	0,001258	NRP2, SLC8A3, FGFR3, CLDN3, TRHR, GLRA2, AQP4, SYT6, NEO1, EPHB3, KCNIP2, KCNIP1, KCNQ5, HRH3, ROBO1, UNC5A, PLCH2, SCN9A, ADRA2C, ODZ3, CDH23, LPL, VAV3, CAP2, PIGY, NTF3, SCUBE1, PODXL, PTPRR, NTNG2, NPY1R, KITL, CAMK2N1, SLC17A7, LAYN, NTRK2, CACNA1G, DRD1A, PLA2G4B, HTR2C, FAIM2	111	2906	12504	1,589330556	0,1946947	0,10261196	1,52711033
GOTERM_MF_FAT	GO:0022838~substrate specific channel activity	11	6,58682635	0,001369	KCNQ5, KCNAB1, GLRA2, CACNA1I, CACNA1G, SCN9A, AQP4, CACNG2, KCNIP2, KCNH3, KCNIP1	119	360	13288	3,411951447	0,33068316	0,06472651	1,80799626
GOTERM_BP_FAT	GO:0042069~regulation of catecholamine metabolic process	3	1,79640719	0,001501	NR4A2, HTR2C, DRD1A	118	7	13588	49,35108959	0,7883779	0,12138877	2,35488189
GOTERM_BP_FAT	GO:0042053~regulation of dopamine metabolic process	3	1,79640719	0,001501	NR4A2, HTR2C, DRD1A	118	7	13588	49,35108959	0,7883779	0,12138877	2,35488189
GOTERM_BP_FAT	GO:0033238~regulation of cellular amine metabolic process	3	1,79640719	0,001501	NR4A2, HTR2C, DRD1A	118	7	13588	49,35108959	0,7883779	0,12138877	2,35488189
GOTERM_MF_FAT	GO:0022803~passive transmembrane transporter activity	11	6,58682635	0,001517	KCNQ5, KCNAB1, GLRA2, CACNA1I, CACNA1G, SCN9A, AQP4, CACNG2, KCNIP2, KCNH3, KCNIP1	119	365	13288	3,365212386	0,3591469	0,06158688	2,00171887
GOTERM_MF_FAT	GO:0015267~channel activity	11	6,58682635	0,001517	KCNQ5, KCNAB1, GLRA2, CACNA1I, CACNA1G, SCN9A, AQP4, CACNG2, KCNIP2, KCNH3, KCNIP1	119	365	13288	3,365212386	0,3591469	0,06158688	2,00171887
GOTERM_CC_FAT	GO:0031012~extracellular matrix	10	5,98802395	0,001598	WNT7B, CRTAC1, SPARCL1, LTBP4, SMOC1, CCDC80, NTNG2, TIMP2, COL5A1, ANGPTL4	111	309	12504	3,645587335	0,24054088	0,08763601	1,93646795
GOTERM_MF_FAT	GO:0005261~cation channel activity	9	5,38922156	0,001647	KCNQ5, KCNAB1, CACNA1I, CACNA1G, SCN9A, CACNG2, KCNIP2, KCNH3, KCNIP1	119	248	13288	4,052317701	0,38304094	0,05858299	2,17079024
GOTERM_BP_FAT	GO:0048858~cell projection morphogenesis	8	4,79041916	0,001807	SEMA6A, NTF3, ROBO1, NR4A2, NTNG2, EPHB3, NEFL, SLIT3	118	202	13588	4,560496728	0,84593295	0,1340035	2,82933681
GOTERM_BP_FAT	GO:0032990~cell part morphogenesis	8	4,79041916	0,002375	SEMA6A, NTF3, ROBO1, NR4A2, NTNG2, EPHB3, NEFL, SLIT3	118	212	13588	4,345378957	0,9144228	0,16104269	3,70212148
GOTERM_BP_FAT	GO:0019748~secondary metabolic process	5	2,99401198	0,002934	NMNAT3, DCT, CYP1B1, CRABP2, AS3MT	118	69	13588	8,344387128	0,95208393	0,18335731	4,55534895
GOTERM_BP_FAT	GO:0042490~mechanoreceptor differentiation	4	2,39520958	0,003612	FGFR3, NTF3, NTRK2, CDH23	118	36	13588	12,79472693	0,9762781	0,20850742	5,57952283
GOTERM_BP_FAT	GO:0007617~mating behavior	3	1,79640719	0,003843	PPP1R1B, HEXB, DRD1A	118	11	13588	31,40523883	0,98133828	0,2087909	5,92651268
GOTERM_MF_FAT	GO:0005216~ion channel activity	10	5,98802395	0,003895	KCNQ5, KCNAB1, GLRA2, CACNA1I, CACNA1G, SCN9A, CACNG2, KCNIP2, KCNH3, KCNIP1	119	349	13288	3,199537695	0,68129092	0,11931296	5,06363935
GOTERM_BP_FAT	GO:0045597~positive regulation of cell differentiation	7	4,19161677	0,004033	LPL, WNT7B, FGFR3, ID2, NTF3, KITL, NEFL	118	175	13588	4,606101695	0,98467672	0,20715881	6,21061529
GOTERM_MF_FAT	GO:0046873~metal ion transmembrane transporter activity	9	5,38922156	0,004308	KCNQ5, KCNAB1, CACNA1I, CACNA1G, SCN9A, CACNG2, KCNIP2, KCNH3, KCNIP1	119	290	13288	3,46543031	0,71776162	0,11882614	5,58648798
GOTERM_BP_FAT	GO:0007626~locomotory behavior	8	4,79041916	0,004601	NRP2, ATXN1, ROBO1, HEXB, NR4A2, NPY1R, DRD1A, CDH23	118	239	13588	3,854478406	0,99150589	0,22195233	7,05592633
GOTERM_BP_FAT	GO:0016477~cell migration	8	4,79041916	0,004706	NRP2, VAV3, PODXL, NR4A2, NR2F2, DRD1A, KITL, NR2F1	118	240	13588	3,838418079	0,99238514	0,21642044	7,21164432
GOTERM_BP_FAT	GO:0030001~metal ion transport	11	6,58682635	0,004814	SLC8A3, SLC17A7, KCNQ5, KCNAB1, CACNA1I, CACNA1G, SCN9A, CACNG2, KCNIP2, KCNH3, KCNIP1	118	442	13588	2,865787254	0,99319579	0,21150502	7,37177681
GOTERM_BP_FAT	GO:0000902~cell morphogenesis	9	5,38922156	0,005233	SEMA6A, NTF3, ROBO1, NR4A2, NTNG2, EPHB3, NEFL, SLIT3, CDH23	118	309	13588	3,353957545	0,99559337	0,21852706	7,9872259
GOTERM_CC_FAT	GO:0043005~neuron projection	8	4,79041916	0,00586	NRP2, SEMA6A, ROBO1, RASGRF1, NTRK2, CYGB, NEFL, CAMK2N1	111	245	12504	3,678323221	0,63611101	0,22331981	6,93243138
PANTHER_PATHWAY	P00008:Axon guidance mediated by Slit/Robo	4	2,39520958	0,006195	ROBO1, NTNG2, NEO1, SLIT3	34	34	2921	10,10726644	0,20046426	0,20046426	5,39211585
GOTERM_MF_FAT	GO:0043167~ion binding	49	29,3413174	0,00647	ZCCHC24, KCNAB1, LTBP4, ZFP738, GLRA2, HEXB, RORB, SYT6, KCNIP2, KCNIP1, KCNQ5, TDO2, PLOD1, ATP8B1, CYGB, ZFP273, NR2F2, CDH23, NR2F1, SPARCL1, CRTAC1, SCUBE1, ZFP429, CACNG2, ARHGAP29, SLIT3, PGM2, ZFP827, ZDHHC14, GUCY1B3, KCNH3, CYP1B1, DCT, CALML4, PLCH2, SMOC1, SCN9A, PLCD1, GALNT14, VAV3, PTGR2, NR4A2, S100A10, SLC17A7, PRICKLE1, HPCA, CACNA1G, JAZF1, PLA2G4B	119	3934	13288	1,390831066	0,85071203	0,1587766	8,27981547
GOTERM_BP_FAT	GO:0009791~post-embryonic development	5	2,99401198	0,006713	EMX1, CHST11, NR4A2, SCN9A, CDH23	118	87	13588	6,617962205	0,9990554	0,26126426	10,1362965
GOTERM_BP_FAT	GO:0043085~positive regulation of catalytic activity	8	4,79041916	0,007363	VAV3, RASGRF1, APH1B, NR4A2, HTR2C, DRD1A, KITL, ADCYAP1	118	261	13588	3,529579843	0,99951983	0,27268112	11,0645201
GOTERM_BP_FAT	GO:0010035~response to inorganic substance	5	2,99401198	0,008156	CCND1, AS3MT, CACNA1G, NR4A2, FANCC	118	92	13588	6,258290346	0,99978984	0,2873077	12,1850475
GOTERM_BP_FAT	GO:0030534~adult behavior	5	2,99401198	0,008467	ATXN1, NR4A2, HTR2C, DRD1A, CDH23	118	93	13588	6,190996902	0,99984805	0,28691277	12,6209738
GOTERM_MF_FAT	GO:0043169~cation binding	48	28,742515	0,008564	ZCCHC24, CYP1B1, KCNAB1, ZFP738, LTBP4, HEXB, SYT6, RORB, KCNIP2, KCNIP1, DCT, KCNQ5, TDO2, PLOD1, CALML4, SMOC1, PLCH2, SCN9A, ATP8B1, CYGB, PLCD1, ZFP273, NR2F2, CDH23, GALNT14, NR2F1, PTGR2, VAV3, CRTAC1, SPARCL1, SCUBE1, ZFP429, NR4A2, S100A10, ARHGAP29, CACNG2, SLIT3, SLC17A7, PGM2, ZFP827, ZDHHC14, PRICKLE1, HPCA, JAZF1, CACNA1G, GUCY1B3, KCNH3, PLA2G4B	119	3885	13288	1,379630771	0,91953661	0,18941257	10,8201533
GOTERM_MF_FAT	GO:0003707~steroid hormone receptor activity	4	2,39520958	0,009346	NR4A2, RORB, NR2F2, NR2F1	119	49	13288	9,115417596	0,93615888	0,19074637	11,7530381
GOTERM_BP_FAT	GO:0007631~feeding behavior	4	2,39520958	0,009596	NTRK2, NPY1R, HTR2C, DRD1A	118	51	13588	9,031571951	0,99995324	0,30876625	14,1870308
GOTERM_MF_FAT	GO:0031420~alkali metal ion binding	7	4,19161677	0,010124	SLC17A7, KCNQ5, KCNAB1, SCN9A, KCNIP2, KCNH3, KCNIP1	119	206	13288	3,794403198	0,94927809	0,19180908	12,6707465
GOTERM_BP_FAT	GO:0007411~axon guidance	5	2,99401198	0,010139	SEMA6A, NTF3, ROBO1, EPHB3, SLIT3	118	98	13588	5,875129713	0,99997346	0,31361262	14,9294948
GOTERM_BP_FAT	GO:0051094~positive regulation of developmental process	7	4,19161677	0,010474	LPL, WNT7B, FGFR3, ID2, NTF3, KITL, NEFL	118	214	13588	3,766671947	0,99998131	0,31300225	15,3858674
GOTERM_BP_FAT	GO:0050804~regulation of synaptic transmission	5	2,99401198	0,010863	NTF3, RASGRF1, NTRK2, HTR2C, DRD1A	118	100	13588	5,757627119	0,99998755	0,313713	15,9120072
GOTERM_BP_FAT	GO:0032989~cellular component morphogenesis	9	5,38922156	0,010907	SEMA6A, NTF3, ROBO1, NR4A2, NTNG2, EPHB3, NEFL, SLIT3, CDH23	118	351	13588	2,952629292	0,99998811	0,30635211	15,9709993
GOTERM_BP_FAT	GO:0048870~cell motility	8	4,79041916	0,011403	NRP2, VAV3, PODXL, NR4A2, NR2F2, DRD1A, KITL, NR2F1	118	284	13588	3,243733588	0,99999292	0,30966379	16,6374816
GOTERM_BP_FAT	GO:0051674~localization of cell	8	4,79041916	0,011403	NRP2, VAV3, PODXL, NR4A2, NR2F2, DRD1A, KITL, NR2F1	118	284	13588	3,243733588	0,99999292	0,30966379	16,6374816
GOTERM_BP_FAT	GO:0019098~reproductive behavior	3	1,79640719	0,011424	PPP1R1B, HEXB, DRD1A	118	19	13588	18,18198037	0,99999307	0,30232229	16,664962
GOTERM_BP_FAT	GO:0007618~mating	3	1,79640719	0,011424	PPP1R1B, HEXB, DRD1A	118	19	13588	18,18198037	0,99999307	0,30232229	16,664962
GOTERM_MF_FAT	GO:0008227~amine receptor activity	4	2,39520958	0,011584	HRH3, ADRA2C, HTR2C, DRD1A	119	53	13288	8,427461551	0,9670868	0,20355093	14,370318
GOTERM_MF_FAT	GO:0046872~metal ion binding	47	28,1437126	0,012039	ZCCHC24, CYP1B1, KCNAB1, ZFP738, LTBP4, SYT6, RORB, KCNIP2, KCNIP1, DCT, KCNQ5, TDO2, PLOD1, CALML4, SMOC1, PLCH2, SCN9A, ATP8B1, CYGB, PLCD1, ZFP273, NR2F2, CDH23, GALNT14, NR2F1, PTGR2, VAV3, CRTAC1, SPARCL1, SCUBE1, ZFP429, NR4A2, S100A10, ARHGAP29, CACNG2, SLIT3, SLC17A7, PGM2, ZFP827, ZDHHC14, PRICKLE1, HPCA, JAZF1, CACNA1G, GUCY1B3, KCNH3, PLA2G4B	119	3850	13288	1,363169268	0,97123958	0,19892218	14,8935466
GOTERM_CC_FAT	GO:0042995~cell projection	12	7,18562874	0,012264	NRP2, SEMA6A, ROBO1, LAYN, RASGRF1, NTRK2, CYGB, NRADD, CYS1, NEFL, CAMK2N1, CDH23	111	575	12504	2,35092832	0,88025878	0,34589309	14,0013916
GOTERM_BP_FAT	GO:0007267~cell-cell signaling	8	4,79041916	0,01268	SLC17A7, ATXN1, WNT7B, FGFR4, NTF3, LTBP4, NTRK2, DRD1A	118	290	13588	3,176621859	0,99999814	0,3216415	18,3294358
KEGG_PATHWAY	mmu04020:Calcium signaling pathway	7	4,19161677	0,013091	SLC8A3, TRHR, CACNA1I, CACNA1G, PLCD1, HTR2C, DRD1A	60	191	5738	3,504886562	0,70249081	0,45455597	13,407725
GOTERM_BP_FAT	GO:0006812~cation transport	11	6,58682635	0,013379	SLC8A3, SLC17A7, KCNQ5, KCNAB1, CACNA1I, CACNA1G, SCN9A, CACNG2, KCNIP2, KCNH3, KCNIP1	118	515	13588	2,459568866	0,99999911	0,32828854	19,2425873
GOTERM_MF_FAT	GO:0004714~transmembrane receptor protein tyrosine kinase activity	4	2,39520958	0,013447	FGFR4, FGFR3, NTRK2, EPHB3	119	56	13288	7,975990396	0,98106558	0,20811543	16,4949857
GOTERM_MF_FAT	GO:0004879~ligand-dependent nuclear receptor activity	4	2,39520958	0,013447	NR4A2, RORB, NR2F2, NR2F1	119	56	13288	7,975990396	0,98106558	0,20811543	16,4949857
GOTERM_BP_FAT	GO:0051969~regulation of transmission of nerve impulse	5	2,99401198	0,013659	NTF3, RASGRF1, NTRK2, HTR2C, DRD1A	118	107	13588	5,380959924	0,99999933	0,32633355	19,6052437
GOTERM_CC_FAT	GO:0030424~axon	5	2,99401198	0,014639	NRP2, SEMA6A, ROBO1, NTRK2, NEFL	111	107	12504	5,263955544	0,92085902	0,34476024	16,4954459

GOTERM_MF_FAT	GO:0005245~voltage-gated calcium channel activity	3	1,79640719	0,014707	CACNA1I, CACNA1G, CACNG2	119	21	13288	15,95198079	0,98697884	0,21429718	17,9037425
GOTERM_BP_FAT	GO:0008344~adult locomotory behavior	4	2,39520958	0,016281	ATXN1, NR4A2, DRD1A, CDH23	118	62	13588	7,429196282	0,99999996	0,36792255	22,9305651
GOTERM_BP_FAT	GO:0031644~regulation of neurological system process	5	2,99401198	0,01639	NTF3, RASGRF1, NTRK2, HTR2C, DRD1A	118	113	13588	5,095245238	0,99999996	0,36215803	23,0649816
GOTERM_BP_FAT	GO:0044093~positive regulation of molecular function	8	4,79041916	0,016585	VAV3, RASGRF1, APH1B, NR4A2, HTR2C, DRD1A, KITL, ADCYAP1	118	306	13588	3,010523984	0,99999997	0,35815925	23,3077143
GOTERM_CC_FAT	GO:0060076~excitatory synapse	2	1,19760479	0,017518	SLC17A7, NTRK2	111	2	12504	112,6486486	0,95215284	0,3522491	19,4290836
GOTERM_CC_FAT	GO:0044421~extracellular region part	14	8,38323353	0,018196	LPL, SPARCL1, CRTAC1, LTBP4, CCDC80, NTNG2, TIMP2, KITL, COL5A1, SLIT3, WNT7B, CXCL14, SMOC1, ANGPTL4	111	774	12504	2,037572456	0,95750877	0,32619007	20,1059956
GOTERM_MF_FAT	GO:0005539~glycosaminoglycan binding	5	2,99401198	0,018672	FGFBP3, LPL, LAYN, CCDC80, COL5A1	119	114	13288	4,897537963	0,99600432	0,25223023	22,1948719
GOTERM_BP_FAT	GO:0030814~regulation of cAMP metabolic process	4	2,39520958	0,019997	HRH3, TIMP2, DRD1A, ADCYAP1	118	67	13588	6,874778649	1	0,40676194	27,4219508
GOTERM_MF_FAT	GO:0030955~potassium ion binding	5	2,99401198	0,020905	KCNQ5, KCNAB1, KCNIP2, KCNH3, KCNIP1	119	118	13288	4,731519727	0,9979506	0,2661939	24,520104
GOTERM_CC_FAT	GO:0045202~synapse	8	4,79041916	0,022445	SLC17A7, SPARCL1, NTRK2, GLRA2, SYT6, FAIM2, CAMK2N1, CDH23	111	319	12504	2,82504448	0,97984793	0,35197601	24,2314128
GOTERM_BP_FAT	GO:0030799~regulation of cyclic nucleotide metabolic process	4	2,39520958	0,025022	HRH3, TIMP2, DRD1A, ADCYAP1	118	73	13588	6,309728349	1	0,47221495	33,1068101
GOTERM_BP_FAT	GO:0006873~cellular ion homeostasis	7	4,19161677	0,025311	ATXN1, NTF3, HEXB, CACNA1I, CACNA1G, CACNG2, DRD1A	118	261	13588	3,088382362	1	0,46802856	33,4214444
GOTERM_BP_FAT	GO:0051968~positive regulation of synaptic transmission, glutamatergic	2	1,19760479	0,025612	NTRK2, DRD1A	118	3	13588	76,76836158	1	0,46414736	33,746135
GOTERM_MF_FAT	GO:0005165~neurotrophin receptor binding	2	1,19760479	0,026407	NTF3, NRADD	119	3	13288	74,44257703	0,99960678	0,31160187	29,9757084
GOTERM_MF_FAT	GO:0005166~neurotrophin p75 receptor binding	2	1,19760479	0,026407	NTF3, NRADD	119	3	13288	74,44257703	0,99960678	0,31160187	29,9757084
GOTERM_MF_FAT	GO:0008332~low voltage-gated calcium channel activity	2	1,19760479	0,026407	CACNA1I, CACNA1G	119	3	13288	74,44257703	0,99960678	0,31160187	29,9757084
GOTERM_CC_FAT	GO:0005604~basement membrane	4	2,39520958	0,026411	SMOC1, CCDC80, TIMP2, COL5A1	111	73	12504	6,172528693	0,98998521	0,36894938	27,9046885
GOTERM_BP_FAT	GO:0006140~regulation of nucleotide metabolic process	4	2,39520958	0,026834	HRH3, TIMP2, DRD1A, ADCYAP1	118	75	13588	6,141468927	1	0,47229438	35,0527387
GOTERM_MF_FAT	GO:0030247~polysaccharide binding	5	2,99401198	0,027185	FGFBP3, LPL, LAYN, CCDC80, COL5A1	119	128	13288	4,361869748	0,99968889	0,30723325	30,7170619
GOTERM_MF_FAT	GO:0001871~pattern binding	5	2,99401198	0,027185	FGFBP3, LPL, LAYN, CCDC80, COL5A1	119	128	13288	4,361869748	0,99968889	0,30723325	30,7170619
GOTERM_MF_FAT	GO:0005267~potassium channel activity	5	2,99401198	0,027868	KCNQ5, KCNAB1, KCNIP2, KCNH3, KCNIP1	119	129	13288	4,328056804	0,99974679	0,30236274	31,3623843
GOTERM_BP_FAT	GO:0055082~cellular chemical homeostasis	7	4,19161677	0,028328	ATXN1, NTF3, HEXB, CACNA1I, CACNA1G, CACNG2, DRD1A	118	268	13588	3,007715659	1	0,48331328	36,6170457
GOTERM_BP_FAT	GO:0016042~lipid catabolic process	5	2,99401198	0,028527	LPL, PLCH2, HEXB, PLCD1, PLA2G4B	118	134	13588	4,296736656	1	0,4782514	36,8227711
GOTERM_BP_FAT	GO:0051705~behavioral interaction between organisms	3	1,79640719	0,029054	PPP1R1B, HEXB, DRD1A	118	31	13588	11,14379442	1	0,47725454	37,3641734
GOTERM_BP_FAT	GO:0044057~regulation of system process	6	3,59281437	0,029856	NTF3, RASGRF1, NTRK2, CACNA1G, HTR2C, DRD1A	118	201	13588	3,437389325	1	0,47948473	38,1795408
GOTERM_BP_FAT	GO:0060191~regulation of lipase activity	3	1,79640719	0,03082	HTR2C, DRD1A, ANGPTL4	118	32	13588	10,79555085	1	0,48345295	39,1470348
GOTERM_BP_FAT	GO:0043523~regulation of neuron apoptosis	4	2,39520958	0,031664	NMNAT3, NTF3, NR4A2, NEFL	118	80	13588	5,757627119	1	0,48593659	39,9831365
GOTERM_BP_FAT	GO:0021954~central nervous system neuron development	3	1,79640719	0,032627	NR4A2, EPHB3, DRD1A	118	33	13588	10,46841294	1	0,48957851	40,9224782
GOTERM_MF_FAT	GO:0005007~fibroblast growth factor receptor activity	2	1,19760479	0,035054	FGFR4, FGFR3	119	4	13288	55,83193277	0,9999712	0,35314669	37,8190769
GOTERM_MF_FAT	GO:0008201~heparin binding	4	2,39520958	0,037559	FGFBP3, LPL, CCDC80, COL5A1	119	83	13288	5,381391111	0,99998655	0,36152489	39,934205
GOTERM_BP_FAT	GO:0051899~membrane depolarization	3	1,79640719	0,038288	ATXN1, CACNA1G, CACNG2	118	36	13588	9,596045198	1	0,53989495	46,1761265
GOTERM_BP_FAT	GO:0048878~chemical homeostasis	8	4,79041916	0,038304	ATXN1, NTF3, HEXB, CACNA1I, CACNA1G, AQP4, CACNG2, DRD1A	118	365	13588	2,52389134	1	0,53325591	46,1901716
GOTERM_MF_FAT	GO:0016298~lipase activity	4	2,39520958	0,038706	LPL, PLCH2, PLCD1, PLA2G4B	119	84	13288	5,317326931	0,99999052	0,35907784	40,879894
GOTERM_CC_FAT	GO:0044456~synapse part	6	3,59281437	0,039146	SLC17A7, NTRK2, GLRA2, SYT6, FAIM2, CAMK2N1	111	212	12504	3,188169301	0,99895997	0,46442238	38,6232919
GOTERM_BP_FAT	GO:0043062~extracellular structure organization	5	2,99401198	0,039773	WNT7B, SMOC1, CCDC80, CACNG2, COL5A1	118	149	13588	3,864179274	1	0,54028047	47,4798247
GOTERM_BP_FAT	GO:0050801~ion homeostasis	7	4,19161677	0,041011	ATXN1, NTF3, HEXB, CACNA1I, CACNA1G, CACNG2, DRD1A	118	293	13588	2,75108463	1	0,54491213	48,5440731
GOTERM_BP_FAT	GO:0009404~toxin metabolic process	2	1,19760479	0,042324	CYP1B1, AS3MT	118	5	13588	46,06101695	1	0,5499954	49,6503255
GOTERM_BP_FAT	GO:0006811~ion transport	12	7,18562874	0,042922	SLC8A3, SLC17A7, KCNQ5, KCNAB1, GLRA2, CACNA1I, CACNA1G, SCN9A, CACNG2, KCNIP2, KCNH3, KCNIP1	118	712	13588	1,940773186	1	0,54879286	50,1472704
GOTERM_MF_FAT	GO:0043121~neurotrophin binding	2	1,19760479	0,043626	NTF3, NTRK2	119	5	13288	44,66554622	0,99999789	0,38372254	44,7844115
KEGG_PATHWAY	mmu04360:Axon guidance	5	2,99401198	0,04501	SEMA6A, UNC5A, ROBO1, EPHB3, SLIT3	60	131	5738	3,650127226	0,9855498	0,75642956	39,537176
GOTERM_BP_FAT	GO:0019226~transmission of nerve impulse	6	3,59281437	0,045692	SLC17A7, ATXN1, NTF3, HEXB, CACNG2, DRD1A	118	226	13588	3,057147143	1	0,56559726	52,3882038
GOTERM_BP_FAT	GO:0030203~glycosaminoglycan metabolic process	3	1,79640719	0,046368	CHST11, HEXB, CHST5	118	40	13588	8,636440678	1	0,56485103	52,920398
GOTERM_BP_FAT	GO:0015672~monovalent inorganic cation transport	7	4,19161677	0,046952	SLC17A7, KCNQ5, KCNAB1, SCN9A, KCNIP2, KCNH3, KCNIP1	118	303	13588	2,660289758	1	0,56340698	53,3756996
GOTERM_CC_FAT	GO:0044420~extracellular matrix part	4	2,39520958	0,047366	SMOC1, CCDC80, TIMP2, COL5A1	111	92	12504	4,897767333	0,9997627	0,5011821	44,741968
GOTERM_BP_FAT	GO:0030155~regulation of cell adhesion	4	2,39520958	0,047428	VAV3, PODXL, SMOC1, CCDC80	118	94	13588	4,900108186	1	0,56116423	53,7435647
GOTERM_BP_FAT	GO:0042981~regulation of apoptosis	10	5,98802395	0,049132	NMNAT3, FGFR3, NTF3, CSRNP3, CHST11, NR4A2, DMRT2, KITL, NEFL, ANGPTL4	118	553	13588	2,082324455	1	0,56838066	55,0394134
GOTERM_BP_FAT	GO:0006813~potassium ion transport	5	2,99401198	0,049417	KCNQ5, KCNAB1, KCNIP2, KCNH3, KCNIP1	118	160	13588	3,598516949	1	0,56472892	55,2523984
KEGG_PATHWAY	mmu04080:Neuroactive ligand-receptor interaction	7	4,19161677	0,049476	HRH3, TRHR, GLRA2, ADRA2C, NPY1R, HTR2C, DRD1A	60	260	5738	2,57474359	0,9906117	0,68872324	42,5555004
GOTERM_BP_FAT	GO:0021953~central nervous system neuron differentiation	3	1,79640719	0,050622	NR4A2, EPHB3, DRD1A	118	42	13588	8,225181598	1	0,56798432	56,1444576
GOTERM_BP_FAT	GO:0043067~regulation of programmed cell death	10	5,98802395	0,052426	NMNAT3, FGFR3, NTF3, CSRNP3, CHST11, NR4A2, DMRT2, KITL, NEFL, ANGPTL4	118	560	13588	2,0562954	1	0,57541121	57,4480593
GOTERM_CC_FAT	GO:0005829~cytosol	10	5,98802395	0,052593	DCT, CCND1, PRICKLE1, RPS17, RASGRF1, NTRK2, AS3MT, PLCD1, PLA2G4B, HSPA8	111	549	12504	2,051887954	0,99990789	0,51071345	48,3360951
GOTERM_BP_FAT	GO:0045785~positive regulation of cell adhesion	3	1,79640719	0,0528	VAV3, SMOC1, CCDC80	118	43	13588	8,033898305	1	0,57251686	57,7138011
GOTERM_BP_FAT	GO:0010941~regulation of cell death	10	5,98802395	0,053881	NMNAT3, FGFR3, NTF3, CSRNP3, CHST11, NR4A2, DMRT2, KITL, NEFL, ANGPTL4	118	563	13588	2,04533823	1	0,57462466	58,4732527
GOTERM_BP_FAT	GO:0030900~forebrain development	5	2,99401198	0,056168	FGFR3, EMX1, NR2F2, DRD1A, NR2F1	118	167	13588	3,447680909	1	0,58480505	60,0374088
GOTERM_BP_FAT	GO:0019228~regulation of action potential in neuron	3	1,79640719	0,057254	NTF3, HEXB, DRD1A	118	45	13588	7,676836158	1	0,58667415	60,760736
GOTERM_BP_FAT	GO:0051172~negative regulation of nitrogen compound metabolic process	8	4,79041916	0,058065	TXNIP, ATXN1, ID2, JAZF1, NR2F2, HTR2C, TCF7L1, NR2F1	118	401	13588	2,297307579	1	0,58671586	61,2933433
GOTERM_MF_FAT	GO:0030246~carbohydrate binding	7	4,19161677	0,063246	FGFBP3, LPL, LAYN, GLT28D2, CCDC80, COL5A1, GALNT14	119	317	13288	2,465763593	1	0,49524253	58,1015078
GOTERM_BP_FAT	GO:0042592~homeostatic process	10	5,98802395	0,064813	ATXN1, ID2, NTF3, HEXB, CACNA1I, CACNA1G, AQP4, CACNG2, DRD1A, FANCC	118	584	13588	1,971790109	1	0,62313861	65,4662966
GOTERM_MF_FAT	GO:0004857~enzyme inhibitor activity	6	3,59281437	0,065387	TXNIP, PPP1R1B, SPINT2, TIMP2, CAMK2N1, ANGPTL4	119	243	13288	2,757132483	1	0,49501452	59,3590401
GOTERM_MF_FAT	GO:0019841~retinol binding	2	1,19760479	0,06889	CRABP1, CRABP2	119	8	13288	27,91596639	1	0,50198376	61,3408846
GOTERM_MF_FAT	GO:0008146~sulfotransferase activity	3	1,79640719	0,070199	CHST11, HS6ST3, CHST5	119	49	13288	6,836563197	1	0,49738521	62,0582807
PANTHER_PATHWAY	P00009:Axon guidance mediated by netrin	3	1,79640719	0,070755	UNC5A, NTNG2, NEO1	34	39	2921	6,608597285	0,92876614	0,73310328	48,031026
GOTERM_BP_FAT	GO:0006022~aminoglycan metabolic process	3	1,79640719	0,073798	CHST11, HEXB, CHST5	118	52	13588	6,643415906	1	0,66745079	70,3709895
GOTERM_BP_FAT	GO:0033057~reproductive behavior in a multicellular organism	2	1,19760479	0,0749	PPP1R1B, HEXB	118	9	13588	25,58945386	1	0,66804169	70,9253384
GOTERM_BP_FAT	GO:0019725~cellular homeostasis	7	4,19161677	0,075871	ATXN1, NTF3, HEXB, CACNA1I, CACNA1G, CACNG2, DRD1A	118	343	13588	2,350051885	1	0,66796421	71,4056226
GOTERM_CC_FAT	GO:0044459~plasma membrane part	21	12,5748503	0,076675	SLC8A3, FGFR3, CLDN3, SCUBE1, GLRA2, AQP4, NTNG2, SYT6, EPHB3, CAMK2N1, SLC17A7, KCNQ5, ROBO1, LAYN, NTRK2, SCN9A, CACNA1G, HTR2C, PLA2G4B, ODZ3, FAIM2	111	1633	12504	1,448635408	0,9999989	0,62472002	62,2865471
GOTERM_BP_FAT	GO:0006355~regulation of transcription, DNA-dependent	19	11,3772455	0,076686	TXNIP, HLF, NTF3, EMX1, ZFP738, ZFP429, NR4A2, DMRT2, RORB, 6720489N17RIK, TCF7L1, ATXN1, ID2, CSRNP3, JAZF1, ZFP273, NR2F2, KCNH3, NR2F1	118	1465	13588	1,493445942	1	0,66712242	71,8032751
GOTERM_BP_FAT	GO:0010648~negative regulation of cell communication	5	2,99401198	0,076856	ATXN1, CCND1, CHST11, DRD1A, RGS9BP	118	186	13588	3,095498451	1	0,66311681	71,8857588
GOTERM_MF_FAT	GO:0016918~retinal binding	2	1,19760479	0,077163	CRABP1, CRABP2	119	9	13288	24,81419234	1	0,52062604	65,6726054
GOTERM_BP_FAT	GO:0001508~regulation of action potential	3	1,79640719	0,078776	NTF3, HEXB, DRD1A	118	54	13588	6,397363465	1	0,66774248	72,7992719
GOTERM_BP_FAT	GO:0042692~muscle cell differentiation	4	2,39520958	0,080074	NTF3, NEO1, CACNG2, FLNC	118	117	13588	3,936839055	1	0,66925474	73,4009532
GOTERM_BP_FAT	GO:0010647~positive regulation of cell communication	5	2,99401198	0,080431	FGFBP3, FGFR3, NTRK2, DRD1A, KITL	118	189	13588	3,046363555	1	0,66628978	73,5642767
GOTERM_BP_FAT	GO:0006024~glycosaminoglycan biosynthetic process	2	1,19760479	0,082871	CHST11, CHST5	118	10	13588	23,03050847	1	0,67310071	74,655534
KEGG_PATHWAY	mmu04916:Melanogenesis	4	2,39520958	0,082977	DCT, WNT7B, KITL, TCF7L1	60	100	5738	3,825333333	0,99965408	0,79685996	61,1840665
GOTERM_BP_FAT	GO:0044242~cellular lipid catabolic process	3	1,79640719	0,083856	LPL, HEXB, PLA2G4B	118	56	13588	6,168886199	1	0,67307507	75,0842469
GOTERM_BP_FAT	GO:0006357~regulation of transcription from RNA polymerase II promoter	10	5,98802395	0,084028	TXNIP, ATXN1, ID2, NTF3, CSRNP3, NR4A2, JAZF1, NR2F2, TCF7L1, NR2F1	118	616	13588	1,869359454	1	0,66936876	75,1581692
GOTERM_BP_FAT	GO:0007169~transmembrane receptor protein tyrosine kinase signaling pathway	5	2,99401198	0,084087	TXNIP, FGFR4, FGFR3, NTRK2, EPHB3	118	192	13588	2,998764124	1	0,66520068	75,1836806
GOTERM_MF_FAT	GO:0017134~fibroblast growth factor binding	2	1,19760479	0,085364	FGFBP3, FGFR3	119	10	13288	22,33277311	1	0,5471728	69,5192347
GOTERM_BP_FAT	GO:0051252~regulation of RNA metabolic process	19	11,3772455	0,086072	TXNIP, HLF, NTF3, EMX1, ZFP738, ZFP429, NR4A2, DMRT2, RORB, 6720489N17RIK, TCF7L1, ATXN1, ID2, CSRNP3, JAZF1, ZFP273, NR2F2, KCNH3, NR2F1	118	1488	13588	1,470361764	1	0,66974749	76,0232475
GOTERM_BP_FAT	GO:0006816~calcium ion transport	4	2,39520958	0,086539	SLC8A3, CACNA1I, CACNA1G, CACNG2	118	121	13588	3,806695616	1	0,66748734	76,2167897
GOTERM_BP_FAT	GO:0007167~enzyme linked receptor protein signaling pathway	6	3,59281437	0,08688	TXNIP, FGFR4, FGFR3, LTBP4, NTRK2, EPHB3	118	273	13588	2,530825107	1	0,66471198	76,3573372
BIOCARTA	m_ck1Pathway:Regulation of ck1/cdk5 by type 1 glutamate receptors	2	1,19760479	0,088829	PPP1R1B, DRD1A	10	12	1171	19,51666667	0,8708189	0,8708189	51,7146462
GOTERM_BP_FAT	GO:0051336~regulation of hydrolase activity	5	2,99401198	0,089088	VAV3, RASGRF1, HTR2C, DRD1A, ANGPTL4	118	196	13588	2,937564856	1	0,67010444	77,2483437
GOTERM_BP_FAT	GO:0021952~central nervous system projection neuron axonogenesis	2	1,19760479	0,090774	NR4A2, EPHB3	118	11	13588	20,93682589	1	0,67311423	77,9073806
GOTERM_BP_FAT	GO:0006023~aminoglycan biosynthetic process	2	1,19760479	0,090774	CHST11, CHST5	118	11	13588	20,93682589	1	0,67311423	77,9073806
GOTERM_MF_FAT	GO:0046982~protein heterodimerization activity	4	2,39520958	0,092843	ROBO1, SCUBE1, HEXB, NR4A2	119	121	13288	3,691367456	1	0,56815996	72,6758335
GOTERM_MF_FAT	GO:0016782~transferase activity, transferring sulfur-containing groups	3	1,79640719	0,093799	CHST11, HS6ST3, CHST5	119	58	13288	5,775717183	1	0,56156008	73,0566365
GOTERM_BP_FAT	GO:0045761~regulation of adenylate cyclase activity	3	1,79640719	0,094304	HRH3, DRD1A, ADCYAP1	118	60	13588	5,757627119	1	0,68361002	79,2299018
GOTERM_CC_FAT	GO:0005576~extracellular region	21	12,5748503	0,095894	FGFBP3, NXPH3, LPL, NTF3, SPARCL1, CRTAC1, LTBP4, SCUBE1, CCDC80, NTNG2, TIMP2, KITL, COL5A1, ADCYAP1, SLIT3, ST3GAL1, BTBD17, WNT7B, CXCL14, SMOC1, ANGPTL4	111	1680	12504	1,408108108	0,99999997	0,68523967	70,8373599
GOTERM_BP_FAT	GO:0051966~regulation of synaptic transmission, glutamatergic	2	1,19760479	0,098609	NTRK2, DRD1A	118	12	13588	19,1920904	1	0,6966086	80,7421915
GOTERM_BP_FAT	GO:0031279~regulation of cyclase activity	3	1,79640719	0,099662	HRH3, DRD1A, ADCYAP1	118	62	13588	5,571897212	1	0,69666197	81,0961023
GOTERM_BP_FAT	GO:0051339~regulation of lyase activity	3	1,79640719	0,099662	HRH3, DRD1A, ADCYAP1	118	62	13588	5,571897212	1	0,69666197	81,0961023

Appendix Table S3
GO analysis by IPA (Ingenuity Pathway Analysis)

© 2000-2020 QIAGEN. All rights reserved.

Ingenuity Canonical Pathways	-log(p-value)	Ratio	z-score	Molecules
Axonal Guidance Signaling	3,92E+00	2,47E-02	NaN	EPHB3,NRP2,NTF3,NTNG2,NTRK2,PLCD1,PLCH2,ROBO1,SEMA6A,SLIT3,UNC5A,WNT7B
Sperm Motility	3,84E+00	3,59E-02	NaN	CACNA1G,EPHB3,FGFR3,FGFR4,NTRK2,PLA2G4B,PLCD1,PLCH2
Human Embryonic Stem Cell Pluripotency	3,49E+00	4,44E-02	NaN	FGFR3,FGFR4,NTF3,NTRK2,TCF7L1,WNT7B
Regulation of the Epithelial-Mesenchymal Transition Pathway	3,46E+00	3,65E-02	NaN	APH1B,CLDN3,FGFR3,FGFR4,ID2,TCF7L1,WNT7B
Thyroid Cancer Signaling	3,40E+00	7,84E-02	NaN	CCND1,NTF3,NTRK2,TCF7L1
Netrin Signaling	3,00E+00	6,15E-02	-2	CACNA1G,CACNA1I,CACNG2,UNC5A
GPCR-Mediated Nutrient Sensing in Enteroendocrine Cells	3,00E+00	4,46E-02	-2,236	CACNA1G,CACNA1I,CACNG2,PLCD1,PLCH2
Endocannabinoid Neuronal Synapse Pathway	2,74E+00	3,91E-02	-2,236	CACNA1G,CACNA1I,CACNG2,PLCD1,PLCH2
Synaptic Long Term Depression	2,73E+00	3,17E-02	-2,449	CACNA1G,CACNA1I,CACNG2,PLA2G4B,PLCD1,PLCH2
White Adipose Tissue Browning Pathway	2,73E+00	3,88E-02	-1,342	CACNA1G,CACNA1I,CACNG2,FGFR3,FGFR4
Adipogenesis pathway	2,66E+00	3,73E-02	NaN	FGFR3,FGFR4,LPL,NR2F2,TXNIP
nNOS Signaling in Skeletal Muscle Cells	2,57E+00	7,32E-02	NaN	CACNA1G,CACNA1I,CACNG2
Aryl Hydrocarbon Receptor Signaling	2,53E+00	3,50E-02	NaN	ALDH1L1,CCND1,CYP1B1,DCT,NR2F1
Role of NFAT in Cardiac Hypertrophy	2,46E+00	2,80E-02	-1,633	CACNA1G,CACNA1I,CACNG2,PLCD1,PLCH2,SLC8A3
Dermatan Sulfate Biosynthesis (Late Stages)	2,43E+00	6,52E-02	NaN	CHST11,HS3ST4,HS6ST3
Chondroitin Sulfate Biosynthesis (Late Stages)	2,37E+00	6,25E-02	NaN	CHST11,HS3ST4,HS6ST3
Chondroitin Sulfate Biosynthesis	2,19E+00	5,36E-02	NaN	CHST11,HS3ST4,HS6ST3
Dermatan Sulfate Biosynthesis	2,12E+00	5,08E-02	NaN	CHST11,HS3ST4,HS6ST3
Phospholipases	2,05E+00	4,76E-02	NaN	PLA2G4B,PLCD1,PLCH2
IL-15 Production	2,03E+00	3,31E-02	NaN	EPHB3,FGFR3,FGFR4,NTRK2
PTEN Signaling	1,97E+00	3,17E-02	1	CCND1,FGFR3,FGFR4,NTRK2
Heparan Sulfate Biosynthesis (Late Stages)	1,91E+00	4,23E-02	NaN	CHST11,HS3ST4,HS6ST3
Melatonin Signaling	1,89E+00	4,17E-02	NaN	PLCD1,PLCH2,RORB
GPCR-Mediated Integration of Enteroendocrine Signaling Exemplified by an L Cell	1,87E+00	4,11E-02	NaN	ADCYAP1,PLCD1,PLCH2
CREB Signaling in Neurons	1,87E+00	2,42E-02	NaN	CACNA1G,CACNA1I,CACNG2,PLCD1,PLCH2
Androgen Signaling	1,86E+00	2,94E-02	-1	CACNA1G,CACNA1I,CACNG2,CCND1
FcγRIIB Signaling in B Lymphocytes	1,84E+00	4,00E-02	NaN	CACNA1G,CACNA1I,CACNG2
Heparan Sulfate Biosynthesis	1,80E+00	3,85E-02	NaN	CHST11,HS3ST4,HS6ST3
Synaptogenesis Signaling Pathway	1,70E+00	1,92E-02	0,816	CDH23,EPHB3,HSPA8,NTRK2,RASGRF1,SYT6
PKCθ Signaling in T Lymphocytes	1,67E+00	2,58E-02	NaN	CACNA1G,CACNA1I,CACNG2,VAV3
Aldosterone Signaling in Epithelial Cells	1,64E+00	2,53E-02	NaN	HSPA12A,HSPA8,PLCD1,PLCH2
Acute Myeloid Leukemia Signaling	1,64E+00	3,37E-02	NaN	CCND1,KITLG,TCF7L1
CCR5 Signaling in Macrophages	1,58E+00	3,19E-02	NaN	CACNA1G,CACNA1I,CACNG2
Glioblastoma Multiforme Signaling	1,58E+00	2,42E-02	-2	CCND1,PLCD1,PLCH2,WNT7B
Arsenate Detoxification I (Glutaredoxin)	1,57E+00	2,50E-01	NaN	AS3MT
GABA Receptor Signaling	1,57E+00	3,16E-02	NaN	CACNA1G,CACNA1I,CACNG2
Apelin Cardiomyocyte Signaling Pathway	1,53E+00	3,03E-02	NaN	PLCD1,PLCH2,SLC8A3
Neuropathic Pain Signaling In Dorsal Horn Neurons	1,50E+00	2,97E-02	NaN	NTRK2,PLCD1,PLCH2
Eumelanin Biosynthesis	1,48E+00	2,00E-01	NaN	DCT
Antioxidant Action of Vitamin C	1,42E+00	2,75E-02	NaN	PLA2G4B,PLCD1,PLCH2
Role of Oct4 in Mammalian Embryonic Stem Cell Pluripotency	1,41E+00	4,35E-02	NaN	NR2F1,NR2F2
Tryptophan Degradation to 2-amino-3-carboxymuconate Semialdehyde	1,40E+00	1,67E-01	NaN	TDO2
NAD Biosynthesis III	1,40E+00	1,67E-01	NaN	Nmnat3
PPARα/RXRα Activation	1,39E+00	2,11E-02	NaN	LPL,NR2F1,PLCD1,PLCH2
RAR Activation	1,37E+00	2,07E-02	NaN	CRABP1,CRABP2,NR2F1,NR2F2
Xenobiotic Metabolism Signaling	1,34E+00	1,74E-02	NaN	ALDH1L1,CHST11,CYP1B1,HS3ST4,HS6ST3
NAD Salvage Pathway III	1,33E+00	1,43E-01	NaN	Nmnat3
GDP-glucose Biosynthesis	1,33E+00	1,43E-01	NaN	PGM1
G Beta Gamma Signaling	1,30E+00	2,46E-02	NaN	CACNA1G,CACNA1I,CACNG2
Calcium Signaling	1,29E+00	1,94E-02	-1	CACNA1G,CACNA1I,CACNG2,SLC8A3
Glucose and Glucose-1-phosphate Degradation	1,28E+00	1,25E-01	NaN	PGM1
Superoxide Radicals Degradation	1,28E+00	1,25E-01	NaN	CYGB
Gαi Signaling	1,27E+00	2,40E-02	NaN	ADRA2C,HRH3,NPY1R
Protein Kinase A Signaling	1,27E+00	1,51E-02	-1	FLNC,PLCD1,PLCH2,PPP1R1B,PTPRR,TCF7L1
FXR/RXR Activation	1,27E+00	2,38E-02	NaN	FBP1,FGFR4,LPL
Cellular Effects of Sildenafil (Viagra)	1,22E+00	2,29E-02	NaN	CACNG2,PLCD1,PLCH2
Role of Macrophages, Fibroblasts and Endothelial Cells in Rheumatoid Arthritis	1,21E+00	1,60E-02	NaN	CCND1,PLCD1,PLCH2,TCF7L1,WNT7B
Retinoic acid Mediated Apoptosis Signaling	1,20E+00	3,33E-02	NaN	CRABP1,CRABP2
PCP pathway	1,20E+00	3,33E-02	NaN	PRICKLE1,WNT7B
STAT3 Pathway	1,19E+00	2,22E-02	NaN	FGFR3,FGFR4,NTRK2
LPS/IL-1 Mediated Inhibition of RXR Function	1,18E+00	1,79E-02	NaN	ALDH1L1,CHST11,HS3ST4,HS6ST3
EIF2 Signaling	1,18E+00	1,79E-02	NaN	CCND1,RPL13,RPL35A,RPS17
Wnt/Ca+ pathway	1,18E+00	3,23E-02	NaN	PLCD1,PLCH2
PI3K Signaling in B Lymphocytes	1,17E+00	2,17E-02	NaN	PLCD1,PLCH2,VAV3
Ovarian Cancer Signaling	1,16E+00	2,16E-02	NaN	CCND1,TCF7L1,WNT7B
Type II Diabetes Mellitus Signaling	1,14E+00	2,11E-02	NaN	CACNA1G,CACNA1I,CACNG2
Corticotropin Releasing Hormone Signaling	1,12E+00	2,07E-02	NaN	CACNA1G,CACNA1I,CACNG2
Hematopoiesis from Multipotent Stem Cells	1,11E+00	8,33E-02	NaN	KITLG
Glycogen Degradation II	1,11E+00	8,33E-02	NaN	PGM1
NAD biosynthesis II (from tryptophan)	1,07E+00	7,69E-02	NaN	TDO2
Basal Cell Carcinoma Signaling	1,07E+00	2,78E-02	NaN	TCF7L1,WNT7B
Ephrin B Signaling	1,07E+00	2,78E-02	NaN	EPHB3,VAV3
Gustation Pathway	1,06E+00	1,95E-02	NaN	CACNA1G,CACNA1I,CACNG2
Leptin Signaling in Obesity	1,05E+00	2,70E-02	NaN	PLCD1,PLCH2
Glycogen Degradation III	1,04E+00	7,14E-02	NaN	PGM1
eNOS Signaling	1,03E+00	1,89E-02	NaN	AQP1,AQP4,HSPA8
HOTAIR Regulatory Pathway	1,03E+00	1,89E-02	NaN	ATXN1,TCF7L1,WNT7B
Neurotrophin/TRK Signaling	1,03E+00	2,63E-02	NaN	NTF3,NTRK2
Role of Wnt/GSK-3β Signaling in the Pathogenesis of Influenza	1,01E+00	2,56E-02	NaN	TCF7L1,WNT7B
Dopamine-DARPP32 Feedback in cAMP Signaling	1,00E+00	1,84E-02	NaN	PLCD1,PLCH2,PPP1R1B

Chondroitin Sulfate Degradation (Metazoa)	9,87E-01	6,25E-02	NaN	HEXB
Dermatan Sulfate Degradation (Metazoa)	9,63E-01	5,88E-02	NaN	HEXB
VEGF Family Ligand-Receptor Interactions	9,55E-01	2,38E-02	NaN	NRP2,PLA2G4B
FGF Signaling	9,55E-01	2,38E-02	NaN	FGFR3,FGFR4
GNRH Signaling	9,47E-01	1,73E-02	NaN	CACNA1G,CACNA1I,CACNG2
Wnt/β-catenin Signaling	9,47E-01	1,73E-02	NaN	CCND1,TCF7L1,WNT7B
G-Protein Coupled Receptor Signaling	9,43E-01	1,47E-02	NaN	ADRA2C,HRH3,HTR2C,NPY1R
Cardiac Hypertrophy Signaling (Enhanced)	9,43E-01	1,23E-02	-1,633	ADRA2C,FGFR3,FGFR4,PLCD1,PLCH2,WNT7B
GADD45 Signaling	9,17E-01	5,26E-02	NaN	CCND1
Granulocyte Adhesion and Diapedesis	9,14E-01	1,68E-02	NaN	CLDN3,CXCL14,HRH3
NF-κB Signaling	9,14E-01	1,68E-02	NaN	FGFR3,FGFR4,NTRK2
Hepatic Fibrosis / Hepatic Stellate Cell Activation	8,79E-01	1,61E-02	NaN	COL5A1,COL9A3,TIMP2
Melanocyte Development and Pigmentation Signaling	8,76E-01	2,13E-02	NaN	DCT,KITLG
Endothelin-1 Signaling	8,66E-01	1,60E-02	NaN	PLA2G4B,PLCD1,PLCH2
Bladder Cancer Signaling	8,54E-01	2,06E-02	NaN	CCND1,FGFR3
UVA-Induced MAPK Signaling	8,45E-01	2,04E-02	NaN	PLCD1,PLCH2
Tryptophan Degradation III (Eukaryotic)	8,39E-01	4,35E-02	NaN	TDO2
Ethanol Degradation IV	8,39E-01	4,35E-02	NaN	CYGB
Leukocyte Extravasation Signaling	8,24E-01	1,52E-02	NaN	CLDN3,TIMP2,VAV3
Gap Junction Signaling	8,21E-01	1,52E-02	NaN	HTR2C,PLCD1,PLCH2
Mouse Embryonic Stem Cell Pluripotency	8,12E-01	1,94E-02	NaN	ID2,TCF7L1
Bupropion Degradation	8,07E-01	4,00E-02	NaN	CYP1B1
D-myo-inositol (1,4,5)-Trisphosphate Biosynthesis	8,07E-01	4,00E-02	NaN	PLCD1
Estrogen-mediated S-phase Entry	7,90E-01	3,85E-02	NaN	CCND1
NAD Salvage Pathway II	7,90E-01	3,85E-02	NaN	Nmnat3
Glycolysis I	7,90E-01	3,85E-02	NaN	FBP1
Gluconeogenesis I	7,90E-01	3,85E-02	NaN	FBP1
CDK5 Signaling	7,80E-01	1,85E-02	NaN	NTRK2,PPP1R1B
Acetone Degradation I (to Methylglyoxal)	7,33E-01	3,33E-02	NaN	CYP1B1
Fc Epsilon RI Signaling	7,26E-01	1,71E-02	NaN	PLA2G4B,VAV3
Sphingosine-1-phosphate Signaling	7,26E-01	1,71E-02	NaN	PLCD1,PLCH2
Role of NANOG in Mammalian Embryonic Stem Cell Pluripotency	7,14E-01	1,68E-02	NaN	TCF7L1,WNT7B
GP6 Signaling Pathway	7,14E-01	1,68E-02	NaN	COL5A1,COL9A3
Circadian Rhythm Signaling	6,97E-01	3,03E-02	NaN	ADCYAP1
cAMP-mediated signaling	6,97E-01	1,32E-02	NaN	ADRA2C,HRH3,NPY1R
MIF-mediated Glucocorticoid Regulation	6,86E-01	2,94E-02	NaN	PLA2G4B
Phagosome Formation	6,82E-01	1,60E-02	NaN	PLCD1,PLCH2
Atherosclerosis Signaling	6,78E-01	1,59E-02	NaN	LPL,PLA2G4B
14-3-3-mediated Signaling	6,72E-01	1,57E-02	NaN	PLCD1,PLCH2
P2Y Purigenic Receptor Signaling Pathway	6,72E-01	1,57E-02	NaN	PLCD1,PLCH2
Interferon Signaling	6,64E-01	2,78E-02	NaN	IFITM2
Synaptic Long Term Potentiation	6,62E-01	1,55E-02	NaN	PLCD1,PLCH2
p70S6K Signaling	6,62E-01	1,55E-02	NaN	PLCD1,PLCH2
Gα12/13 Signaling	6,58E-01	1,54E-02	NaN	CDH23,VAV3
Cardiac Hypertrophy Signaling	6,54E-01	1,25E-02	NaN	ADRA2C,PLCD1,PLCH2
Cell Cycle Regulation by BTG Family Proteins	6,54E-01	2,70E-02	NaN	CCND1
Notch Signaling	6,54E-01	2,70E-02	NaN	APH1B
Inhibition of Matrix Metalloproteases	6,33E-01	2,56E-02	NaN	TIMP2
Opioid Signaling Pathway	6,29E-01	1,21E-02	NaN	CACNA1G,CACNA1I,CACNG2
Estrogen Biosynthesis	6,14E-01	2,44E-02	NaN	CYP1B1
Hereditary Breast Cancer Signaling	6,11E-01	1,43E-02	NaN	CCND1,FANCC
Colorectal Cancer Metastasis Signaling	6,09E-01	1,19E-02	NaN	CCND1,TCF7L1,WNT7B
MIF Regulation of Innate Immunity	6,04E-01	2,38E-02	NaN	PLA2G4B
Retinol Biosynthesis	6,04E-01	2,38E-02	NaN	LPL
Endocannabinoid Cancer Inhibition Pathway	5,97E-01	1,40E-02	NaN	CCND1,TCF7L1
Serotonin Receptor Signaling	5,95E-01	2,33E-02	NaN	HTR2C
BAG2 Signaling Pathway	5,95E-01	2,33E-02	NaN	HSPA8
PFKFB4 Signaling Pathway	5,70E-01	2,17E-02	NaN	FBP1
Molecular Mechanisms of Cancer	5,62E-01	1,02E-02	NaN	APH1B,CCND1,RASGRF1,WNT7B
Ephrin A Signaling	5,62E-01	2,13E-02	NaN	VAV3
Triacylglycerol Degradation	5,62E-01	2,13E-02	NaN	LPL
Hematopoiesis from Pluripotent Stem Cells	5,48E-01	2,04E-02	NaN	KITLG
D-myo-inositol-5-phosphate Metabolism	5,48E-01	1,29E-02	NaN	PLCD1,PPP1R1B
Melanoma Signaling	5,41E-01	2,00E-02	NaN	CCND1
Amyloid Processing	5,41E-01	2,00E-02	NaN	APH1B
Phototransduction Pathway	5,19E-01	1,89E-02	NaN	RGS9BP
Transcriptional Regulatory Network in Embryonic Stem Cells	5,13E-01	1,85E-02	NaN	TCF7L1
Nicotine Degradation III	4,99E-01	1,79E-02	NaN	CYP1B1
Unfolded protein response	4,99E-01	1,79E-02	NaN	HSPA8
Glutamate Receptor Signaling	4,93E-01	1,75E-02	NaN	SLC17A7
Neuroinflammation Signaling Pathway	4,79E-01	1,00E-02	NaN	APH1B,NTF3,PLA2G4B
Endometrial Cancer Signaling	4,75E-01	1,67E-02	NaN	CCND1
Melatonin Degradation I	4,75E-01	1,67E-02	NaN	CYP1B1
Acute Phase Response Signaling	4,66E-01	1,12E-02	NaN	CRABP1,CRABP2
Dendritic Cell Maturation	4,53E-01	1,09E-02	NaN	PLCD1,PLCH2
Hepatic Cholestasis	4,50E-01	1,09E-02	NaN	ATP8B1,FGFR4
ErbB2-ErbB3 Signaling	4,47E-01	1,54E-02	NaN	CCND1
Nicotine Degradation II	4,47E-01	1,54E-02	NaN	CYP1B1
Superpathway of Melatonin Degradation	4,47E-01	1,54E-02	NaN	CYP1B1
Regulation of Cellular Mechanics by Calpain Protease	4,47E-01	1,54E-02	NaN	CCND1
Eicosanoid Signaling	4,41E-01	1,52E-02	NaN	PLA2G4B
ErbB4 Signaling	4,37E-01	1,49E-02	NaN	APH1B
Cell Cycle: G1/S Checkpoint Regulation	4,37E-01	1,49E-02	NaN	CCND1
ILK Signaling	4,33E-01	1,05E-02	NaN	CCND1,FLNC
Remodeling of Epithelial Adherens Junctions	4,32E-01	1,47E-02	NaN	DNM3
Agranulocyte Adhesion and Diapedesis	4,27E-01	1,04E-02	NaN	CLDN3,CXCL14
Clathrin-mediated Endocytosis Signaling	4,24E-01	1,04E-02	NaN	DNM3,HSPA8
GM-CSF Signaling	4,21E-01	1,43E-02	NaN	CCND1
Small Cell Lung Cancer Signaling	4,17E-01	1,41E-02	NaN	CCND1
Superpathway of Inositol Phosphate Compounds	4,13E-01	1,02E-02	NaN	PLCD1,PPP1R1B
Adrenomedullin signaling pathway	4,13E-01	1,02E-02	NaN	PLCD1,PLCH2
Caveolar-mediated Endocytosis Signaling	4,07E-01	1,37E-02	NaN	FLNC

Non-Small Cell Lung Cancer Signaling	4,07E-01	1,37E-02	NaN	CCND1
Estrogen-Dependent Breast Cancer Signaling	4,02E-01	1,35E-02	NaN	CCND1
Glioma Invasiveness Signaling	4,02E-01	1,35E-02	NaN	TIMP2
Dopamine Receptor Signaling	3,89E-01	1,30E-02	NaN	PPP1R1B
Thrombin Signaling	3,85E-01	9,62E-03	NaN	PLCD1,PLCH2
IL-7 Signaling Pathway	3,85E-01	1,28E-02	NaN	CCND1
Osteoarthritis Pathway	3,78E-01	9,48E-03	NaN	FGFR3,TCF7L1
Role of BRCA1 in DNA Damage Response	3,77E-01	1,25E-02	NaN	FANCC
Role of MAPK Signaling in the Pathogenesis of Influenza	3,77E-01	1,25E-02	NaN	PLA2G4B
AMPK Signaling	3,75E-01	9,43E-03	NaN	ADRA2C,CCND1
Cyclins and Cell Cycle Regulation	3,73E-01	1,23E-02	NaN	CCND1
PEDF Signaling	3,69E-01	1,22E-02	NaN	TCF7L1
HER-2 Signaling in Breast Cancer	3,61E-01	1,19E-02	NaN	CCND1
HIPPO signaling	3,58E-01	1,18E-02	NaN	WWC1
Role of Osteoblasts, Osteoclasts and Chondrocytes in Rheumatoid Arthritis	3,57E-01	9,09E-03	NaN	TCF7L1,WNT7B
Regulation of IL-2 Expression in Activated and Anergic T Lymphocytes	3,43E-01	1,12E-02	NaN	VAV3
Hepatic Fibrosis Signaling Pathway	3,41E-01	8,15E-03	NaN	CCND1,TCF7L1,WNT7B
Prostate Cancer Signaling	3,35E-01	1,10E-02	NaN	CCND1
Factors Promoting Cardiogenesis in Vertebrates	3,29E-01	1,08E-02	NaN	TCF7L1
Fcγ Receptor-mediated Phagocytosis in Macrophages and Monocytes	3,25E-01	1,06E-02	NaN	VAV3
α-Adrenergic Signaling	3,22E-01	1,05E-02	NaN	SLC8A3
Huntington's Disease Signaling	3,21E-01	8,44E-03	NaN	DNM3,HSPA8
Amyotrophic Lateral Sclerosis Signaling	3,16E-01	1,03E-02	NaN	NEFL
PAK Signaling	3,16E-01	1,03E-02	NaN	EPHB3
p53 Signaling	3,12E-01	1,02E-02	NaN	CCND1
Chronic Myeloid Leukemia Signaling	2,98E-01	9,71E-03	NaN	CCND1
PPAR Signaling	2,94E-01	9,62E-03	NaN	NR2F1
T Cell Receptor Signaling	2,92E-01	9,52E-03	NaN	VAV3
Virus Entry via Endocytic Pathways	2,86E-01	9,35E-03	NaN	FLNC
Phospholipase C Signaling	2,83E-01	7,78E-03	NaN	PLA2G4B,PLCD1
Pancreatic Adenocarcinoma Signaling	2,81E-01	9,17E-03	NaN	CCND1
Glioma Signaling	2,78E-01	9,09E-03	NaN	CCND1
HGF Signaling	2,75E-01	9,01E-03	NaN	CCND1
Endocannabinoid Developing Neuron Pathway	2,65E-01	8,70E-03	NaN	CCND1
p38 MAPK Signaling	2,58E-01	8,47E-03	NaN	PLA2G4B
Protein Ubiquitination Pathway	2,56E-01	7,33E-03	NaN	HSPA12A,HSPA8
Natural Killer Cell Signaling	2,55E-01	8,40E-03	NaN	VAV3
LXR/RXR Activation	2,50E-01	8,26E-03	NaN	LPL
Th1 Pathway	2,50E-01	8,26E-03	NaN	APH1B
RhoA Signaling	2,46E-01	8,13E-03	NaN	NRP2
CCR3 Signaling in Eosinophils	2,44E-01	8,06E-03	NaN	PLA2G4B
Th2 Pathway	2,19E-01	7,35E-03	NaN	APH1B
Estrogen Receptor Signaling	2,17E-01	7,30E-03	NaN	NR0B1
D-myo-inositol (1,4,5,6)-Tetrakisphosphate Biosynthesis	2,11E-01	7,14E-03	NaN	PPP1R1B
D-myo-inositol (3,4,5,6)-tetrakisphosphate Biosynthesis	2,11E-01	7,14E-03	NaN	PPP1R1B
Cardiac β-adrenergic Signaling	2,09E-01	7,09E-03	NaN	SLC8A3
Glucocorticoid Receptor Signaling	0,00E+00	2,98E-03	NaN	HSPA8
Actin Cytoskeleton Signaling	0,00E+00	4,59E-03	NaN	VAV3
Mitochondrial Dysfunction	0,00E+00	5,85E-03	NaN	APH1B
Tight Junction Signaling	0,00E+00	5,95E-03	NaN	CLDN3
IL-8 Signaling	0,00E+00	5,00E-03	NaN	CCND1
mTOR Signaling	0,00E+00	4,76E-03	NaN	RPS17
Regulation of eIF4 and p70S6K Signaling	0,00E+00	6,37E-03	NaN	RPS17
Signaling by Rho Family GTPases	0,00E+00	4,10E-03	NaN	CDH23
RhoGDI Signaling	0,00E+00	5,56E-03	NaN	CDH23
Sertoli Cell-Sertoli Cell Junction Signaling	0,00E+00	5,41E-03	NaN	CLDN3
3-phosphoinositide Degradation	0,00E+00	6,49E-03	NaN	PPP1R1B
3-phosphoinositide Biosynthesis	0,00E+00	6,10E-03	NaN	PPP1R1B
Epithelial Adherens Junction Signaling	0,00E+00	6,58E-03	NaN	TCF7L1
Gαq Signaling	0,00E+00	6,37E-03	NaN	HTR2C
Tec Kinase Signaling	0,00E+00	6,10E-03	NaN	VAV3
ERK/MAPK Signaling	0,00E+00	5,18E-03	NaN	PLA2G4B
PI3K/AKT Signaling	0,00E+00	5,75E-03	NaN	CCND1
B Cell Receptor Signaling	0,00E+00	5,41E-03	NaN	VAV3
Th1 and Th2 Activation Pathway	0,00E+00	5,85E-03	NaN	APH1B
Systemic Lupus Erythematosus In B Cell Signaling Pathway	0,00E+00	3,64E-03	NaN	CCND1
Senescence Pathway	0,00E+00	3,64E-03	NaN	CCND1
Ephrin Receptor Signaling	0,00E+00	5,56E-03	NaN	EPHB3

Appendix Table S4
GO analysis by PANTHER Overrepresentation Test

Analysis Type:	PANTHER Overrepresentation Test (Released 20190711)
Annotation Version and Release Date:	GO Ontology database Released 2020-01-03
Analyzed List:	Client Text Box Input (Mus musculus)
Reference List:	Mus musculus (all genes in database)
Test Type:	FISHER
Correction:	FDR

GO biological process complete	Mus musculus - REFLIST (22296)	Client Text Box Input (168)	Client Text Box Input (expected)	Client Text Box Input (over/under)	Client Text Box Input (fold Enrichment)	Client Text Box Input (raw P-value)	Client Text Box Input (FDR)
regulation of biological quality (GO:0065008)	3956	66	29,81	+	2,21	8,50E-11	1,34E-06
system development (GO:0048731)	4219	68	31,79	+	2,14	1,41E-10	1,11E-06
neurogenesis (GO:0022008)	1771	39	13,34	+	2,92	1,23E-09	6,48E-06
neuron development (GO:0048666)	842	26	6,34	+	4,1	1,60E-09	6,32E-06
anatomical structure development (GO:0048856)	5208	75	39,24	+	1,91	2,14E-09	6,78E-06
generation of neurons (GO:0048699)	1663	37	12,53	+	2,95	2,76E-09	7,28E-06
animal organ development (GO:0048513)	3043	53	22,93	+	2,31	2,83E-09	6,39E-06
cell development (GO:0048468)	1749	38	13,18	+	2,88	3,05E-09	6,02E-06
nervous system development (GO:0007399)	2264	44	17,06	+	2,58	3,68E-09	6,47E-06
neuron differentiation (GO:0030182)	1036	28	7,81	+	3,59	6,09E-09	9,63E-06
developmental process (GO:0032502)	5565	76	41,93	+	1,81	1,61E-08	2,32E-05
multicellular organism development (GO:0007275)	4838	69	36,45	+	1,89	1,77E-08	2,33E-05
axon development (GO:0061564)	373	16	2,81	+	5,69	4,05E-08	4,92E-05
cellular developmental process (GO:0048869)	3785	58	28,52	+	2,03	4,33E-08	4,89E-05
cell differentiation (GO:0030154)	3699	57	27,87	+	2,05	5,64E-08	5,95E-05
forebrain development (GO:0030900)	383	16	2,89	+	5,54	5,74E-08	5,67E-05
head development (GO:0060322)	678	21	5,11	+	4,11	6,57E-08	6,11E-05
anatomical structure morphogenesis (GO:0009653)	2214	41	16,68	+	2,46	7,36E-08	6,46E-05
brain development (GO:0007420)	624	20	4,7	+	4,25	8,12E-08	6,76E-05
biological regulation (GO:0065007)	12132	125	91,41	+	1,37	1,32E-07	1,04E-04
cell morphogenesis (GO:0000902)	733	21	5,52	+	3,8	2,32E-07	1,75E-04
locomotion (GO:0040011)	1168	27	8,8	+	3,07	2,62E-07	1,89E-04
regulation of developmental process (GO:0050793)	2686	45	20,24	+	2,22	2,84E-07	1,95E-04
neuron projection development (GO:0031175)	691	20	5,21	+	3,84	3,94E-07	2,59E-04
regulation of multicellular organismal process (GO:0051239)	3224	50	24,29	+	2,06	4,24E-07	2,68E-04
cell morphogenesis involved in differentiation (GO:0000904)	569	18	4,29	+	4,2	4,54E-07	2,76E-04
central nervous system development (GO:0007417)	835	22	6,29	+	3,5	4,68E-07	2,74E-04
cellular component morphogenesis (GO:0032989)	841	22	6,34	+	3,47	5,26E-07	2,97E-04
movement of cell or subcellular component (GO:0006928)	1379	29	10,39	+	2,79	6,09E-07	3,32E-04
synapse organization (GO:0050808)	296	13	2,23	+	5,83	6,18E-07	3,26E-04
tissue development (GO:0009888)	1652	32	12,45	+	2,57	8,57E-07	4,37E-04
cell-cell signaling (GO:0007267)	808	21	6,09	+	3,45	1,08E-06	5,33E-04
negative regulation of biological process (GO:0048519)	5167	67	38,93	+	1,72	1,44E-06	6,91E-04
cell morphogenesis involved in neuron differentiation (GO:0048667)	433	15	3,26	+	4,6	1,48E-06	6,89E-04
behavior (GO:0007610)	687	19	5,18	+	3,67	1,51E-06	6,83E-04
regulation of catalytic activity (GO:0050790)	1874	34	14,12	+	2,41	1,88E-06	8,26E-04
regulation of nervous system development (GO:0051960)	1070	24	8,06	+	2,98	2,20E-06	9,38E-04
regulation of molecular function (GO:0065009)	2552	41	19,23	+	2,13	2,55E-06	1,06E-03
regulation of biological process (GO:0050789)	11515	117	86,77	+	1,35	2,73E-06	1,11E-03
axonogenesis (GO:0007409)	342	13	2,58	+	5,04	2,89E-06	1,14E-03
neuron projection morphogenesis (GO:0048812)	470	15	3,54	+	4,24	3,91E-06	1,51E-03
transmembrane transport (GO:0055085)	884	21	6,66	+	3,15	4,27E-06	1,61E-03
plasma membrane bounded cell projection morphogenesis (GO:0120039)	475	15	3,58	+	4,19	4,42E-06	1,63E-03
cell projection morphogenesis (GO:0048858)	481	15	3,62	+	4,14	5,12E-06	1,84E-03
response to chemical (GO:0042221)	3485	50	26,26	+	1,9	5,33E-06	1,87E-03
plasma membrane bounded cell projection organization (GO:0120036)	1053	23	7,93	+	2,9	5,60E-06	1,93E-03
circulatory system development (GO:0072359)	908	21	6,84	+	3,07	6,39E-06	2,15E-03
cell migration (GO:0016477)	841	20	6,34	+	3,16	7,25E-06	2,39E-03
metal ion transport (GO:0030001)	566	16	4,26	+	3,75	8,17E-06	2,64E-03
negative regulation of cellular process (GO:0048523)	4630	60	34,89	+	1,72	8,93E-06	2,82E-03
axon guidance (GO:0007411)	219	10	1,65	+	6,06	9,19E-06	2,85E-03
cell part morphogenesis (GO:0032990)	507	15	3,82	+	3,93	9,42E-06	2,86E-03
neuron projection guidance (GO:0097485)	221	10	1,67	+	6,01	9,92E-06	2,96E-03
regulation of membrane potential (GO:0042391)	446	14	3,36	+	4,17	9,96E-06	2,92E-03
multicellular organismal process (GO:0032501)	7289	83	54,92	+	1,51	1,00E-05	2,89E-03
cell projection organization (GO:0030030)	1109	23	8,36	+	2,75	1,27E-05	3,59E-03
regulation of neurogenesis (GO:0050767)	953	21	7,18	+	2,92	1,31E-05	3,62E-03
cation transport (GO:0006812)	733	18	5,52	+	3,26	1,41E-05	3,85E-03
localization of cell (GO:0051674)	961	21	7,24	+	2,9	1,48E-05	3,95E-03
cell motility (GO:0048870)	961	21	7,24	+	2,9	1,48E-05	3,89E-03
blood vessel development (GO:0001568)	528	15	3,98	+	3,77	1,50E-05	3,88E-03
transmission of nerve impulse (GO:0019226)	65	6	0,49	+	12,25	1,53E-05	3,91E-03
regulation of cell differentiation (GO:0045595)	1872	32	14,11	+	2,27	1,68E-05	4,21E-03
regulation of phosphate metabolic process (GO:0019220)	1730	30	13,04	+	2,3	1,82E-05	4,49E-03
regulation of phosphorus metabolic process (GO:0051174)	1731	30	13,04	+	2,3	1,83E-05	4,46E-03
neuron migration (GO:0001764)	143	8	1,08	+	7,42	1,86E-05	4,45E-03
positive regulation of developmental process (GO:0051094)	1541	28	11,61	+	2,41	1,99E-05	4,70E-03

memory (GO:0007613)	146	8	1,1	+	7,27	2,14E-05	4,98E-03
regulation of cytosolic calcium ion concentration (GO:0051480)	356	12	2,68	+	4,47	2,23E-05	5,12E-03
telencephalon development (GO:0021537)	248	10	1,87	+	5,35	2,57E-05	5,81E-03
vasculature development (GO:0001944)	557	15	4,2	+	3,57	2,74E-05	6,10E-03
regulation of cell development (GO:0060284)	1092	22	8,23	+	2,67	3,08E-05	6,76E-03
regulation of dopamine metabolic process (GO:0042053)	21	4	0,16	+	25,28	3,38E-05	7,31E-03
trans-synaptic signaling (GO:0099537)	372	12	2,8	+	4,28	3,39E-05	7,23E-03
cardiovascular system development (GO:0072358)	571	15	4,3	+	3,49	3,61E-05	7,62E-03
calcium ion transport into cytosol (GO:0060402)	77	6	0,58	+	10,34	3,75E-05	7,80E-03
central nervous system neuron differentiation (GO:0021953)	208	9	1,57	+	5,74	3,89E-05	7,99E-03
cellular response to chemical stimulus (GO:0070887)	2361	36	17,79	+	2,02	4,25E-05	8,61E-03
inorganic ion transmembrane transport (GO:0098660)	445	13	3,35	+	3,88	4,29E-05	8,59E-03
chemical homeostasis (GO:0048878)	1120	22	8,44	+	2,61	4,46E-05	8,82E-03
positive regulation of nervous system development (GO:0051962)	654	16	4,93	+	3,25	4,53E-05	8,85E-03
regulation of catecholamine metabolic process (GO:0042069)	23	4	0,17	+	23,08	4,63E-05	8,93E-03
drug transport (GO:0015893)	120	7	0,9	+	7,74	4,86E-05	9,26E-03
positive regulation of multicellular organismal process (GO:0051240)	1915	31	14,43	+	2,15	4,86E-05	9,15E-03
cellular calcium ion homeostasis (GO:0006874)	464	13	3,5	+	3,72	6,49E-05	1,21E-02
regulation of hydrolase activity (GO:0051336)	992	20	7,47	+	2,68	7,19E-05	1,32E-02
ion transport (GO:0006811)	1159	22	8,73	+	2,52	7,32E-05	1,33E-02
synaptic signaling (GO:0099536)	405	12	3,05	+	3,93	7,47E-05	1,34E-02
inorganic cation transmembrane transport (GO:0098662)	407	12	3,07	+	3,91	7,82E-05	1,39E-02
regulation of synapse structure or activity (GO:0050803)	285	10	2,15	+	4,66	7,94E-05	1,39E-02
regulation of cell population proliferation (GO:0042127)	1657	28	12,49	+	2,24	8,22E-05	1,43E-02
positive regulation of biological process (GO:0048518)	6109	70	46,03	+	1,52	8,27E-05	1,42E-02
tube development (GO:0035295)	922	19	6,95	+	2,73	8,33E-05	1,42E-02
response to drug (GO:0042493)	770	17	5,8	+	2,93	8,97E-05	1,51E-02
calcium ion homeostasis (GO:0055074)	480	13	3,62	+	3,59	9,04E-05	1,50E-02
taxis (GO:0042330)	480	13	3,62	+	3,59	9,04E-05	1,49E-02
response to wounding (GO:0009611)	352	11	2,65	+	4,15	9,50E-05	1,55E-02
response to inorganic substance (GO:0010035)	416	12	3,13	+	3,83	9,55E-05	1,54E-02
regulation of cellular process (GO:0050794)	10818	107	81,51	+	1,31	9,71E-05	1,55E-02
small molecule metabolic process (GO:0044281)	1433	25	10,8	+	2,32	9,85E-05	1,56E-02
positive regulation of catalytic activity (GO:0043085)	1100	21	8,29	+	2,53	9,99E-05	1,56E-02
cellular divalent inorganic cation homeostasis (GO:0072503)	485	13	3,65	+	3,56	1,00E-04	1,55E-02
cytosolic calcium ion transport (GO:0060401)	93	6	0,7	+	8,56	1,01E-04	1,55E-02
central nervous system neuron development (GO:0021954)	93	6	0,7	+	8,56	1,01E-04	1,53E-02
adult behavior (GO:0030534)	185	8	1,39	+	5,74	1,06E-04	1,59E-02
regulation of multicellular organismal development (GO:2000026)	2153	33	16,22	+	2,03	1,07E-04	1,60E-02
locomotory behavior (GO:0007626)	241	9	1,82	+	4,96	1,15E-04	1,71E-02
blood vessel morphogenesis (GO:0048514)	425	12	3,2	+	3,75	1,16E-04	1,70E-02
negative regulation of molecular function (GO:0044092)	1029	20	7,75	+	2,58	1,17E-04	1,70E-02
regulation of localization (GO:0032879)	2888	40	21,76	+	1,84	1,22E-04	1,75E-02
sensory perception of chemical stimulus (GO:0007606)	1226	0	9,24	-	< 0.01	1,22E-04	1,74E-02
regulation of MAPK cascade (GO:0043408)	714	16	5,38	+	2,97	1,23E-04	1,74E-02
tube morphogenesis (GO:0035239)	716	16	5,4	+	2,97	1,27E-04	1,78E-02
regulation of anatomical structure size (GO:0090066)	571	14	4,3	+	3,25	1,35E-04	1,87E-02
negative regulation of metabolic process (GO:0009892)	2764	39	20,83	+	1,87	1,38E-04	1,90E-02
metal ion homeostasis (GO:0055065)	647	15	4,88	+	3,08	1,41E-04	1,92E-02
homeostatic process (GO:0042592)	1639	27	12,35	+	2,19	1,46E-04	1,97E-02
positive regulation of cytosolic calcium ion concentration (GO:0007204)	308	10	2,32	+	4,31	1,47E-04	1,97E-02
response to toxic substance (GO:0009636)	310	10	2,34	+	4,28	1,55E-04	2,05E-02
cation transmembrane transport (GO:0098655)	439	12	3,31	+	3,63	1,56E-04	2,05E-02
inorganic ion homeostasis (GO:0098771)	731	16	5,51	+	2,9	1,60E-04	2,09E-02
divalent inorganic cation homeostasis (GO:0072507)	510	13	3,84	+	3,38	1,62E-04	2,10E-02
lipid metabolic process (GO:0006629)	1062	20	8	+	2,5	1,77E-04	2,27E-02
regulation of gliogenesis (GO:0014013)	149	7	1,12	+	6,23	1,77E-04	2,25E-02
ion transmembrane transport (GO:0034220)	587	14	4,42	+	3,17	1,78E-04	2,25E-02
regulation of ion transport (GO:0043269)	738	16	5,56	+	2,88	1,78E-04	2,23E-02
response to axon injury (GO:0048678)	34	4	0,26	+	15,61	1,83E-04	2,27E-02
response to bronchodilator (GO:0097366)	34	4	0,26	+	15,61	1,83E-04	2,26E-02
cellular process (GO:0009987)	14072	129	106,03	+	1,22	2,02E-04	2,48E-02
positive regulation of MAPK cascade (GO:0043410)	522	13	3,93	+	3,31	2,02E-04	2,46E-02
regulation of adenylate cyclase activity (GO:0045761)	35	4	0,26	+	15,17	2,02E-04	2,44E-02
regulation of transport (GO:0051049)	1974	30	14,87	+	2,02	2,06E-04	2,47E-02
regulation of cellular component organization (GO:0051128)	2549	36	19,21	+	1,87	2,18E-04	2,59E-02
neuronal action potential (GO:0019228)	36	4	0,27	+	14,75	2,24E-04	2,64E-02
multicellular organismal signaling (GO:0035637)	110	6	0,83	+	7,24	2,41E-04	2,82E-02
regulation of cellular amine metabolic process (GO:0033238)	38	4	0,29	+	13,97	2,71E-04	3,15E-02
regulation of neural precursor cell proliferation (GO:2000177)	113	6	0,85	+	7,05	2,76E-04	3,19E-02
sensory perception of smell (GO:0007608)	1126	0	8,48	-	< 0.01	2,78E-04	3,18E-02
regulation of kinase activity (GO:0043549)	769	16	5,79	+	2,76	2,80E-04	3,18E-02
regulation of synapse organization (GO:0050807)	273	9	2,06	+	4,38	2,83E-04	3,20E-02
epithelium development (GO:0060429)	1021	19	7,69	+	2,47	2,99E-04	3,36E-02
negative regulation of locomotion (GO:0040013)	339	10	2,55	+	3,91	3,10E-04	3,45E-02
chemotaxis (GO:0006935)	477	12	3,59	+	3,34	3,26E-04	3,60E-02
localization (GO:0051179)	4860	57	36,62	+	1,56	3,33E-04	3,66E-02
cellular component organization or biogenesis (GO:0071840)	5236	60	39,45	+	1,52	3,51E-04	3,83E-02
gas transport (GO:0015669)	16	3	0,12	+	24,88	3,65E-04	3,95E-02
negative regulation of phosphorus metabolic process (GO:0010563)	556	13	4,19	+	3,1	3,65E-04	3,93E-02
negative regulation of phosphate metabolic process (GO:0045936)	556	13	4,19	+	3,1	3,65E-04	3,90E-02
calcium ion transport (GO:0006816)	226	8	1,7	+	4,7	3,92E-04	4,16E-02
cellular response to acid chemical (GO:0071229)	171	7	1,29	+	5,43	3,95E-04	4,16E-02

regulation of phosphorylation (GO:0042325)	1572	25	11,84	+	2,11	4,06E-04	4,26E-02
cation homeostasis (GO:0055080)	718	15	5,41	+	2,77	4,17E-04	4,34E-02
cellular component organization (GO:0016043)	5039	58	37,97	+	1,53	4,18E-04	4,32E-02
negative chemotaxis (GO:0050919)	43	4	0,32	+	12,35	4,19E-04	4,30E-02
chemical synaptic transmission (GO:0007268)	354	10	2,67	+	3,75	4,31E-04	4,40E-02
anterograde trans-synaptic signaling (GO:0098916)	354	10	2,67	+	3,75	4,31E-04	4,37E-02
ion homeostasis (GO:0050801)	801	16	6,04	+	2,65	4,34E-04	4,37E-02
learning or memory (GO:0007611)	293	9	2,21	+	4,08	4,66E-04	4,67E-02
positive regulation of cell differentiation (GO:0045597)	1107	20	8,34	+	2,4	4,70E-04	4,68E-02
regulation of cyclase activity (GO:0031279)	45	4	0,34	+	11,8	4,92E-04	4,86E-02
mechanoreceptor differentiation (GO:0042490)	82	5	0,62	+	8,09	4,97E-04	4,88E-02
negative regulation of cellular metabolic process (GO:0031324)	2469	34	18,6	+	1,83	5,00E-04	4,88E-02
negative regulation of nitrogen compound metabolic process (GO:0051172)	2272	32	17,12	+	1,87	5,02E-04	4,87E-02
gliogenesis (GO:0042063)	235	8	1,77	+	4,52	5,03E-04	4,85E-02
cellular metal ion homeostasis (GO:0006875)	576	13	4,34	+	3	5,06E-04	4,85E-02

Appendix Table S5
Complete list of P-values

Figure		sample number	test	exact p-Value
1	I	n=1 brain; n=3 sections	2way ANOVA (Graphpad)	All significant (***) except: ANTlateral vs. ANTdorsal (n.s.=0.1498); ANTdorsal vs. ANTmedial (n.s.=0.3694); MIDlateral vs. MIDdorsal. (n.s.=0.1764); POSTdorsal vs. POSTmedial (n.s.=0.1498). p-Value shown in Graph for: ANTlateral/MIDlateral; ANTlateral/POSTlateral; MIDlateral/POSTlateral; POSTmedial/POSTlateral; POSTdorsal/POSTlateral: ***= <0.0001
1	M	n=1 brain; n=4 sections	t-test (Excel)	GW14 VZ cells Sulcus/Gyrus: ***=5.68734E-23
1	M'	n=1 brain; n=4 sections	t-test (Excel)	CP cells, pixel intensity; n.s.= 0.0609
1	O	n=1 brain; n=4 convolutions from n=2 sections	t-test (Excel)	GW14 SVZ TBR2+ cells Sulcus/Gyrus: *=0.02037
1	Q	n=1 brain; n=4 sections	t-test (Excel)	GW14 oSVZ cells Sulcus/Gyrus: ***=0.000444351
1	R	n=1 brain; n=4 sections	t-test (Excel)	GW14 Sulcus/Gyrus HOPX+NR2F1+ cells: ***=0.000428
2	B	n=3-6 wells from n=2 independent culture batches	2way ANOVA (Graphpad)	Tukey's multiple comparisons test. p-Value shown in Graph for: WTstep1/KOstep1: n.s.=0.9992; WTstep2/KOstep2: ***= <0,0001; WTstep3/KOstep3: ***= <0,0001; WTstep4/KOstep4: ***= <0,0001; WTstep5/KOstep5: ***= <0,0001; WTstep6/KOstep6: ***= <0,0001; WTstep7/KOstep7: ***= <0,0001; WTstep8/KOstep8: ***= <0,0001; WTstep9/KOstep9: ***= <0,0001.
2	E	n=6 neurospheres from n=2 batches	t-test (Excel)	WT/KO: **=0.006512
3	D	n=3 brains	2way ANOVA (Graphpad)	E12.5 WT/KO: n.s.=0.9999; E13.5 WT/KO: n.s.=0.9999; E14.5 WT/KO: n.s.=0.1032; E15.5 WT/KO: ***=0.0006; E16.5 WT/KO: ***= <0.0001; E17.5 WT/KO: ***= <0.0001; E18.5 WT/KO: ***= <0.0001; P0 WT/KO: ***= 0.0006; P2 WT/KO: n.s.=0.9995; P5 WT/KO: n.s.= >0.9999.
3	G	n=3 brains	2way ANOVA (Graphpad)	E12.5 WT/KO: **=0.0030; E13.5 WT/KO: ***=0.0002; E14.5 WT/KO: *=0.0196; E15.5 WT/KO: n.s.=0.9832; E16.5 WT/KO: n.s.=0.9303; E17.5 WT/KO: **=0.0069; E18.5 WT/KO: n.s.= >0.9999; P0 WT/KO: n.s.= >0.9999.
3	H	n=3 brains	2way ANOVA (Graphpad)	E12.5 WT/KO: n.s.=0.8271; E13.5 WT/KO: n.s.= >0.9999; E14.5 WT/KO: *=0.0485; E15.5 WT/KO: n.s.=0.3042; E16.5 WT/KO: **=0.0055; E17.5 WT/KO: ***=0.0002; E18.5 WT/KO: n.s.=0.1945; P0 WT/KO: n.s.=0.9958; P5 WT/KO: n.s.= >0.9999.
3	I	n=3 brains	2way ANOVA (Graphpad)	E12.5 WT/KO: n.s.= >0.9999; E13.5 WT/KO: n.s.= >0.9999; E14.5 WT/KO: n.s.= 0.9966; E15.5 WT/KO: n.s.=0.0770; E16.5 WT/KO: n.s.= 0.8775; E17.5 WT/KO: ***= <0.0001; E18.5 WT/KO: ***= <0.0001; P0 WT/KO: ***= <0.0001.
3	L	n=3 brains	2way ANOVA (Graphpad)	p-Value shown in Graph for: WT/KO level11: n.s.= >0.9999; WT/KO level12: n.s.= >0.9885; WT/KO level13: **=0.0014; WT/KO level14: ***= <0.0001; WT/KO level15: ***= <0.0001; WT/KO level16: n.s.=0.6345; WT/KO level17: n.s.= >0.9999; WT/KO level18: n.s.= >0.9999. All not showed p-Values: n.s.= >0.9999.
3	P	n=3 electroporated brains	2way ANOVA (Graphpad)	WTsox2GFP/KOsox2GFP: **= 0.0038; WTtis21RFP/KOtis21RFP: n.s.=0.9999; WTdouble/KOdouble: **=0,0043.
3	Q-R'	n=4-6 sections from n=2 brains	t-test (Excel)	Short term differentiation assay, EdU injection at E12.5, analysis at E13.5. WT/KO: *=0.03967.
4	A	n=3-4 brains	2way ANOVA (Graphpad)	E12.5 WT/KO: n.s.=0.7661; E13.5 WT/KO: n.s.=0.3251; E14.5 WT/KO: **=0.0038; E15.5 WT/KO: n.s.=0.6937; E16.5 WT/KO: ***=0.0001; E17.5 WT/KO: **=0.0052; E18.5 WT/KO: n.s.=0.0994; P0 WT/KO: n.s.=0.4115; P2 WT/KO: n.s.=0.1319; P5 WT/KO: n.s.= >0.9999.
4	B	n=3-4 brains	2way ANOVA (Graphpad)	E12.5 WT/KO: n.s.=0.9805; E13.5 WT/KO: n.s.=0.8332; E14.5 WT/KO: ***=0.0005; E15.5 WT/KO: n.s.=0.7251; E16.5 WT/KO: *=0.0154; E17.5 WT/KO: n.s.= >0.9999; E18.5 WT/KO: n.s.= >0.9999; P0 WT/KO: n.s.= >0.9999; P2 WT/KO: n.s.= >0.9999; P5 WT/KO: n.s.= >0.9999.
4	C	n=3-4 brains	2way ANOVA (Graphpad)	E12.5 WT/KO: n.s.= >0.9999; E13.5 WT/KO: n.s.=0.9963; E14.5 WT/KO: ***= <0.0001; E15.5 WT/KO: n.s.= >0.9999; E16.5 WT/KO: n.s.=0.1579; E17.5 WT/KO: n.s.=0.2434; E18.5 WT/KO: n.s.=0.1191; P0 WT/KO: n.s.=0.9525; P2 WT/KO: n.s.= >0.9999; P5 WT/KO: n.s.= >0.9999.
4	D	n=3-4 brains	2way ANOVA (Graphpad)	E12.5 WT/KO: n.s.= >0.9999; E13.5 WT/KO: n.s.= >0.9999; E14.5 WT/KO: n.s.= >0.9999; E15.5 WT/KO: n.s.= >0.9999; E16.5 WT/KO: n.s.=0.9996; E17.5 WT/KO: n.s.=0.1510; E18.5 WT/KO: **=0.0019; P0 WT/KO: ***= <0.0001; P2 WT/KO: *=0.0164; P5 WT/KO: n.s.=0.9973.
4	E	n=3-4 brains	2way ANOVA (Graphpad)	E10.5 WT/KO: n.s.= >0.9999; E12.5 WT/KO: *=0.0454; E13.5 WT/KO: n.s.=0.3247; E14.5 WT/KO: ***= <0.0001; E15.5 WT/KO: ***= <0.0001; E16.5 WT/KO: n.s.=0.0736.
4	H	n=2 brains	2way ANOVA (Graphpad)	EdU2h WT/KO: **=0.0068; EdU4h WT/KO: **=0.0011; EdU6h WT/KO: **=0.0057; EdU8h WT/KO: ***= <0.0001.
4	K	n=2 brains	t-test (Excel)	G2 phase cell percentage WT/KO: *=0.01811.
4	L	n=3 brains	2way ANOVA (Graphpad)	TimeG1 WT/KO: ***= <0.0001; TimeS WT/KO: n.s.=0.3431; TimeG2 WT/KO: n.s.= >0.9999; TimeM WT/KO: n.s.= >0.9999.
5	C	n=3 brains	2way ANOVA (Graphpad)	Pax6 and Nr2f1 pixel intensity levels (green and red channels, respectively) were analysed separately. Pax6 E12.5 WT/KO: *=0.0325; Pax6 E14.5 WT/KO: *=0.0246; Nr2f1 E12.5 WT/KO: ***= <0.0001; Nr2f1 E12.5 WT/KO: ***= <0.0001.
5	D	n=3 brains	2way ANOVA (Graphpad)	Pax6 WT/KO: **=0.0010; Nr2f1 WT/KO: **=0.0035.
5	H	n=2 electroporated brains	2way ANOVA (Graphpad)	Pax6 average intensity pCIG2-GFP/pCIG2-Nr2f1-GFP: ***= <0.0001; Nr2f1 average intensity pCIG2-GFP/pCIG2-Nr2f1-GFP: ***= <0.0001.

5	I	n=3 brains	2way ANOVA (Graphpad)	Fisher's LSD test (no correction for multiple comparisons). Nr2f1 E12.5 WT/KO: ***=0.0002; CycB1 E12.5 WT/KO: n.s.=0.3262; CycB2 E12.5 WT/KO: n.s.=0.8574; CycD1 E12.5 WT/KO: *.=0.0281; CycD2 E12.5 WT/KO: n.s.=0.4124; CycE E12.5 WT/KO: n.s.=0.0550; Cdc2 E12.5 WT/KO: n.s.=0.2745; CycD3 E12.5 WT/KO: n.s.=0.4045; CycA2 E12.5 WT/KO: n.s.=0.5975; CycD3 E12.5 WT/KO: n.s.=0.4045; cmyc E12.5 WT/KO: n.s.=0.0772; MycN E12.5 WT/KO: n.s.=0.6292; Cdk2 E12.5 WT/KO: n.s.=0.5653; Cdk4 E12.5 WT/KO: n.s.=0.5649; P15 E12.5 WT/KO: n.s.=0.4771; P18 E12.5 WT/KO: n.s.=0.1316; P19 E12.5 WT/KO: n.s.=0.4269; P21 E12.5 WT/KO: *=0.0390; P27 E12.5 WT/KO: n.s.=0.8973; P57 E12.5 WT/KO: n.s.=0.9279; Dct E12.5 WT/KO: ***= <0.0001.
5	L	n=2 electroporated brains	t-test (Excel)	P21 cells PX458/αNr2f1 PX458: *=0.0460.
5	N	n=3 dissected cortices per genotype	see Methods	RNA-Seq analysis, see Methods
5	O	n=3 dissected cortices per genotype	see Methods	RNA-Seq analysis, see Methods
5	P	n=3 dissected cortices per genotype	see Methods	RNA-Seq analysis, see Methods
6	A	n=3 brains	2way ANOVA (Graphpad)	WT vs. Pax6 het: **=0.0017; WT vs. Pax6 ko: ***= <0.0001; WT vs. Nr2f1 ko: *=0.0166; WT vs. Nr2f1 ko Pax6 het: n.s.= >0.9999; Pax6 het vs.Pax6 ko: n.s.=0.4309; Pax6 het vs. Nr2f1 ko: ***= <0.0001; Pax6 het vs. Nr2f1 ko Pax6 het: **=0.0017; Pax6 ko vs. Nr2f1 ko: ***= <0.0001; Pax6 ko vs. Nr2f1 ko Pax6 het: ***= <0.0001; Nr2f1 ko vs. Nr2f1 ko Pax6 het: *=0.0159.
6	C	n=3-6 wells from n=2 independent culture batches	2way ANOVA (Graphpad)	WTstep1/KOstep1: n.s.=0.9999; WTstep2/KOstep2: ***= <0,0001; WTstep3/KOstep3: ***= <0,0001; WTstep4/KOstep4: ***= <0,0001; WTstep5/KOstep5: ***= <0,0001; WTstep6/KOstep6: ***= <0,0001; WTstep7/KOstep7: ***= <0,0001; WTstep8/KOstep8: ***= <0,0001; WTstep9/KOstep9: ***= <0,0001; WTstep10/KOstep10: ***= <0,0001. WTstep1/NkoPhet step1: n.s.=0.9999; WTstep2/NkoPhet step2: *=0,0288; WTstep3/NkoPhet step3: **=0,0039; WTstep4/NkoPhet step4: n.s.=0.9999; WTstep5/NkoPhet step5: n.s.=0.9989; WTstep6/NkoPhet step6: n.s.=0.1902; WTstep7/NkoPhet step7: n.s.=0.9202; WTstep8/NkoPhet step8: n.s.= >0.9999; WTstep9/NkoPhet step9: n.s.= >0.9999; WTstep10/NkoPhet step10: n.s.= >0.9999. KOstep1/NkoPhet step1: n.s.= >0.9999; KOstep2/NkoPhet step2: ***= <0,0001; KOstep3/NkoPhet step3: **=0,0012; KOstep4/NkoPhet step4: ***= <0,0001; KOstep5/NkoPhet step5: ***= <0,0001; KOstep6/NkoPhet step6: ***= <0,0001; KOstep7/NkoPhet step7: ***= <0,0001; KOstep8/NkoPhet step8: ***= <0,0001; KOstep9/NkoPhet step9: ***= <0,0001; KOstep10/NkoPhet step10: ***= <0,0001.
6	E	n=3-6 wells from n=2 independent culture batches	2way ANOVA (Graphpad)	WT P-P/KO P-P: ***= 0.0001; WT P-P/NkoPhet P-P: n.s.=0.9984; NkoPhet P-P/KO P-P: ***= 0.0002; WT P-N/KO P-N: n.s.= 0.0838; WT P-N/NkoPhet P-N: n.s.=0.9984; NkoPhet P-N/KO P-N: n.s.= 0.1884; WT N-N/KO N-N: **= 0.0033; WT N-N/NkoPhet N-N: n.s.= >0.9999; NkoPhet N-N/KO N-N: **= 0.0033.
6	I	n=3 brains	2way ANOVA (Graphpad)	Pax6 WT/Nko: ***= <0.0001; Pax6 WT/NkoPhet: n.s.=0.6694; Pax6 Nko/NkoPhet: ***= <0.0001; Nr2f1 WT/Nko: ***= <0.0001; Nr2f1 WT/NkoPhet: ***= <0.0001; Nr2f1 Nko/NkoPhet: ***= <0.0001.
6	J	n=6 sections from n=2 brains	2way ANOVA (Graphpad)	T cell cycle WT/Nko: **=0.0012; WT/NkoPhet: n.s.=0.1870; WT/Phet: n.s.=0.9808; Nko/NkoPhet: n.s.=0.4527; Phet/Nko: **=0.0014; Phet/NkoPhet: n.s.=0.1332.
6	K	n=2-3 brains per genotype	2way ANOVA (Graphpad)	Vertical division plane: WT/Nko: ***=0.0006; WT/NkoPhet: n.s.= >0.9999; Nko/NkoPhet: ***=0.0005; Oblique division plane: WT/Nko: n.s.=0.1557; WT/NkoPhet: n.s.=0.9985; Nko/NkoPhet: *=0.0468; Horizontal division plane: WT/Nko: n.s.=0.1557; WT/NkoPhet: n.s.=0.9996; Nko/NkoPhet: n.s.=0.3663;
7	C	n=4-5 organoids from 2 batches	2way ANOVA (Graphpad)	NR2F1 negative cells: day30-organoids/day40-organoids: n.s.=0.9999; day30-organoids/day70-organoids: n.s.=0.5521; day40-organoids/day70-organoids: n.s.=0.8452; NR2F1 positive cells, low intensity: day30-organoids/day40-organoids: n.s.=0.9997; day30-organoids/day70-organoids: n.s.=0.0994; day40-organoids/day70-organoids: n.s.=0.2944; NR2F1 positive cells, high intensity: day30-organoids/day40-organoids: n.s.=0.9791; day30-organoids/day70-organoids: ***=0.0004; day40-organoids/day70-organoids: **=0.0087;
7	G	n=4-5 organoids from 2 batches	2way ANOVA (Graphpad)	PAX6 pixel intensity NR2F1high Region/NR2F1low Region: ***=0.0002; NR2F1 pixel intensity NR2F1high Region/NR2F1low Region: ***= <0.0001.
7	J	n=6-10 organoids from 2 batches	2way ANOVA (Graphpad)	GFP+NR2F1- CTRL/aNR2F1: *=0.0136; GFP+NR2F1low CTRL/aNR2F1: n.s.=0.9974; GFP+NR2F1high CTRL/aNR2F1: *=0.0345.
7	M	n=6-10 organoids from 2 batches	2way ANOVA (Graphpad)	GFP+PAX6+ neural progenitors CTRL/aNR2F1: *=0.0295; GFP+TUJ1+ neurons CTRL/aNR2F1: *=0.0294.
EV1	J	n=1 brain; n=4 sections	2way ANOVA (Graphpad)	Anterior/Middle: **=0.0017; Anterior/Posterior and Middle/Posterior: ***=<0.0001
EV1	L	n=1 brain; n=4 sections	t-test (Excel)	SOX2+ VZ cells per 100 μm Sulcus/Gyrus: ***=0.000456
EV1	L'	n=1 brain; n=4 sections	t-test (Excel)	TBR2+ SVZ cells per 100 μm Sulcus/Gyrus: ***=0.000312
EV1	L''	n=1 brain; n=4 sections	t-test (Excel)	SOX2+ oSVZ cells per 100 μm Sulcus/Gyrus: **=0.003636
EV2	G	n=4-6 sections from n=2 brains	2way ANOVA (Graphpad)	Sox2+Tbr2-Ki67+ in VZ-SVZ, WT/KO: **=0.0080; Sox2+Tbr2-Ki67+ in outerSVZ, WT/KO: ***= <0.0001; Sox2-Tbr2-Ki67+ in outerSVZ, WT/KO: *=0.0185. All other WT/KO comparisons: n.s.=0.9999.
EV2	K	n=4-6 sections from n=2 brains	2way ANOVA (Graphpad)	PVim+Tbr2-Pax6+ in VZ-SVZ, WT/KO: **=0.0031; PVim+Tbr2-Pax6+ in outerSVZ, WT/KO: n.s.=0.6352; PVim+Tbr2+Pax6+ in outerSVZ, WT/KO: ***=0.0002. All other WT/KO comparisons: n.s.=0.9999.
EV3	D	n=2-3 brains	2way ANOVA (Graphpad)	apicall RGC cells (Pax6+ in VZ). WT/KO: ***= <0.0001; WT/HET: n.s.=0.0513; HET/KO: n.s.=0.0522.

EV3	E	n=2-3 brains	2way ANOVA (Graphpad)	IP cells (Tbr2+ in SVZ). WT/KO: ***= <0.0001; WT/HET: **=0.0051; HET/KO: *=0.0198.
EV3	F	n=2-3 brains	2way ANOVA (Graphpad)	basal RGC cells (Pax6+ in outer SVZ and IZ). WT/KO: *=0.0312; WT/HET: n.s.=0.1200; HET/KO: n.s.=0.7774.
EV3	J	n=4-6 sections from n=2 brains	2way ANOVA (Graphpad)	Short term differentiation assay, EdU injection at E12.5, analysis at E13.5. WT/KO: *=0.0383; WT/HET: n.s.=0.3220; HET/KO: n.s.=0.4374.
EV4	A	n=4-6 sections from n=2 brains	2way ANOVA (Graphpad)	Short term differentiation assay (analysis 24 hours after EdU injection). E13.5 (EdU at E12.5) WT/KO: *=0.0394; E16.5 (EdU at E15.5) WT/KO: ***=0.0004; P0 (EdU at E18.5) WT/KO: ***=0.0002.
EV4	D	n=4-6 sections from n=2 brains	t-test (Excel)	Short term differentiation assay (analysis 24 hours after EdU injection). Normalised on total EdU+ cells. Analyzed separately by t-student test. E13.5 (EdU at E12.5) WT/KO: *=0.04537; E16.5 (EdU at E15.5) WT/KO: n.s.=0.71403; P0 (EdU at E18.5) WT/KO: n.s.=0.424127.
EV4	E	n=4-6 sections from n=2 brains	2way ANOVA (Graphpad)	P0EdU11.5 WT/KO: n.s.= >0.9999; P0EdU12.5 WT/KO: **=0.0012; P0EdU13.5 WT/KO: **=0.0042; P0EdU14.5 WT/KO: ***= <0.0001; P0EdU15.5 WT/KO: ***= <0.0001; P0EdU16.5 WT/KO: n.s.=0.2996.
EV4	F''	n=4-6 sections from n=2 brains	2way ANOVA (Graphpad)	Analysis at P0, EdU injected at E12.5: EdU+ cells WT/KO: *=0.0345; EdU+ Tbr1+ cells WT/KO: **=0.0012; EdU+ Ctip2+ cells WT/KO: n.s.=0.9998; EdU+ Tbr1+ Ctip2+ cells WT/KO: n.s.=0.9947.
EV4	G''	n=4-6 sections from n=2 brains	2way ANOVA (Graphpad)	Analysis at P0, EdU injected at E15.5: EdU+ cells WT/KO: n.s.=0.4289; EdU+ Cux1+ cells WT/KO: ***= <0.0001.
EV4	J	n=4-6 sections from n=2 brains	2way ANOVA (Graphpad)	Analysis at P8, EdU injected at E17.5: EdU+ cells WT/KO: n.s.=0.9979; EdU+ GFAP+ astrocytes WT/KO: *=0.0150; EdU+ NeuN+ neurons WT/KO: ***= <0.0001.
EV4	L''	n=2 brains per genotype and age	2way ANOVA (Graphpad)	Tuj1+ cortical plate thickness: E17.5 WT/KO: ***= <0.0001; P0 WT/KO: ***= <0.0001.
EV4	M	n=4-6 sections from n=2 brains	2way ANOVA (Graphpad)	Tuj1+ neurons per box: E12.5 WT/KO: n.s.= >0.9999; E13.5 WT/KO: n.s.= >0.9999; E14.5 WT/KO: n.s.=0.9956; E15.5 WT/KO: n.s.= >0.9999; E16.5 WT/KO: n.s.=0.9970; E17.5 WT/KO: *= 0.0335; E18.5 WT/KO: ***= <0.0001; P0 WT/KO: ***= <0.0001; P2 WT/KO: **=0.0019; P5 WT/KO: ***=0.0003.
EV4	Q	n=4-6 sections from n=2 brains	2way ANOVA (Graphpad)	P8 neocortical neurons per transversal box: Satb2+ WT/KO: *=0.0488; Ctip2+ WT/KO: n.s.= >0.9999; Tbr1+ WT/KO: ***= <0.0001.
EV5	C	n=3 brains	2way ANOVA (Graphpad)	Anterior VZ WT/KO: n.s.= >0.9999; Anterior SVZ WT/KO: n.s.=0.9997; Anterior oSVZ WT/KO: n.s.=0.9786; Middle VZ WT/KO: n.s.=0.9966; Middle SVZ WT/KO: ***=0.0002; Middle oSVZ WT/KO: n.s.=0.9784; Posterior VZ WT/KO: **=0.0039; Posterior SVZ WT/KO: n.s.=0.6270; Posterior oSVZ WT/KO: n.s.=0.5629.
EV5	F	n=3-4 brains	2way ANOVA (Graphpad)	E12.5 cell cycle duration along A-P axis: Anterior WT/KO: n.s.=0.1033; Medial WT/KO: n.s.=0.9921; Posterior WT/KO: **=0.0032.
EV5	F'	n=3-4 brains	2way ANOVA (Graphpad)	E14.5 cell cycle duration along A-P axis: Anterior WT/KO: n.s.=0.4579; Medial WT/KO: *=0.0336; Posterior WT/KO: **=0.0014.
EV5	G	n=2-3 brains	2way ANOVA (Graphpad)	E14.5 cell cycle duration in WT, HET and KO lateral pallia: WT/HET: n.s.=0.0855; HET/KO: n.s.=0.6339; WT/KO: **=0.0028.

AF1	C	n=3 cell culture wells (total 7000-9000 cells)	2way ANOVA (Graphpad)	WT/HET: n.s.=0.7372; WT/KO: **=0.0029; HET/KO: *=0.0134
AF2	D	n=2 pools of n=3 brains each; 10.000 total events (cells) per acquisition	See AF2 D'-D'''	See AF2 D'-D'''
AF2	D'	n=2 pools of n=3 brains each; 10.000 total events (cells) per acquisition	2way ANOVA (Graphpad)	S phase cell percentage (over total events): Anterior cortex WT/KO: n.s.=0.2875; Posterior cortex WT/KO: *=0.0167.
AF2	D''	n=2 pools of n=3 brains each; 10.000 total events (cells) per acquisition	2way ANOVA (Graphpad)	G2 phase cell percentage (over total events): Anterior cortex WT/KO: n.s.=0.3779; Posterior cortex WT/KO: n.s.=0.0606.
AF2	D'''	n=2 pools of n=3 brains each; 10.000 total events (cells) per acquisition	2way ANOVA (Graphpad)	M phase cell percentage (PH3+% over total events): Anterior cortex WT/KO: *=0.0200; Posterior cortex WT/KO: ***=0.0007.
AF2	G	n=1 pool of n=3 brains each; 10.000 total events (cells) per acquisition	p-Value not tested	///
AF3	E	n=4-6 sections from n=2 brains	2way ANOVA (Graphpad)	Ki67/Tuj1 ratio in E14.5 WT brains. LP/DP: ***= <0.0001; LP/MP: ***= <0.0001; DP/MP: ***=0.0004.
AF3	F	n=4-6 sections from n=2 brains	2way ANOVA (Graphpad)	LP (lateral pallium) WT/KO: *=0.0108; DP (dorsal pallium) WT/KO: n.s.=0.1436; MP (medial pallium) WT/KO: n.s.=0.9996.
AF3	K	n=3 brains	2way ANOVA (Graphpad)	LP (lateral pallium) WT/KO: *=0.0115; DP (dorsal pallium) WT/KO: n.s.=0.9926; MP (medial pallium) WT/KO: n.s.=0.9944.
AF3	O	n=3 brains	2way ANOVA (Graphpad)	Cell cycle time (EdUBrdU protocol): LP (lateral pallium) WT/KO: ***=0.0002; DP (dorsal pallium) WT/KO:*=0.0469; MP (medial pallium) WT/KO: n.s.= >0.9999.
AF4	D	n=3 brains	2way ANOVA (Graphpad)	Nr2f1 pixel intensity levels (red channel). E13.5 WT/KO: ***= <0.0001; E13.5 WT/HET: ***= <0.0001; E13.5 HET/KO: ***= <0.0001.
AF4	E	n=3 brains	2way ANOVA (Graphpad)	Pax6 pixel intensity levels (green channel). E13.5 WT/KO: ***= <0.0001; E13.5 WT/HET: *=0.0418; E13.5 HET/KO: n.s.=0.0800.
AF5	C	n=2 electroporated brains	2way ANOVA (Graphpad)	GFP+NR2F1- CTRL/aNR2F1: *=0.0135; GFP+NR2F1low CTRL/aNR2F1: n.s.=0.9883; GFP+NR2F1high CTRL/aNR2F1: *=0.0338.
AF6	/	n=3 dissected cortices per genotype	see Methods	RNA-Seq analysis, see Methods

Appendix Table S6

List of real time RT-PCR primers

Pax6	Forward	CACACGCCCTGGTTGG
	Reverse	TGGGTCCTCTCAAACTCTTTTCT
Nr2f1	Forward	TCCCATCGAAACTCTCATCC
	Reverse	AGTGGGCTGCTCTTGTTCC
CycB1	Forward	TGCATTTTGCTCCTTCTCAA
	Reverse	CAGGAAGCAGGGAGTCTTCA
CycB2	Forward	CAACCGTACCAAGTTCATCG
	Reverse	GAGGGATCGTGCTGATCTTC
CycD1	Forward	GAGATTGTGCCATCCATGC
	Reverse	CTCCTCTTCGCACTTCTGCT
CycD2	Forward	CACCGACAACTCTGTGAAGC
	Reverse	TCCACTTCAGCTTACCCAACA
CycE	Forward	CTGAGAGATGAGCACTTTCTGC
	Reverse	GAGCTTATAGACTTCGCACACCT
Cdc2	Forward	CTTCGACATCCAAATATAGTCAGC
	Reverse	CCATGGACAGGAACTCAAAGA
CycD3	Forward	GGCATACTGGATGCTGGAG
	Reverse	CCAGGTAGTTCATAGCCAGAGG
CycA2	Forward	CTTGGCTGCACCAACAGTAA
	Reverse	CAAACTCAGTTCTCCCAAAAACA
cMYC	Forward	CCTAGTGCTGCATGAGGAGA
	Reverse	TCCACAGACACCACATCAATTT
mycN	Forward	TGTGTCTGTTCCAGCTACTGC
	Reverse	CTTCCTCCTCGTCATCCTCA
CDK2	Forward	TGCATCTTTGCTGAAATGGT
	Reverse	AGATCCGGAAGAGTTGGTCA
CDK4	Forward	TCAGTGGTGCCAGAGATGG
	Reverse	GGAAGGCAGAGATTCGCTTA
P15	Forward	AATAACTTCCTACGCATTTTCTGC
	Reverse	CCCTTGGCTTCAAGGTGAG
P18	Forward	AAATGGATTTGGGAGAACTGC
	Reverse	AAATTGGGATTAGCACCTCTGA
P19	Forward	AATGTGACCCAAGGCCACT
	Reverse	TTTCCTCTTTTGTTGACAAGTAACC
P21	Forward	AACATCTCAGGGCCGAAA
	Reverse	TGCGCTTGGAGTGATAGAAA
P27	Forward	GAGCAGTGTCCAGGGATGAG
	Reverse	TCTGTTCTGTTGGCCCTTTT
P57	Forward	CGCAAACGTCTGAGATGAGT
	Reverse	CCCAGAGTTCTTCCATCGTC
MCM2	Forward	CAACTTTGTACTGGGGCTTT
	Reverse	CTGGATGCGGATACGTTGGT
Dct	Forward	CATGGTCCCCTTCTTCCCAC
	Reverse	GAAAAGCCAGCAACCCCAAG
GADPH	Forward	CATGGCCTTCCGTGTTCCTA
	Reverse	TGCCTGCTTCACCACCTTCT
