# Supplementary material for: NR2F1 regulates regional progenitor dynamics in the mouse neocortex and cortical gyrification in BBSOAS patients
Source: EMBO J. 2020 Jun 2;39(13):e104163. doi: 10.15252/embj.2019104163 (PMC7327499; doi:10.15252/embj.2019104163)
Supplement: Supplementary file 2 — Expanded View Figures PDF [file EMBJ-39-e104163-s002.pdf]

Expanded View Figures

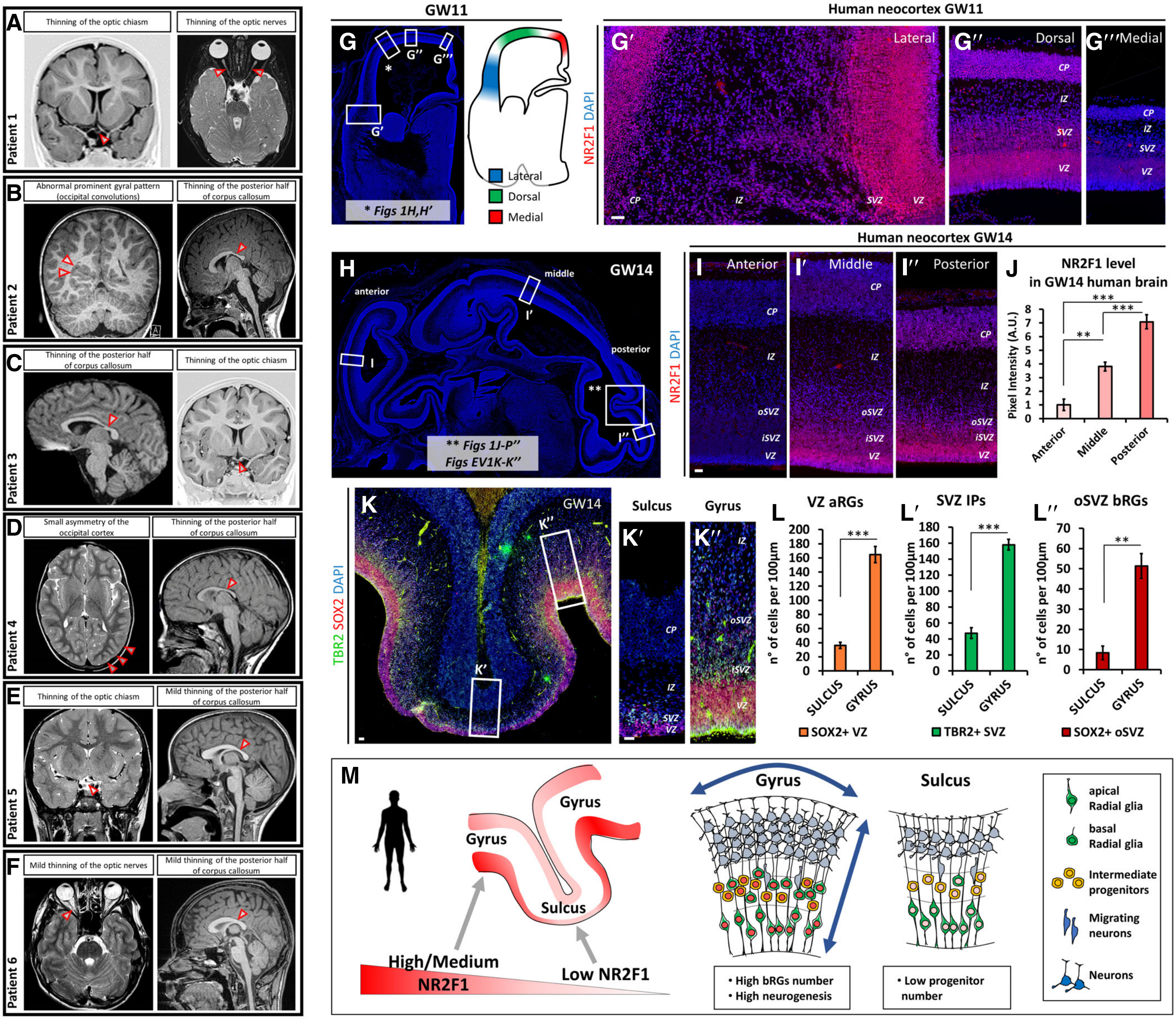

Figure EV1.

 © 2020 The Authors

Figure EV1. MRI features of six novel BBSOAS patients and NR2F1 expression along the L-M and A-P axes.

A Two-year-old female patient with thinning of the optic chiasm (left image) and optic nerves (right image).
B Four-year-old male patient showing prominent occipital convolutions (left) together with thinning of the posterior half of corpus callosum (right).
C Six-year-old female patient with thinning of the posterior half of corpus callosum (left) and of the optic chiasm (right).
D Three-year-old female patient with small asymmetry of the occipital cortex (left), thinning of the posterior half of corpus callosum (right), and normal thickness of optic nerves (not shown).
E Seven-year-old female patient with thinning of the optic chiasm (left) and mild thinning of posterior corpus callosum (right).
F Twelve-year-old male patient showing mild thinning of the optic nerves (left) and of the posterior half of corpus callosum (right).
G–G‴ Low-magnification DAPI staining and schematic representation of a GW11 coronal section (G) displaying the position of high-magnification views in (G′-G‴) and in Fig 1H and H′. Colors in (G) display lateral, dorsal, and medial areas taken for NR2F1 pixel intensity measurements at GW11 (see Fig 1I). Representative images of NR2F1 (red) immunofluorescence (IF) at different levels along the latero-medial (L-M) extent of the cortex, as shown in (G′-G″).
H Low-magnification DAPI staining of a GW14 sagittal section displaying the position of high-magnification images showed in (I-I″, K-K″) and in Fig 1J–L′, N, N′ and P–P″.
I, J NR2F1 (red) IF at GW14 depicting increasing expression levels in the posterior-most cortex (I″), as quantified in (J). $n \geq 4$ sections from $n = 1$ fetal brain.
K–L″ TBR2 (green) and SOX2 (red) IF of a GW14 primary convolution (detail taken from H). The average number of different NP classes in the fissure (K′) and convoluted region (K″) is quantified in (L-L″). The convoluted region is populated by a higher number of both apical and basal progenitors. $n \geq 4$ sections from $n = 1$ fetal brain.
M Schematic representation of human NR2F1 expression levels correlated with distinct cytoarchitectures along cortical convolutions. Expression of NR2F1 displays local modules of low versus medium/high expression levels in sulci and gyri, respectively. Medium/high NR2F1 expression in gyri is associated with high basal RG numbers and high neurogenic activity promoting radial and tangential expansion, whereas low expression is correlated with a small progenitor pool and low neurogenic potential.

Data information: Nuclei (blue) were stained with DAPI. In (J, L-L″), the number of positive cells or the pixel intensity was quantified in 100 μm-width boxes, randomly placed across the cortex in exemplificative regions, as shown in (H). Data are represented as means ± SEM. Two-way ANOVA (J) and Student's *t*-test (L-L″) (**$P < 0.01$, ***$P < 0.001$). Scale bars: 50 μm. In (A-F), arrowheads highlight the morphological features described above the MRI for each patient. CP: cortical plate; IZ: intermediate zone; iSVZ/oSVZ: inner/outer subventricular zone; VZ: ventricular zone.

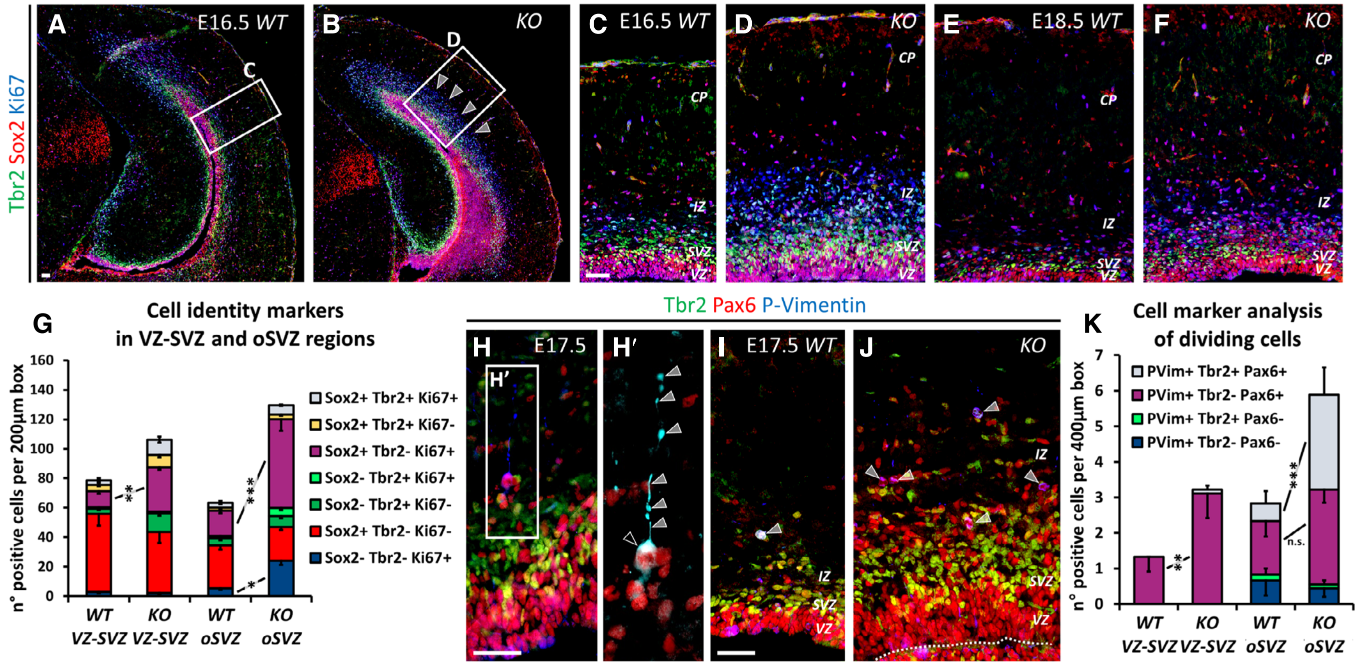

Figure EV2. Molecular identity of basal (b) RGs in Nr2f1 mutant cortices.

A–F Tbr2 (green), Sox2 (red), and Ki67 (blue) triple IF of *WT* (A, C, E) and *KO* (B, D, F) neocortices at E16.5 (A–D) or at E18.5 (E, F) showing abundant NPs in mutant brains. bRGs located out of the Tbr2$^+$ SVZ layer are very abundant in the mutant lateral pallium (LP; arrowheads in B).

G Average number of NPs with distinct molecular profiles in the LPs of E18.5 control and mutant embryos. Cells were evaluated separately in the VZ-SVZ (left) and in the IZ basal to the Tbr2$^+$ SVZ layer (hereinafter oSVZ; right). A high number of Ki67$^+$Sox2$^+$Tbr2$^-$ NPs are found in the *KO* oSVZ, consistent with bRG identity. $n \geq 4$ sections from $n = 2$ brains.

H–J Tbr2 (green), Pax6 (red), and Phospho-Vimentin (blue) triple IF of E17.5 *WT* (H, I) and *KO* (J) LPs. The typical morphology of a bRG cell is shown in (H) and magnified in (H′); gray arrowheads point to the apical process, and empty arrowhead highlights the Pax6$^+$ bRG soma. Note a high number of P-Vim$^+$ cells in the oSVZ of *KO* embryos (J; arrowheads point to P-Vim$^+$ cells).

K Average number of dividing (P-Vim$^+$) cells in the LPs of E18.5 *WT* and *KO* embryos. Cells were evaluated separately in the VZ-SVZ (left) and oSVZ (right). Mutant brains have a higher number of P-Vim$^+$ cells expressing Pax6 or both Pax6 and Tbr2, and located in the VZ-SVZ or in the oSVZ layer, respectively. $n \geq 4$ sections from $n = 2$ brains.

Data information: In (G, K), the number of positive cells was quantified in 200 µm- or 400 µm-width boxes, as indicated, randomly placed across the LP. In graphs, data are represented as means ± SEM. Two-way ANOVA (**$P < 0.01$, ***$P < 0.001$). Scale bars: 50 µm. CP: cortical plate; IZ: intermediate zone; SVZ: subventricular zone; VZ: ventricular zone.

Figure EV3. Nr2f1 dose-dependent control of progenitor amplification and differentiation *in vivo*.

A–C Tbr2 (green; IPs) and Pax6 (red; radial glia cells) IF of E17.5 lateral pallia of *WT* (A, A′), *HET* (B) and *KO* (C) brains.

D–F Quantification of different classes of cortical progenitors in distinct genotypes as indicated; apical radial glia cells (aRGs) were quantified as Pax6$^+$ NPs in VZ (D), basal intermediate progenitors (IPs) as Tbr2$^+$ cells in SVZ (E), and basal RGs as Pax6$^+$ cells in outer SVZ (F). $n \geq 2$ brains.

G–I′ Ki67 (green; progenitors) and EdU (red) IF of E13.5 *WT* (G-G″), *HET* (H, H′), and *KO* (I, I′) embryos, injected with EdU at E12.5. Differentiating cells (EdU$^+$Ki67$^-$) are located in the intermediate zone (IZ) or in the cortical plate (CP).

J Graph showing the average number of EdU$^+$Ki67$^-$ differentiating cells in *WT* (blue column), *HET* (gray column), and *KO* (orange column) cortices quantified 24 h after EdU injection. Delayed at E13.5 in *KO* brains, neurogenesis shows a decreasing trend also in *HET* brains. The number of EdU$^+$Ki67$^-$ differentiating cells was re-normalized on total number of Ki67$^+$ cells, to factor-in the increased size of progenitor pool in mutant brains compared to *WT*. $n \geq 4$ sections from $n = 2$ brains.

Data information: Nuclei (blue) were stained with DAPI. The number of positive cells was quantified in 100 µm-width boxes, randomly placed across the LP. Data are represented as means ± SEM. Two-way ANOVA (n.s.=not significant, $P > 0.05$; *$P < 0.05$, **$P < 0.01$, ***$P < 0.001$). Scale bars: 50 µm. CP: cortical plate; IZ: intermediate zone; SVZ: subventricular zone; VZ: ventricular zone.

 © 2020 The Authors

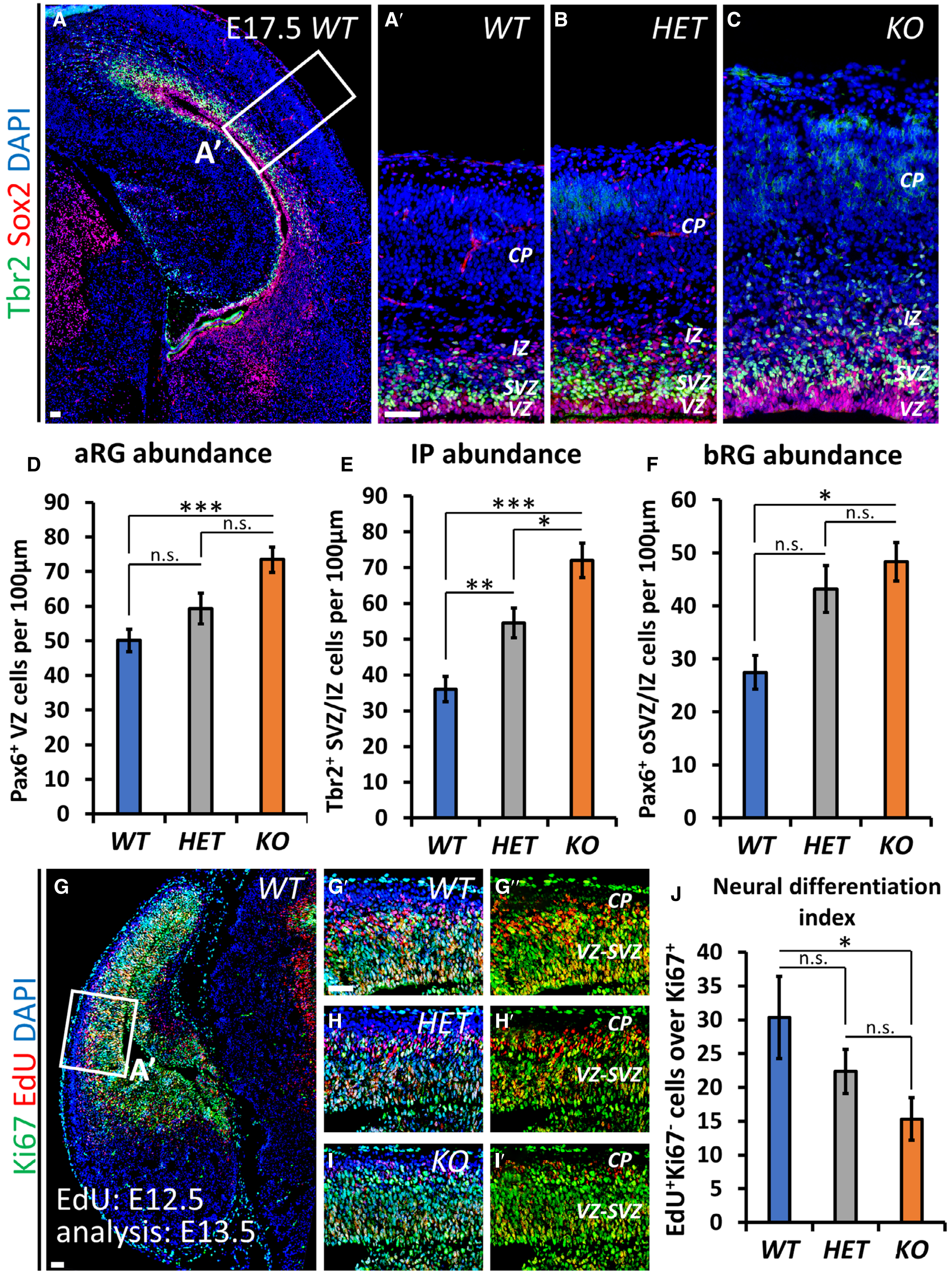
Tbr2 Sox2 DAPI
A E17.5 WT
A′
A′ WT
B HET
C KO
CP
IZ
SVZ
VZ
D aRG abundance
Pax6+ VZ cells per 100µm
E IP abundance
Tbr2+ SVZ/IZ cells per 100µm
F bRG abundance
Pax6+ oSVZ/IZ cells per 100µm
***
**
*
n.s.
WT HET KO
Ki67 EdU DAPI
G WT
EdU: E12.5
analysis: E13.5
G′ WT
G″
H HET
H′
I KO
I′
VZ-SVZ
J Neural differentiation index
EdU+Ki67- cells over Ki67+

Figure EV3.

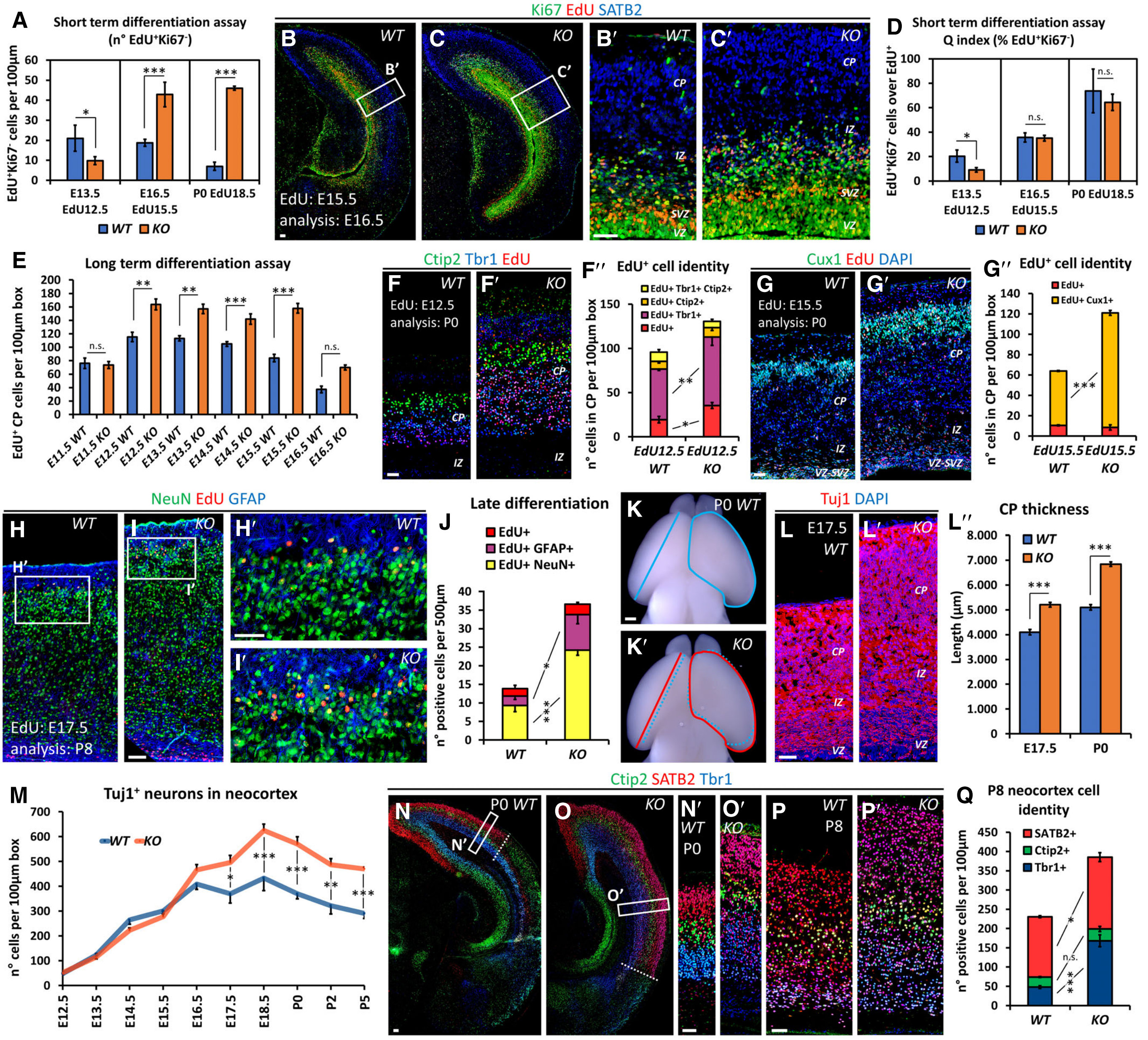

Figure EV4.

© 2020 The Authors

Figure EV4. Delayed neurogenesis and posterior cortical thickening in Nr2f1-deficient mice.

A Graph showing the average number of EdU^+Ki67^- differentiating cells in *WT* (blue columns) and *KO* (orange columns) cortices quantified 24 h after EdU injection. Delayed at E13.5, neurogenesis in *KO* brains increases significantly from E16.5 onwards. $n \geq 4$ sections from $n = 2$ brains.

B–C′ Ki67 (green), EdU (red), and Satb2 (blue; cortical callosal neurons) triple IF of E16.5 *WT* (B, B′) and mutant (C, C′) embryos, injected 24 h before with EdU. Differentiating cells ($EdU^+Satb2^+Ki67^-$) can be recognized as they migrate toward the CP.

D Short-term EdU injection as in (A), after re-normalization on the total number of EdU^+ cells, to take account of the increased size of progenitor pool in mutant brains compared to *WT*. $n \geq 4$ sections from $n = 2$ brains.

E Average number of EdU^+ cells in the LP of control (blue columns) and mutant (orange columns) embryos at P0 upon EdU injection at the indicated stages. Mutant cortices are characterized by a higher density of differentiating neurons at mid-late corticogenesis. $n \geq 4$ sections from $n = 2$ brains.

F–F″ Ctip2 (green), Tbr1 (blue), and EdU (red; injected at E12.5) triple IF of P0 *WT* (F) and *KO* (F′) brains showing a high number of E12.5-born $Tbr1^+EdU^+$ neurons in lower layers of mutant cortices. Graph in (F″) quantifies the average number of EdU^+ neurons expressing distinct laminar markers (Tbr1 and Ctip2) in *WT* and *KO* embryos injected with EdU at E12.5. $n \geq 4$ sections from $n = 2$ brains.

G–G″ Cux1 (green) and EdU (red; injected at E15.5) double IF of P0 *WT* (G) and *KO* (G′) brains showing a high number of E15.5-born $Cux1^+EdU^+$ neurons in superficial layers of mutant cortices. Quantification of E15.5-born $Cux1^+$ superficial neurons in *WT* and *KO* embryos is shown in (G″). $n \geq 4$ sections from $n = 2$ brains.

H–J NeuN (green; neurons), EdU (red), and GFAP (blue; glia) triple IF of *WT* (H, H′) and *KO* (I, I′) animals injected at E17.5 and analyzed at P8. The average number of EdU^+NeuN^+ neurons or of EdU^+GFAP^+ glial cells is quantified in (J). $n \geq 4$ sections from $n = 2$ brains.

K, K′ Representative images of P0 *WT* (K) and *KO* (K′) brains, showing slight elongation of posterior hemispheres (compare red lines with blue ones).

L, M Tuj1 (red) IF of *WT* (L) and *KO* (L′) cortices. Note the increased thickness of *KO* IZ and CP compared to *WT* ones. Graph in (L″) indicates CP thickness measured at E17.5 and P0 in the posterior cortical region of control (blue) and mutant (orange) animals. The average number of $Tuj1^+$ differentiating neurons in posterior-most CP is quantified from E12.5 to P5 in (M). L″: $n = 2$ brains per genotype/age; M: $n \geq 4$ sections from $n = 2$ brains.

N–Q Ctip2 (green), Tbr1 (blue), and Satb2 (red) triple IF of *WT* (N,N′,P) and *KO* (O,O′,P′) posterior cortices at P0 and P8, as indicated, and quantified in (Q). $n \geq 4$ sections from $n = 2$ brains.

Data information: Nuclei (blue) were stained with DAPI. Data are represented as means ± SEM. Two-way ANOVA (*$P < 0.05$; **$P < 0.01$; ***$P < 0.001$). Scale bars: 50 μm. CP: cortical plate; IZ: intermediate zone; SVZ: subventricular zone; VZ: ventricular zone.

© 2020 The Authors

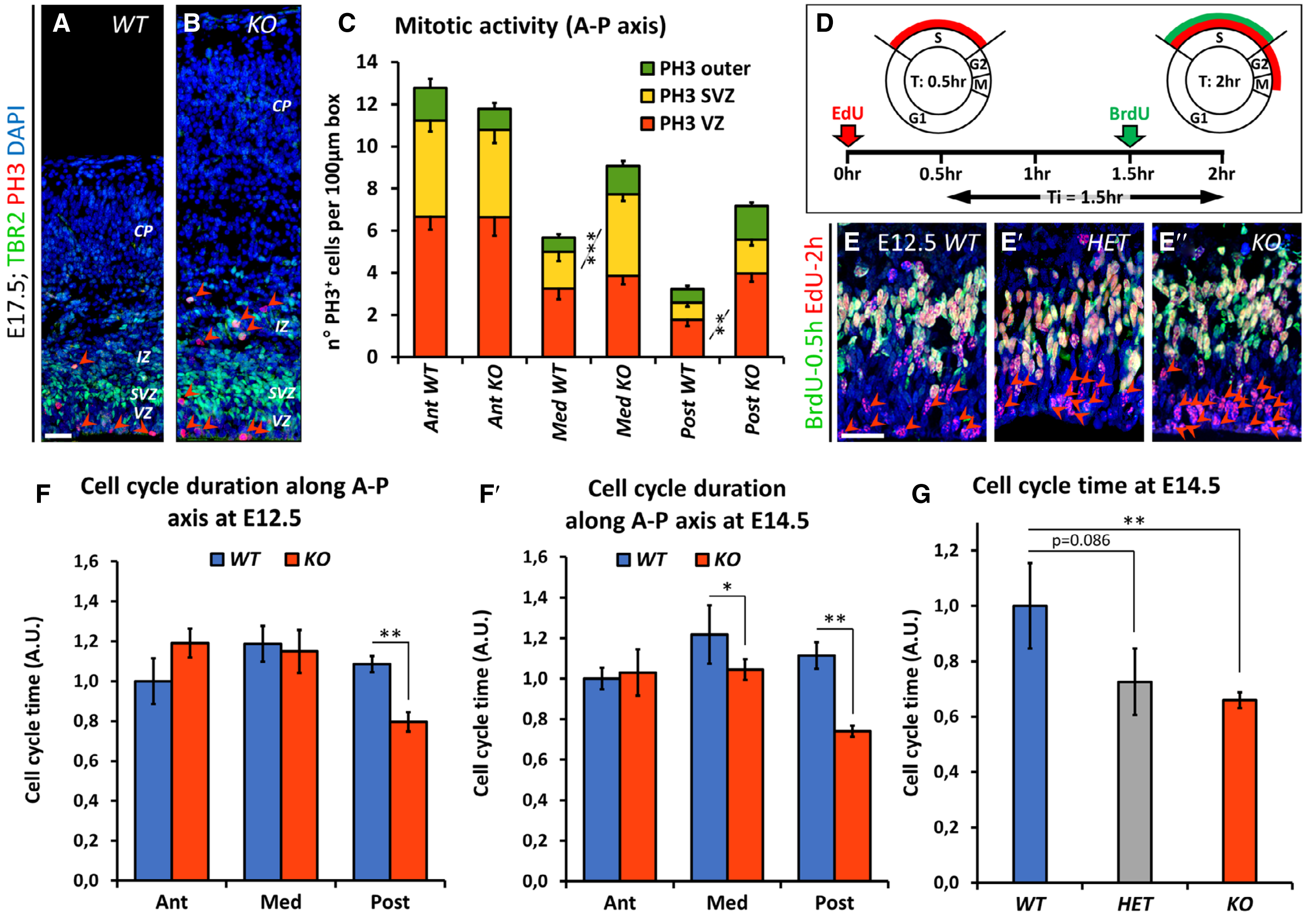

Figure EV5. Regional control of cell cycle dynamics.

A, B Tbr2 (green; IPs) and PH3 (red; mitotic figures) double IF of E17.5 *WT* and *KO* posterior lateral pallia (LPs). Arrowheads in (A, B) point to PH3+ mitotic NPs.

C Average number of dividing cells in the LP of control (*WT*) and mutant (*KO*) animals in different regions along the A-P axis, as indicated. E17.5 was chosen as a developmental age displaying abundant basal progenitor populations. Values of dividing aRGs, IPs, and bRGs are indicated in orange, yellow, and green, respectively. $n \geq 3$ brains.

D Experimental set-up for cell cycle time quantification by double EdU/BrdU injection (adapted from Martynoga *et al*, 2005). Ti corresponds to an interval time between EdU and BrdU injections. For detailed information, see Methods in the Appendix File.

E–E″ BrdU (green) and EdU (red) double IF of *WT*, *HET*, and *KO* E12.5 ventricular zone of LPs. Single EdU+ cells (leaving fraction) that exit the S-Phase during the 2-h injection period move within the VZ surface (red arrowheads), while double EdU/BrdU label S-phase cells reside in a more basal VZ position, next to the SVZ.

F, F′ Cell cycle duration along the A-P axis of E12.5 (F) and E14.5 (F′) LP in mutant (*KO*) embryos and their control (*WT*) littermates. Cell cycle is accelerated (i.e., has a shorter duration) in NPs of the posterior-most region of the mutant cortex, where Nr2f1 is expressed at high levels in control littermates. $n \geq 3$ brains per age/genotype. Cell cycle time has been expressed in arbitrary units (A.U.) upon normalization on the WT Ant duration.

G Cell cycle duration of E14.5 *WT*, *HET*, and *KO* LPs, showing a decreasing trend of cell cycle duration in *HET* animals and a significant decrease in *KO* ones compared to *WT* controls. $n \geq 2$ brains.

Data information: Nuclei (blue) were stained with DAPI. In (C, F-G), the number of positive cells was quantified in 100 µm-width boxes, randomly placed across the LP. In graphs, data are represented as means ± SEM. Two-way ANOVA (*$P < 0.05$; **$P < 0.01$; ***$P < 0.001$). Scale bars: 50 µm. Ant: anterior; CP: cortical plate; IZ: intermediate zone; Med: medial; Post: posterior; SVZ: subventricular zone; VZ: ventricular zone.

 © 2020 The Authors
